# Variability and Magnitude of Choline Levels Across the Psychosis Spectrum: A Meta-analysis

**DOI:** 10.1093/schbul/sbag069

**Published:** 2026-05-11

**Authors:** Jack B Fanshawe, Cameron J Watson, Valentina Mancini, Matthew Cohen, Doğukan Koçyiğit, Rijul Bohra, Chambrez-Zita Zauchenberger, Ioana Varvari, Anna Farid, Violeta Perez-Rodriguez, George Gifford, Toby Pillinger, Katherine Beck, Sameer Jauhar, Philip McGuire, Robert A McCutcheon

**Affiliations:** Department of Psychiatry, University of Oxford, Oxford OX3 7JX, United Kingdom; TUNE-UP service, Oxford Health NHS Foundation Trust, Oxford OX3 7JX, United Kingdom; Social, Genetic and Developmental Psychiatry Centre, Institute of Psychiatry, Psychology and Neuroscience, King's College London, London SE5 8AF, United Kingdom; Department of Psychosis Studies, Institute of Psychiatry, Psychology and Neuroscience, King’s College London, London SE5 8AF, United Kingdom; South London and Maudsley NHS Foundation Trust, London SE5 8AZ, United Kingdom; Department of Psychiatry, University of Oxford, Oxford OX3 7JX, United Kingdom; TUNE-UP service, Oxford Health NHS Foundation Trust, Oxford OX3 7JX, United Kingdom; Wellcome Centre for Integrative Neuroimaging (WIN) & MRC Brain Network Dynamics Unit (BNDU), Nuffield Department of Clinical Neurosciences, University of Oxford, Oxford OX3 9DU, United Kingdom; South London and Maudsley NHS Foundation Trust, London SE5 8AZ, United Kingdom; Department of Psychological Medicine, Institute of Psychiatry, Psychology and Neuroscience, King’s College London, London SE5 8AF, United Kingdom; Department of Psychiatry, University of Oxford, Oxford OX3 7JX, United Kingdom; Department of Psychiatry, Hacettepe University, Ankara 06230, Türkiye; South London and Maudsley NHS Foundation Trust, London SE5 8AZ, United Kingdom; Department of Psychiatry, University of Oxford, Oxford OX3 7JX, United Kingdom; TUNE-UP service, Oxford Health NHS Foundation Trust, Oxford OX3 7JX, United Kingdom; Department of Psychiatry, University of Oxford, Oxford OX3 7JX, United Kingdom; TUNE-UP service, Oxford Health NHS Foundation Trust, Oxford OX3 7JX, United Kingdom; Department of Psychosis Studies, Institute of Psychiatry, Psychology and Neuroscience, King’s College London, London SE5 8AF, United Kingdom; Department of Psychosis Studies, Institute of Psychiatry, Psychology and Neuroscience, King’s College London, London SE5 8AF, United Kingdom; Department of Psychiatry, University of Oxford, Oxford OX3 7JX, United Kingdom; Department of Psychosis Studies, Institute of Psychiatry, Psychology and Neuroscience, King’s College London, London SE5 8AF, United Kingdom; TUNE-UP service, South London and Maudsley NHS Foundation Trust, London SE5 8AZ, United Kingdom; Department of Psychosis Studies, Institute of Psychiatry, Psychology and Neuroscience, King’s College London, London SE5 8AF, United Kingdom; South London and Maudsley NHS Foundation Trust, London SE5 8AZ, United Kingdom; Department of Psychiatry, Imperial College London, London W12 0NN, United Kingdom; Department of Psychiatry, University of Oxford, Oxford OX3 7JX, United Kingdom; Department of Psychiatry, University of Oxford, Oxford OX3 7JX, United Kingdom; TUNE-UP service, Oxford Health NHS Foundation Trust, Oxford OX3 7JX, United Kingdom; Department of Psychosis Studies, Institute of Psychiatry, Psychology and Neuroscience, King’s College London, London SE5 8AF, United Kingdom; Department of Psychiatry, Imperial College London, London W12 0NN, United Kingdom

**Keywords:** MRS, spectroscopy, cholinergic, schizophrenia, treatment resistance, clinical high risk

## Abstract

**Background and Hypothesis:**

Animal, post-mortem, and pharmacological studies suggest altered choline levels in psychosis. Proton magnetic resonance spectroscopy (^1^H-MRS) may allow in vivo investigation of the cholinergic system. This meta-analysis examined the magnitude and variability of ^1^H-MRS choline across the psychosis spectrum.

**Study Design:**

Following a pre-registered protocol (CRD42023403879), we searched MEDLINE, EMBASE, and PsycINFO for studies reporting ^1^H-MRS choline levels in individuals with psychosis, clinical high risk (CHR) states, and healthy controls. Standardized mean differences (SMDs) and log coefficient of variation ratios were pooled using random-effects meta-analysis, stratified by brain region and clinical subgroup. Meta-regression examined associations with imaging and clinical parameters.

**Study Results:**

We included 165 studies comprising 5178 patients and 4269 controls. Choline levels were elevated in people with psychosis in the anterior cingulate cortex (ACC: SMD = 0.23, CI 0.12–0.33), medial prefrontal cortex (mPFC: SMD = 0.12, CI 0.01-0.23), and dorsolateral prefrontal cortex (dlPFC: SMD = 0.15, CI 0.01-0.28). Elevations were also seen in CHR cohorts (ACC: SMD 0.19, CI 0.03-0.36; mPFC: SMD 0.36, CI 0.16-0.57), with the largest effects in treatment-resistant schizophrenia (ACC: SMD 0.65, CI 0.38-0.91; dlPFC: SMD 1.13, CI 0.65-1.60). Increased choline variability was observed in the dlPFC in psychosis cohorts and the mPFC and temporal lobe in CHR groups.

**Conclusions:**

This is the largest meta-analysis of ^1^H-MRS choline in psychosis to date. Increased variability in psychosis and greater differences in treatment-resistant cohorts suggest increased choline levels might identify a subgroup who do not respond to dopamine antagonist treatment.

## Introduction

Schizophrenia is characterized by delusions, hallucinations, disorganized thoughts, and cognitive dysfunction, with an estimated lifetime prevalence of 7.49 per 1000 people.^1^ The underlying pathophysiology is complex, with no single mechanism explaining the range of associated biological and behavioral abnormalities.^2,3^

Proton magnetic resonance spectroscopy (^1^H-MRS) enables the in vivo quantification of various metabolites in the brain.^4^ Its non-invasive nature, and suitability for both patient and preclinical studies, lends it potential as a translational biomarker for pathophysiological research and drug target development.

Choline-containing compounds have numerous functions: choline is an integral part of neuronal membrane phospholipids,^5^ is acylated to form the neurotransmitter acetylcholine,^6^ and is required for DNA methylation.^7^ Choline creates a singlet peak on ^1^H-MRS, allowing for ease of measurement and has been one of the most reported metabolites in ^1^H-MRS studies of psychosis. Raised ^1^H-MRS choline levels seen in people with schizophrenia have traditionally been proposed to represent worsening membrane breakdown, increased cellular turnover and glial overactivation in response to neuroinflammation.^8,9^ These changes are seen in other neuroinflammatory disorders,^8^ while choline is also elevated after traumatic brain injury.^10^ Another possibility is that choline levels partially reflect synaptic choline levels. Evidence from functional ^1^H-MRS has highlighted changes in choline concentrations during cognitive tests thought to induce cholinergic signaling^11,12^; ^1^H-MRS signal also strongly correlates with free choline but not membrane-bound phosphtidylcholine.^13^

A range of animal, postmortem, and pharmacological studies provide indirect evidence of cholinergic dysregulation in schizophrenia.^14-16^ Anticholinergic medication challenges elicit psychotic symptoms in both healthy controls and schizophrenia, while higher anticholinergic burden is significantly associated with cognitive deficits in psychosis.^17,18^ Muscarinic agonism has also recently been shown to be an effective treatment of schizophrenia.^19^ More direct evidence is possible using positron emission tomography (PET). While there are few PET studies, these have suggested reduced muscarinic receptor availability in schizophrenia.^20,21^ Whilst ^1^H-MRS cannot directly quantify receptors, unlike PET it is non-invasive and is economically more viable, lending it potential to provide information regarding the cholinergic system across psychosis spectrum disorders.

Postmortem findings in individuals with schizophrenia suggest that a reduction in M1 receptor availability is limited to a subset of cases, with no difference between all schizophrenia cases and controls.^22^ Meta-analysis of choline variability between cases and controls represents an opportunity to assess whether subgroups with varying levels of choline dysfunction are present in vivo. Identifying subgroups associated with distinct pathophysiological mechanisms may help explain highly variable symptomatology,^23^ clinical course,^24^ and treatment response^25^ in psychosis, and ultimately facilitate more precise treatment and prognostication.

This study aimed to explore choline concentration differences in terms of magnitude and variability between patients and controls across the psychosis spectrum for all available brain regions.

## Methods

### Search Strategy and Study Selection

This systematic review and meta-analysis was pre-registered on Prospero (CRD42023403879) and conducted in line with PRISMA-S guidance.^26^ MEDLINE, EMBASE, and PsycINFO databases were searched up to December 21, 2024 for peer reviewed original research in English; full search terms are found in the Supplementary material. Reference lists of included articles were also examined to identify further works. Duplicate articles were identified automatically using Rayyan software,^27^ followed by manual deduplication by comparing article citations.

Inclusion criteria consisted of studies reporting ^1^H-MRS choline values for patients with psychosis (including schizophrenia, treatment-resistant schizophrenia, schizoaffective disorder, and bipolar disorder with psychosis), or at clinical high risk (CHR) for psychosis (as defined by each study) and healthy controls. Exclusion criteria included studies specifically reporting on individuals with comorbid substance misuse disorder, studies reporting choline only as a ratio of other metabolites (with the exception of creatine), or studies reporting on post-mortem samples. When studies reported longitudinal results, the baseline time point was used. For separate studies with overlapping patient groups, the largest cohort was extracted.

Two reviewers independently evaluated articles by sequentially reviewing titles and abstracts in parallel (J.B.F, M.C., C.Z., V.M., and R.B). Disagreement over inclusion was arbitrated by a third reviewer. Articles marked for full-text screening were obtained through searches in online catalogues and university libraries. Study authors were contacted directly for unretrievable articles. Full texts were examined in the same manner as titles and abstracts.

### Data Extraction and Risk of Bias

Data were extracted by reviewers (M.C., C.Z., V.M., R.B., C.J.W., and I.V.) onto a pre-piloted extraction sheet, with a second check of data performed by J.B.F. Extracted data included choline mean and standard deviation, full width and half maximum (FWHM), Cramer-Rao Lower Bound (CRLB), signal:noise ratio (SNR), magnetic field strength, sex, age, and medication details. Data were analyzed separately for the following diagnostic groups: CHR, established psychosis (first episode psychosis, unspecified psychosis, and schizophrenia), and treatment-resistant schizophrenia. These groups were also combined to create a combined psychosis group.

Data were extracted for the following anatomical locations; anterior cingulate cortex (ACC), centrum semiovale, cerebellum, corpus callosum, dorsolateral prefrontal cortex (dlPFC), unspecified frontal lobe (not white matter) and medial prefrontal cortex (mPFC), frontal white matter, global grey matter, global white matter, hippocampus, occipital lobe, parietal lobe, posterior cingulate cortex, striatum, temporal lobe, and thalamus. Where required, standard errors and 95% CIs were converted to SDs as per the Cochrane handbook.^28^ Risk of bias was assessed independently by two trained authors (AF and VP-R) for all included studies using the Newcastle–Ottawa Scale for case–control studies.^29^ Each study was classified as having low, moderate, or high overall risk of bias based on performance across predefined domains. In sensitivity analyses, meta-analyses were repeated excluding studies rated as high risk of bias, and subsequently restricting analyses to studies rated as low risk of bias only, to assess the robustness of the primary findings.

### Meta-Analysis

Effect sizes were calculated through standardized mean differences (SMD). With high heterogeneity expected from mixed diagnoses, differing medication status and correction methods, a random-effects, inverse-weighted variance model was used to pool effect sizes across studies. Analyses were conducted on both the combined psychosis group and the CHR, established psychosis, and TRS subgroups. Meta-analyses were conducted using the metafor package^30^ in R (Version 2023.12.0 + 369). Anatomical locations were analyzed if values were reported by ≥2 studies. Publication bias was assessed by visual inspection of funnel plots and Egger’s regression test where results were available for >10 studies.^31^  *I*^2^ values were calculated to quantify between-study inconsistency.

To compare variability between treatment and control groups while adjusting for systematic scaling of SD with group mean, we computed the log-Coefficient of Variation Ratio (CVR) for each study. Log-transforming CVR improves normality of the sampling distribution and yields a symmetric metric for relative changes in variability by adjusting the variability ratio (VR) for mean differences between groups. This was calculated using the escalc function of the metafor package.^30^ VR results are presented in Tables S4-S7.

### Meta-Regressions and Sensitivity Analyses

Meta-regressions were conducted to examine the influence of technical, clinical, and demographic moderators on choline effect sizes in the combined psychosis cohort. Prespecified univariate random-effects meta-regressions were performed using the metafor package where moderator data were available for ≥5 independent cohorts.^30^ Technical moderators included scanner field strength, acquisition sequence (PRESS vs non-PRESS), echo time, repetition time, voxel volume, MRI vendor, SNR, FWHM, and year of publication. Methodological factors examined included metabolite referencing method (creatine vs non-creatine), application of motion exclusion criteria, and overall risk of bias. Clinical and demographic moderators included age, sex, medication status (percentage medication-free and medication-naïve), antidepressant use, substance misuse, and symptom severity measured using PANSS total, positive, and negative subscales. Where available, exploratory analyses also examined the difference in mean intelligence quotient (IQ) between psychosis and control groups as a moderator.

Sensitivity analyses were undertaken to assess robustness. Restriction-based analyses stratified studies by scanner field strength, acquisition sequence, MRI vendor, metabolite referencing method, motion exclusion status, and risk of bias category. Exclusion-based analyses sequentially removed studies rated as high risk of bias, high or medium risk of bias, reporting substance misuse, not explicitly excluding antidepressant use, or not excluding psychiatric comorbidity.

## Results

**Figure 1:**
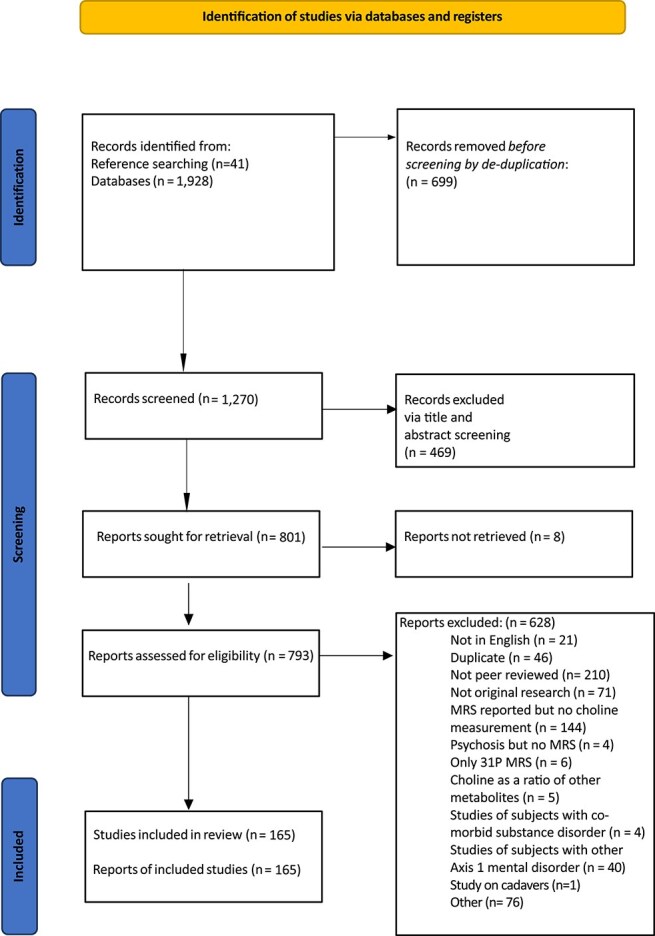
PRISMA Flow Chart.

A total of 1928 titles and abstracts were identified in our database search; 699 were duplicates. A further 41 studies were identified via handsearching references of included studies and previous reviews. Full texts for 801 records were screened and 165 were found to meet inclusion criteria, including 5178 patients and 4269 healthy controls (Figure 1 and Tables S2 and S3).

### Overall Findings

**Figure 2:**
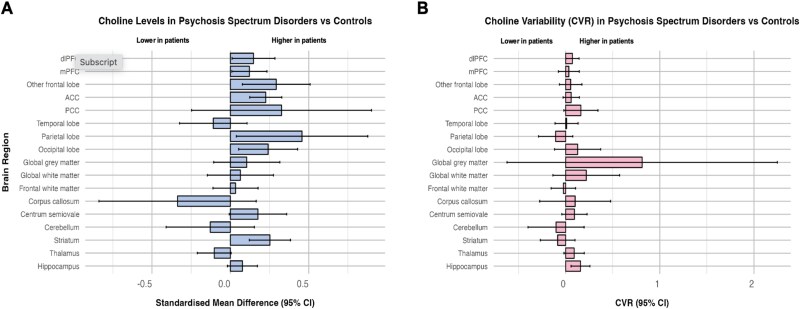
(A) Magnitude of difference (SMD) and (B) CVR in choline levels by brain region between controls and patients with psychosis spectrum disorders. Positive SMD indicates higher choline levels in cases compared to controls. Positive CVR indicates higher choline variability in cases compared to controls. Abbreviations: ACC, anterior cingulate cortex; dlPFC = dorsolateral prefrontal cortex; mPFC, medial prefrontal cortex; PCC, posterior cingulate cortex.

Meta analytic estimates with ≥2 studies for the combined psychosis group were available from 17 anatomical regions and are displayed in Figure 2. Statistically significant raised choline levels in people with a psychosis spectrum diagnosis compared with healthy controls were evidenced in the ACC (SMD 0.23, 95% CI, 0.12-0.33, *I*^2^ = 53.4%, *P* < .001), mPFC (SMD 0.12, 95% CI, 0.01-0.23, *I*^2^ = 22.6%, *P* = .034), other frontal lobe (SMD 0.29, 95% CI, 0.08-0.51, *I*^2^ = 39.0%, *P* < .001), dlPFC (SMD 0.15, 95% CI, 0.01-0.28, *I*^2^ = 49.3%, *P* = .034), occipital lobe (SMD 0.24, 95% CI, 0.05-0.43, *I*^2^ = 0.0%, *P* = .012), parietal lobe (SMD 0.46, 95% CI, 0.04-0.88, *I*^2^ = 73.1%, *P* = .03), and the striatum (SMD 0.25, 95% CI, 0.12-0.38, *I*^2^ = 32.0%, *P* < .001). SMD results for all areas are summarized in Table 1. Forest plots for all brain regions are presented in Figure S2A-P.

**Table 1:** SMD Meta-analysis Results Summary for Psychosis Spectrum Disorders in All Brain Regions

Psychosis spectrum disorders										
Region	Cohorts	Cases	Controls	SMD	Lower 95% CI	Upper 95% CI	*P*-value	*I* ^2^ (%)	Eggers statistic	Eggers *P*-value
ACC	65	2010	1676	0.23	0.12	0.33	<.001	53.4	0.98	.33
dlPFC	39	1013	939	0.15	0.01	0.28	.034	49.3	1.31	.20
Occipital lobe	10	211	241	0.24	0.05	0.43	.012	0.0	−0.74	.48
Hippocampus	39	1070	868	0.08	−0.02	0.17	.114	5.3	1.12	.27
mPFC	27	1006	828	0.12	0.01	0.23	.034	22.6	1.09	.29
Frontal white matter	24	566	521	0.03	−0.11	0.18	.653	23.9	0.28	.78
Thalamus	35	1033	914	−0.10	−0.21	0.01	.061	22.8	−0.49	.63
Striatum	36	798	696	0.25	0.12	0.38	<.001	32.0	2.00	.05
Cerebellum	7	158	131	−0.13	−0.41	0.15	.371	25.5	N/A	N/A
Temporal lobe	22	567	440	−0.11	−0.32	0.11	.322	60.3	0.60	.55
Other frontal lobe	16	322	288	0.29	0.08	0.51	<.001	39.0	−2.56	.02
Parietal lobe	10	205	172	0.46	0.04	0.88	.033	73.1	0.78	.46
Centrum semiovale	6	218	244	0.18	−0.01	0.36	.061	0.0	N/A	N/A
Global grey matter	3	177	170	0.11	−0.11	0.32	.331	0.0	N/A	N/A
Global white matter	3	177	170	0.06	−0.15	0.28	.551	0.0	N/A	N/A
Corpus callosum	3	43	25	−0.34	−0.84	0.17	.188	0.0	N/A	N/A
PCC	4	116	144	0.33	−0.25	0.90	.265	77.9	N/A	N/A

Abbreviations: ACC = anterior cingulate cortex; dlPFC = dorsolateral prefrontal cortex; mPFC = medial prefrontal cortex; PCC = posterior cingulate cortex; SMD = standardized mean difference.

### Results Across the Spectrum of Psychoses

**Figure 3:**
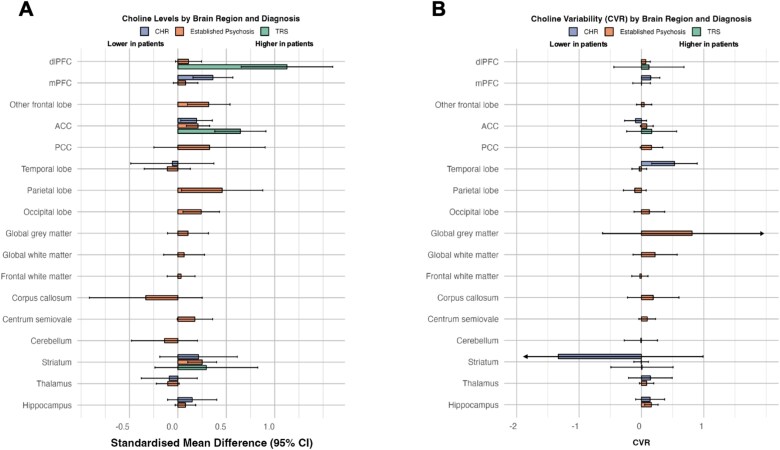
(A) Magnitude of difference (SMD) and (B) CVR in choline levels by brain region between controls and CHR, established psychosis and TRS. Positive SMD indicates higher choline levels in cases compared to controls. Positive CVR indicates higher choline variability in cases compared to controls. Abbreviations: ACC = anterior cingulate cortex; CHR = clinical high risk; CVR = log coefficient of variation ratio; dlPFC = dorsolateral prefrontal cortex; mPFC = medial prefrontal cortex; PCC = posterior cingulate cortex; TRS = treatment-resistant schizophrenia.

In clinical high-risk populations, meta-analytic results with ≥2 studies were available from 6 anatomical regions (Figure 3). Results from the mPFC (SMD 0.36, 95% CI, 0.16-0.57 *I*^2^ = 3.5%, *P* < .001) and the ACC (SMD 0.19, 95% CI, 0.03-0.36, *I*^2^ = 0.3%, *P* = .02) evidenced statistically significant elevations of choline in cases versus controls. Results from all areas are presented in Table S8.

In patients with established psychosis, meta-analytical results with ≥2 studies were available from 17 anatomical regions comparing choline levels with controls (Figure 3). A significant increase of choline was evidenced in the occipital lobe (SMD 0.24, 95% CI, 0.05-0.43, *I*^2^ = 0.0%, *P* = .012), other frontal lobe (SMD 0.32, 95% CI, 0.10-0.54, *I*^2^ = 39.0%, *P* = .004), ACC (SMD 0.21, 95% CI, 0.09-0.33, *I*^2^ = 59.5%, *P* < .001), parietal lobe (SMD 0.46, 95% CI, 0.04-0.88, *I*^2^ = 73.1%, *P* = .03), and the striatum (SMD 0.25, 95% CI, 0.10-0.40, *I*^2^ = 40.3, *P* < .001). Results from all areas are presented in Table S9.

In patients with treatment-resistant schizophrenia choline levels were significantly elevated compared with healthy controls in the ACC (SMD 0.65, 95% CI, 0.38-0.91, *I*^2^ = 0.0%, *P* < .001) and the dlPFC (SMD 1.13, 95% CI, 0.65-1.60, *I*^2^ = 0.0%, *P* < .001), but not in the striatum (SMD 0.29, 95% CI, −0.24 to 0.82, *I*^2^ = 55.6%, *P* = .278) (Figure 3 and Table S10).

Forest plots for all brain regions and diagnostic groups are presented in Figures S3A-F, S4A-P, and S5A-C.

### Variability

In the combined psychosis group, a statistically significant increase in variability was evident in the dlPFC (CVR 0.07, 95% CI, 0.00-0.14, *I*^2^ = 0.0%, *P* = .045) and hippocampus (CVR 0.16, 95% CI, 0.06-0.26, *I*^2^ = 48.3%, *P* = .002) (Figure 2 and Table 2). In the CHR population, significantly increased variability in cases was evident in the temporal lobe (CVR 0.53, 95% CI, 0.16-0.90, *I*^2^ = 0.0%, *P* = .004), and the mPFC (CVR 0.15, 95% CI, 0.00-0.86, *I*^2^ = 37.5%, *P* = .05). All results are presented in Table S11. In the established psychosis cohort significantly increased variability in patients was evident in the hippocampus (CVR 0.16, 95% CI, 0.05-0.27, *I*^2^ = 50.3%, *P* = .004). The remaining regions were not statistically significant (Table S12). In the TRS population none of the regions showed significant differences in variability between cases and controls (Figure 3 and Table S13).

**Table 2:** CVR Meta-analysis Results Summary for Psychosis Spectrum Disorders in All Brain Regions

Psychosis spectrum disorders								
Region	Cohorts	Cases	Controls	CVR	Lower 95% CI	Upper 95% CI	*P*-value	*I* ^2^ (%)
ACC	65	2010	1676	0.06	−0.03	0.15	.184	64.2
dlPFC	39	1013	939	0.07	0.00	0.14	.045	0.0
Occipital lobe	10	211	241	0.13	−0.12	0.37	.310	61.7
Hippocampus	39	1070	868	0.16	0.06	0.26	.002	48.3
mPFC	27	1006	828	0.03	−0.08	0.15	.553	55.7
Frontal white matter	24	566	521	−0.03	−0.16	0.11	.708	47.0
Thalamus	35	1033	914	0.09	−0.02	0.20	.101	56.6
Striatum	36	798	696	−0.09	−0.27	0.10	.364	81.0
Cerebellum	7	158	131	−0.10	−0.40	0.20	.502	63.2
Temporal lobe	22	567	440	0.01	−0.11	0.13	.878	32.7
Other frontal lobe	16	322	288	0.05	−0.07	0.17	.389	0.0
Parietal lobe	10	205	172	−0.11	−0.29	0.08	.250	25.7
Centrum semiovale	6	218	244	0.09	−0.04	0.23	.172	0.0
Global grey matter	3	177	170	0.81	−0.62	2.25	.267	97.8
Global white matter	3	177	170	0.22	−0.14	0.57	.226	78.0
Corpus callosum	3	43	25	0.10	−0.28	0.48	.600	0.0
PCC	4	116	144	0.16	−0.02	0.34	.081	0.0

Abbreviations: ACC = anterior cingulate cortex; dlPFC = dorsolateral prefrontal cortex; mPFC = medial prefrontal cortex; PCC = posterior cingulate cortex.

### Effects of Antipsychotic Medication Status

Both higher antipsychotic medication free percentage and antipsychotic medication naive percentage were found to be significantly associated with reduced case vs control effect size in the dlPFC (Figures S20 and S22). However, the inverse was evident, in the hippocampus with higher medication free percentage and medication naïve percentage being associated with higher case control effect size (Figures S21 and S23; Tables S19 and S20). Neither medication free nor medication naive percentage were significant for any other regions on meta-regression. On subgroup analysis versus healthy controls, the choline magnitude in the dlPFC was statistically significantly lower in both the medication free (SMD −0.28, 95% CI, −0.48 to −0.09, *P* = .004), and medication naïve cohorts (SMD −0.26, 95% CI, −0.49 to −0.03, *P* = .028), whereas in the antipsychotic treated cohort choline magnitude was higher in cases versus healthy controls (SMD 0.28, 95% CI, 0.08-0.35, *P* = .001, Figure S1). The magnitude difference between cases and healthy controls was not significant in any subgroups in the hippocampus (Figure S1 and Table S23). Only four TRS studies reported the percentage of patients treated with clozapine making meta-regression infeasible,^32-35^ Tarumi et al., reported TRS results for patients not on clozapine and reported comparable results to studies reporting results for clozapine treated patients (Figure S5A-C).

### Other Meta-Regressions and Publication Bias

Meta-regressions examining demographic, technical, and clinical moderators demonstrated largely region-specific associations. A greater proportion of male participants was associated with reduced case–control differences in the occipital lobe, hippocampus, and frontal white matter (Table S18). No statistically significant effect of smoking status was evident (Table S21). Higher CRLB values were associated with reduced effect sizes in the dlPFC, mPFC, and ACC (Table S22). There were no consistent associations observed for age, signal-to-noise ratio, publication year, FWHM, magnetic field strength, repetition time, echo time, or other acquisition parameters (Tables S14-S16, S24-S26). Restriction-based sensitivity analyses supported the robustness of the primary findings, although some regions no longer reached statistical significance when restricting analyses to creatine-referenced studies. Leave-one-out analyses did not identify any disproportionately influential studies (Table S31). Several regions lost statistical significance when restricting analyses to creatine-referenced studies, formal subgroup comparisons indicated that creatine referencing did not significantly moderate SMD for any region apart from the striatum (Table S34). No consistent moderation was observed by field strength, MRI vendor, acquisition sequence, or motion exclusion criteria (Tables S29-S30, S32-S34).

In contrast, we identified clinically relevant moderators with regionally specific effects. No statistically significant effect of antidepressant medication use percentage was evidence (Table S28). While PANSS total scores were not associated with choline effect sizes, greater PANSS positive symptoms was associated with lower effect sizes in the ACC and higher effect sizes in the thalamus. PANSS negative symptom severity was positively associated with ACC effect sizes (Table S35). Meta-regression of cognitive differences between cases and controls demonstrated that larger IQ differences, reflecting greater cognitive impairment, were associated with greater ACC choline effect sizes (Table S36).

Visual inspection of funnel plots and Egger’s test revealed no evidence of publication bias except in studies of the other frontal lobe region (Table 1 and Figures S6 and S15).

### Risk of Bias

Across the 165 included studies, 18 were rated as low risk of bias, 79 as moderate risk, and 68 as high risk of bias (Figure S27 and Table S37). The most common sources of bias related to representativeness of the study population, limited matching or adjustment for key clinical confounders, and heterogeneity in reporting of acquisition and processing parameters.

Sensitivity analyses excluding studies rated as high risk of bias, and further restricting analyses to low risk-of-bias studies only (Tables S29 and S30), yielded results that were consistent in direction and broadly similar in magnitude to the primary analyses, indicating that the main findings were robust to study quality.

## Discussion

This systematic review and meta-analysis represent the most comprehensive overview of choline level differences across the psychosis spectrum. Altered choline concentrations are demonstrated across several brain regions in individuals with psychotic disorders compared with healthy controls, with particularly large differences observed in TRS. The increased variability of choline levels in some regions suggests that differences are not universal but possibly restricted to a subgroup. That group differences are greatest in treatment-resistant patients, and that variability in this group is not increased, suggests that this potential subgroup of individuals may overlap with the clinically defined group of individuals with treatment-resistant schizophrenia.

The choline resonance measured by ^1^H-MRS predominantly reflects total choline (tCho), comprising water-soluble choline-containing compounds such as phosphocholine (PCho), glycerophosphocholine (GPC), and free choline.^36^ Much of this signal has historically been interpreted as reflecting membrane phospholipid turnover or breakdown,^37^ and therefore as a potential marker of tissue injury or neuroinflammatory processes.^8^ However, biochemical validation studies indicate that the ^1^H-MRS choline signal correlates strongly with the cytosolic pool of free choline and its soluble derivatives, but not with membrane-bound phosphatidylcholine.^13^ In addition, functional MRS studies demonstrate task-dependent changes in choline concentrations during cognitive engagement of cholinergic circuits, occurring on timescales incompatible with membrane turnover or inflammatory processes.^11,12^ Pharmacological studies further show that enhancement of cholinergic signaling, for example with muscarinic agonists, is associated with reductions in MRS-visible choline.^38^ Together, these findings suggest that the ^1^H-MRS choline resonance reflects a metabolically active choline pool influenced by multiple processes, including membrane phospholipid metabolism and cholinergic demand.

In our study, the regional distribution of elevated choline within the ACC, mPFC, dlPFC, striatum, and parietal cortex overlaps with brain regions in which PET and post-mortem studies demonstrate cholinergic abnormalities in schizophrenia. Significant reductions in M1/M4 muscarinic receptor availability are evident in the striatum and fronto-cingulate cortex, alongside alterations in muscarinic and nicotinic receptor expression across frontal, cingulate, and parietal regions, with association to symptom severity and cognitive dysfunction.^39^

Elevated choline levels in CHR individuals were seen in the mPFC and the ACC. There was also increased variability in CHR individuals in the temporal lobe and mPFC, which suggests that alterations are present only in a subgroup and raises the question as to whether raised choline levels might be restricted to those who subsequently transition to psychosis. This is in contrast to measures of glutamate and dopamine function in which no differences have been observed between those in the at-risk state and controls in terms of either magnitude or variability.^40^ Increases in choline level precede the appearance of N-acetylaspartate (NAA) reductions observed in studies of chronic schizophrenia,^41^ and NAA alterations are not present in the at-risk state,^42^ suggesting an important role in prediction for choline compared with other metabolites measurable with MRS. Given that no treatments currently exist to prevent transition in those with CHR, breakthroughs in the underlying neurobiology are critical to guide treatment trials.^43^

Increased variability in choline levels in the hippocampus and dlPFC in those with psychosis suggests heterogeneous pathophysiology. The increased variability in choline concentrations may reflect the presence of biologically distinct subgroups, including individuals with choline levels within the typical range and others with aberrant choline concentrations. This raises the possibility that choline measures could help to identify subgroups with differing underlying neurobiology. Future work should directly test whether such choline-defined subgroups are associated with clinically relevant features, including cognitive profiles and treatment response, both of which show marked interindividual heterogeneity,^25,44,45^ and potential differential response to cholinergic treatments. The regional localization of increased choline variability within prefrontal and hippocampal regions is notable, as these areas overlap with cognitive and salience-related networks,^46-48^ and with regions showing abnormal muscarinic and nicotinic receptor expression,^39,49^ providing further insight into potential subgroup-specific pathophysiology.

The findings of exacerbated differences in choline levels in those with TRS are consistent with evidence from a recent focused meta-analysis.^50^ Notably, however, in the treatment-resistant subgroup choline variability did not differ from controls, despite markedly elevated mean levels. This pattern suggests that treatment-resistant schizophrenia may represent a more biologically homogeneous subgroup with respect to choline abnormalities. The increased efficacy of clozapine versus other antipsychotics in this group may be in part explained by its ability to mitigate this cholinergic dysfunction.^51^ Clozapine, through its active metabolite N-desmethylclozapine, is a full agonist at the M1 receptor, which, alongside its partial agonism of M2 and M4, is unique among antipsychotics.^52^ Alternatively, raised choline levels in those with TRS may reflect the effect of clozapine itself, analogous to the impact seen on striatal volume.^53^ However, choline differences were similar in those with TRS treated with clozapine compared to those treated with other antipsychotics,^32-35^ and clozapine appears to protect dopaminergic neurons from inflammation-induced damage by inhibiting microglial activation.^54^ Further studies focused on to what extent cholinergic dysfunction may predict treatment resistance and suggest the need for early clozapine use are necessary to explore this hypothesis.

The evidence for the crucial role of the cholinergic system in cognition for those with psychosis is further supported by the significant association between increased anticholinergic burden and worsened cognitive impairment.^17,55^ The emerging success of various muscarinic treatments for psychosis has emphasized the importance of the cholinergic system in these disorders for not just cognition but all symptom types.^19,56^ Cholinergic treatment also appears to reduce psychotic symptoms in other conditions such as Parkinson’s disease^57^ and dementia.^58^

The current study has several limitations. The findings of CHR choline level difference and increased variability are novel, however, the small number of studies and participants limit the interpretation of these findings. The relatively small number of studies recording important quality parameters such as LWHM, CRLB, and SNR highlights the need for future studies to follow clear reporting guidelines. A further limitation is that cognitive outcomes beyond global IQ were assessed using heterogeneous instruments across studies, precluding sufficiently consistent data for quantitative synthesis. Although extensive sensitivity, subgroup, and meta-regression analyses were conducted, substantial heterogeneity remained in some regions, indicating that unmeasured clinical or methodological factors may contribute to variability in choline estimates.

The meta-regressions of medication status and subgroup analyses suggest that antipsychotic medication may increase choline levels in the dlPFC. This could potentially reflect a homeostatic response to the anticholinergic effects of many antipsychotics, or alternatively involve dopaminergic mechanisms given the complex interactions between cholinergic and dopaminergic signaling.^51-53^ However, interpretation of these findings should be made cautiously, as medication-stratified analyses were based on a limited number of studies and demonstrated regionally heterogeneous effects. While effect sizes in medication-free and medication-naïve groups indicated lower choline levels in psychosis in some regions, this pattern was not consistent across the brain, with opposing or null associations evident elsewhere. In addition, the marked elevation of choline observed in treatment-resistant schizophrenia may reflect that illness progression or cumulative disease burden increases choline abnormalities rather than medication effects alone. Further work using longitudinal designs, pharmacological challenges, and PET imaging will be required to disentangle illness-related from treatment-related contributions to the choline MRS signal.

The cholinergic system may be of key importance for the cognitive impairments observed in psychosis,^59^ and future large studies looking at associations between choline levels and specific symptom domains would be of value. It would also be of interest to investigate to what extent choline levels are relevant to cognitive function at a transdiagnostic level.^60^ Further evaluation of choline levels as a biomarker for transition to psychosis in CHR cohorts would also be relevant. In addition to the potential to predict resistance to D2 antagonist treatment and potential response to clozapine, it would be of considerable importance to investigate whether raised choline levels predict a favorable response to the recently approved cholinergic agonist xanomeline/trospium. If validated, future trials of cholinergic treatments could stratify participants based on 1H-MRS defined choline level or, where feasible, direct in vivo measures of cholinergic targets (eg, PET or SPECT).

## Conclusion

In the largest meta-analysis of 1H-MRS choline to date, we find provide evidence of cholinergic differences across the psychosis spectrum. While there appears some evidence that these differences emerge prior to the first episode of psychosis, the greatest choline level differences were seen in treatment-resistant cohorts. Taken together with the finding of greater variability in general cohorts—but not resistant cohorts—this suggests that choline level change might be greatest in a subgroup of individuals who do not respond to standard dopamine blocking treatments. Future work should investigate if raised choline concentrations are a potential predictor of response to clozapine or muscarinic agonist treatments.

## Supplementary Material

sbag069_Supplementary_materials

## Data Availability

All data used in this meta-analysis are available on reasonable request from the corresponding author.

## References

[1] Moreno-Küstner B, Martín C, Pastor L. Prevalence of psychotic disorders and its association with methodological issues. A systematic review and meta-analyses. *PLoS One*. 2018;13:e0195687. 10.1371/journal.pone.0195687 29649252 PMC5896987

[2] McCutcheon RA, Reis Marques T, Howes OD. Schizophrenia-an overview. *JAMA Psychiatry*. 2020;77:201-210. 10.1001/jamapsychiatry.2019.3360 31664453

[3] Jauhar S, Johnstone M, McKenna PJ. Schizophrenia. *Lancet Lond Engl*. 2022;399:473-486. 10.1016/S0140-6736(21)01730-X 35093231

[4] Duarte JMN, Xin L. Magnetic resonance spectroscopy in schizophrenia: Evidence for glutamatergic dysfunction and impaired energy metabolism. *Neurochem Res*. 2019;44:102-116. 10.1007/s11064-018-2521-z 29616444 PMC6345729

[5] Zeisel SH . Choline: Critical role during fetal development and dietary requirements in adults. *Annu Rev Nutr*. 2006;26:229-250. 10.1146/annurev.nutr.26.061505.111156 16848706 PMC2441939

[6] Ueland PM . Choline and betaine in health and disease. *J Inherit Metab Dis*. 2011;34:3-15. 10.1007/s10545-010-9088-4 20446114

[7] Jiang X, Greenwald E, Jack-Roberts C. Effects of choline on DNA methylation and macronutrient metabolic gene expression in In vitro models of hyperglycemia. *Nutr Metab Insights*. 2016;9:11-17. 10.4137/NMI.S29465 27081315 PMC4825771

[8] Chang L, Munsaka SM, Kraft-Terry S, Ernst T. Magnetic resonance spectroscopy to assess neuroinflammation and neuropathic pain. *J Neuroimmune Pharmacol Off J Soc NeuroImmune Pharmacol*. 2013;8:576-593. 10.1007/s11481-013-9460-x PMC3698315 23666436

[9] Oz G, Alger JR, Barker PB, et al. Clinical proton MR spectroscopy in central nervous system disorders. *Radiology*. 2014;270:658-679. 10.1148/radiol.13130531 24568703 PMC4263653

[10] Javaid S, Farooq T, Rehman Z, et al. Dynamics of choline-containing phospholipids in traumatic brain injury and associated comorbidities. *Int J Mol Sci*. 2021;22:11313. 10.3390/ijms222111313 34768742 PMC8583393

[11] Bell T, Lindner M, Mullins PG, Christakou A. Functional neurochemical imaging of the human striatal cholinergic system during reversal learning. *Eur J Neurosci*. 2018;47:1184-1193. 10.1111/ejn.13803 29265530

[12] Lindner M, Bell T, Iqbal S, Mullins PG, Christakou A. In vivo functional neurochemistry of human cortical cholinergic function during visuospatial attention. *PLoS One*. 2017;12:e0171338. 10.1371/journal.pone.0171338 28192451 PMC5305251

[13] Miller BL, Chang L, Booth R, et al. In vivo 1H MRS choline: correlation with in vitro chemistry/histology. *Life Sci*. 1996;58:1929-1935. 10.1016/0024-3205(96)00182-8 8637421

[14] McCutcheon RA, Weber LAE, Nour MM, Cragg SJ, McGuire PM. Psychosis as a disorder of muscarinic signalling: Psychopathology and pharmacology. *Lancet Psychiatry*. 2024;11:554-565. 10.1016/S2215-0366(24)00100-7 38795721

[15] Zavitsanou K, Katsifis A, Mattner F, Huang XF. Investigation of m1/m4 muscarinic receptors in the anterior cingulate cortex in schizophrenia, bipolar disorder, and major depression disorder. *Neuropsychopharmacol Off Publ Am Coll Neuropsychopharmacol*. 2004;29:619-625. 10.1038/sj.npp.1300367 14694353

[16] Gerber DJ, Sotnikova TD, Gainetdinov RR, Huang SY, Caron MG, Tonegawa S. Hyperactivity, elevated dopaminergic transmission, and response to amphetamine in M1 muscarinic acetylcholine receptor-deficient mice. *Proc Natl Acad Sci USA*. 2001;98:15312-15317. 10.1073/pnas.261583798 11752469 PMC65026

[17] Mancini V, Latreche C, Fanshawe J, et al. Anticholinergic burden and cognitive function in psychosis: a systematic review and meta-analysis. *Am J Psychiatry*. 2024;182:349-359. 10.1176/appi.ajp.20240260 40007252

[18] Veselinović T, Vernaleken I, Janouschek H, et al. Effects of anticholinergic challenge on psychopathology and cognition in drug-free patients with schizophrenia and healthy volunteers. *Psychopharmacology*. 2015;232:1607-1617. 10.1007/s00213-014-3794-9 25373869

[19] Brannan SK, Sharon S, Miller AC, et al. Muscarinic cholinergic receptor agonist and peripheral antagonist for schizophrenia. *N Engl J Med*. 2021;384:717-726. 10.1056/NEJMoa2017015 33626254 PMC7610870

[20] Raedler TJ, Knable MB, Jones DW, et al. In vivo determination of muscarinic acetylcholine receptor availability in schizophrenia. *Am J Psychiatry*. 2003;160:118-127. 10.1176/appi.ajp.160.1.118 12505810

[21] Bakker G, Vingerhoets C, Boucherie D, et al. Relationship between muscarinic M1 receptor binding and cognition in medication-free subjects with psychosis. *NeuroImage Clin*. 2018;18:713-719. 10.1016/j.nicl.2018.02.030 29560312 PMC5857491

[22] Scarr E, Cowie TF, Kanellakis S, Sundram S, Pantelis C, Dean B. Decreased cortical muscarinic receptors define a subgroup of subjects with schizophrenia. *Mol Psychiatry*. 2009;14:1017-1023. 10.1038/mp.2008.28 18317461

[23] Helldin L, Mohn C, Olsson A-K, Hjärthag F. Neurocognitive variability in schizophrenia spectrum disorders: relationship to real-world functioning. *Schizophr Res Cogn*. 2020;20:100172. 10.1016/j.scog.2020.100172 32090024 PMC7026276

[24] Habtewold TD, Tiles-Sar N, Liemburg EJ, et al. Six-year trajectories and associated factors of positive and negative symptoms in schizophrenia patients, siblings, and controls: genetic risk and outcome of psychosis (GROUP) study. *Sci Rep*. 2023;13:9391. 10.1038/s41598-023-36235-9 37296301 PMC10256804

[25] McCutcheon RA, Pillinger T, Efthimiou O, et al. Reappraising the variability of effects of antipsychotic medication in schizophrenia: a meta-analysis. *World Psychiatry*. 2022;21:287-294. 10.1002/wps.20977 35524614 PMC9077611

[26] Rethlefsen ML, Kirtley S, Waffenschmidt S, et al. PRISMA-S: an extension to the PRISMA statement for reporting literature searches in systematic reviews. *Syst Rev*. 2021;10:1-19. 33499930 10.1186/s13643-020-01542-z PMC7839230

[27] Ouzzani M, Hammady H, Fedorowicz Z, Elmagarmid A. Rayyan—a web and mobile app for systematic reviews. *Syst Rev*. 2016;5:210. 10.1186/s13643-016-0384-4 27919275 PMC5139140

[28] Higgins JPT, Green S. Cochrane Handbook for Systematic Reviews of Interventions. Cochrane Collaboration, 2011.

[29] Stang A . Critical evaluation of the Newcastle-Ottawa scale for the assessment of the quality of nonrandomized studies in meta-analyses. *Eur J Epidemiol*. 2010;25:603-605. 10.1007/s10654-010-9491-z 20652370

[30] Viechtbauer W . Conducting meta-analyses in R with the metafor package. *J Stat Softw*. 2010;36:1-48. 10.18637/jss.v036.i03

[31] Egger M, Smith GD, Schneider M, Minder C. Bias in meta-analysis detected by a simple, graphical test. *BMJ*. 1997;315:629-634. 10.1136/bmj.315.7109.629 9310563 PMC2127453

[32] Ueno F, Nakajima S, Iwata Y, et al. Gamma-aminobutyric acid (GABA) levels in the midcingulate cortex and clozapine response in patients with treatment-resistant schizophrenia: a proton magnetic resonance spectroscopy (1 H-MRS) study. *Psychiatry Clin Neurosci*. 2022;76:587-594. 10.1111/pcn.13463 36111425

[33] Goldstein ME, Anderson VM, Pillai A, Kydd RR, Russell BR. Glutamatergic neurometabolites in clozapine-responsive and -resistant schizophrenia. *Int J Neuropsychopharmacol*. 2015;18:pyu117. 10.1093/ijnp/pyu117 25603859 PMC4438552

[34] Tarumi R, Tsugawa S, Noda Y, et al. Levels of glutamatergic neurometabolites in patients with severe treatment-resistant schizophrenia: a proton magnetic resonance spectroscopy study. *Neuropsychopharmacol Off Publ Am Coll Neuropsychopharmacol*. 2020;45:632-640. 10.1038/s41386-019-0589-z PMC7021829 31842203

[35] Iwata Y, Nakajima S, Plitman E, et al. Glutamatergic Neurometabolite levels in patients with ultra-treatment-resistant schizophrenia: a cross-sectional 3T proton magnetic resonance spectroscopy study. *Biol Psychiatry*. 2019;85:596-605. 10.1016/j.biopsych.2018.09.009 30389132

[36] Boulanger Y, Labelle M, Khiat A. Role of phospholipase A2 on the variations of the choline signal intensity observed by 1H magnetic resonance spectroscopy in brain diseases. *Brain Res Rev*. 2000;33:380-389. 10.1016/S0165-0173(00)00037-0 11011072

[37] Maddock RJ, Buonocore MH. MR spectroscopic studies of the brain in psychiatric disorders. *Curr Top Behav Neurosci*. 2012;11:199-251. 22294088 10.1007/7854_2011_197

[38] Satlin A, Bodick N, Offen WW, Renshaw PF. Brain proton magnetic resonance spectroscopy (1H-MRS) in Alzheimer’s disease: changes after treatment with xanomeline, an M1 selective cholinergic agonist. *Am J Psychiatry*. 1997;154:1459-1461. 10.1176/ajp.154.10.1459 9326834

[39] Saint-Georges Z, MacDonald J, Al-Khalili R, et al. Cholinergic system in schizophrenia: a systematic review and meta-analysis. *Mol Psychiatry*. 2025;30:3301-3315. 10.1038/s41380-025-03023-y 40394282 PMC12185319

[40] McCutcheon RA, Merritt K, Howes OD. Dopamine and glutamate in individuals at high risk for psychosis: a meta-analysis of in vivo imaging findings and their variability compared to controls. *World Psychiatry Off J World Psychiatr Assoc WPA*. 2021;20:405-416. 10.1002/wps.20893 PMC8429330 34505389

[41] Théberge J, Al-Semaan Y, Drost DJ, et al. Duration of untreated psychosis vs. *N*-acetylaspartate and choline in first episode schizophrenia: a 1H magnetic resonance spectroscopy study at 4.0 tesla. *Psychiatry Res Neuroimaging*. 2004;131:107-114. 10.1016/j.pscychresns.2004.04.002 15313517

[42] Whitehurst TS, Osugo M, Townsend L, et al. Proton magnetic resonance spectroscopy of N-acetyl aspartate in chronic schizophrenia, first episode of psychosis and high-risk of psychosis: A systematic review and meta-analysis. *Neurosci Biobehav Rev*. 2020;119:255-267. 10.1016/j.neubiorev.2020.10.001 33068555

[43] Minichino A, Davies C, Karpenko O, et al. Preventing psychosis in people at clinical high risk: an updated meta-analysis by the world psychiatric association preventive psychiatry section. *Mol Psychiatry*. 2025;30:2773-2782. 10.1038/s41380-025-02902-8 39953286 PMC12092282

[44] Joyce EM, Roiser JP. Cognitive heterogeneity in schizophrenia. *Curr Opin Psychiatry*. 2007;20:268-272. 10.1097/YCO.0b013e3280ba4975 17415081 PMC2597188

[45] Haatveit B, Westlye LT, Vaskinn A, et al. Intra- and inter-individual cognitive variability in schizophrenia and bipolar spectrum disorder: an investigation across multiple cognitive domains. *Schizophrenia*. 2023;9:89. 10.1038/s41537-023-00414-4 38110366 PMC10728206

[46] Palaniyappan L, Liddle PF. Does the salience network play a cardinal role in psychosis? An emerging hypothesis of insular dysfunction. *J Psychiatry Neurosci*. 2012;37:17-27. 10.1503/jpn.100176 21693094 PMC3244495

[47] Smucny J, Dienel SJ, Lewis DA, Carter CS. Mechanisms underlying dorsolateral prefrontal cortex contributions to cognitive dysfunction in schizophrenia. *Neuropsychopharmacology*. 2022;47:292-308. 10.1038/s41386-021-01089-0 34285373 PMC8617156

[48] Bird CM, Burgess N. The hippocampus and memory: insights from spatial processing. *Nat Rev Neurosci*. 2008;9:182-194. 10.1038/nrn2335 18270514

[49] Kunii Y, Zhang W, Xu Q, et al. CHRNA7 and CHRFAM7A mRNAs: co-localized and their expression levels altered in the postmortem dorsolateral prefrontal cortex in major psychiatric disorders. *Am J Psychiatry*. 2015;172:1122-1130. 10.1176/appi.ajp.2015.14080978 26206074

[50] Smucny J, Carter CS, Maddock RJ. Greater choline-containing compounds and Myo-inositol in treatment-resistant versus responsive schizophrenia: a 1H-magnetic resonance spectroscopy meta-analysis. *Biol Psychiatry Cogn Neurosci Neuroimaging*. 2024;9:137-145. 10.1016/j.bpsc.2023.10.008 37925074 PMC11192527

[51] Morrison PD, Jauhar S, Young AH. The mechanism of action of clozapine. *J Psychopharmacol (Oxf)*. 2025;39:297-300. 10.1177/02698811251319458 PMC11967075 39945414

[52] Davies MA, Compton-Toth BA, Hufeisen SJ, Meltzer HY, Roth BL. The highly efficacious actions of N-desmethylclozapine at muscarinic receptors are unique and not a common property of either typical or atypical antipsychotic drugs: Is M1 agonism a pre-requisite for mimicking clozapine’s actions? *Psychopharmacology*. 2005;178:451-460. 10.1007/s00213-004-2017-1 15765260

[53] Jørgensen KN, Nesvåg R, Gunleiksrud S, Raballo A, Jönsson EG, Agartz I. First- and second-generation antipsychotic drug treatment and subcortical brain morphology in schizophrenia. *Eur Arch Psychiatry Clin Neurosci*. 2016;266:451-460. 10.1007/s00406-015-0650-9 26547434

[54] Hu X, Zhou H, Zhang D, et al. Clozapine protects dopaminergic neurons from inflammation-induced damage by inhibiting microglial overactivation. *J Neuroimmune Pharmacol Off J Soc NeuroImmune Pharmacol*. 2012;7:187-201. 10.1007/s11481-011-9309-0 PMC3633602 21870076

[55] Joshi YB, Thomas ML, Braff DL, et al. Anticholinergic medication burden–associated cognitive impairment in schizophrenia. *Am J Psychiatry*. 2021;178:838-847. 10.1176/appi.ajp.2020.20081212 33985348 PMC8440496

[56] Krystal JH, Kane JM, Correll CU, et al. Emraclidine, a novel positive allosteric modulator of cholinergic M4 receptors, for the treatment of schizophrenia: a two-part, randomised, double-blind, placebo-controlled, phase 1b trial. *Lancet*. 2022;400:2210-2220. 10.1016/S0140-6736(22)01990-0 36528376

[57] Hutchinson M, Fazzini E. Cholinesterase inhibition in Parkinson’s disease. *J Neurol Neurosurg Psychiatry*. 1996;61:324-325. 10.1136/jnnp.61.3.324-a PMC486563 8795611

[58] Tan ECK, Johnell K, Bell JS, et al. Do acetylcholinesterase inhibitors prevent or delay psychotropic prescribing in people with dementia? Analyses of the Swedish dementia registry. *Am J Geriatr Psychiatry Off J Am Assoc Geriatr Psychiatry*. 2020;28:108-117. 10.1016/j.jagp.2019.06.008 31331724

[59] Carruthers SP, Gurvich CT, Rossell SL. The muscarinic system, cognition and schizophrenia. *Neurosci Biobehav Rev*. 2015;55:393-402. 10.1016/j.neubiorev.2015.05.011 26003527

[60] McCutcheon RA, Keefe RSE, McGuire PM, Marquand A. Deconstructing cognitive impairment in psychosis with a machine learning approach. *JAMA Psychiatry*. 2025;82:57-65. 10.1001/jamapsychiatry.2024.3062 39382875 PMC11465119

