# Supplementary material for: Variability and Magnitude of Choline Levels Across the Psychosis Spectrum: A Meta-analysis
Source: Schizophr Bull. 2026 May 11;52(3):sbag069. doi: 10.1093/schbul/sbag069 (PMC13158245; doi:10.1093/schbul/sbag069)
Supplement: sbag069_Supplementary_materials [file sbag069_supplementary_materials.docx]

**Variability and magnitude of choline dysfunction across the psychosis spectrum: A meta-analysis (Supplementary materials)**

**Appendix - Search terms**

(“schiz*” OR “psychosis” OR “prodrom*” OR “at risk mental state” OR “high risk mental state” OR “ultra high risk” OR “clinical high risk”) AND (“Proton magnetic resonance spectroscopy” OR “proton magnetic resonance spect*” OR “H-MRS” OR “MRS”) AND (“choline” OR “glycerophosphocholine” OR “phosphocholine” OR “cho” OR "metab*")

| **Section and Topic** | **Item #** | **Checklist item** | **Location where item is reported** |
| --- | --- | --- | --- |
| **TITLE** | | |  |
| Title | 1 | Identify the report as a systematic review. | Title page |
| **ABSTRACT** | | |  |
| Abstract | 2 | See the PRISMA 2020 for Abstracts checklist. | Abstract |
| **INTRODUCTION** | | |  |
| Rationale | 3 | Describe the rationale for the review in the context of existing knowledge. | Introduction (page 3) |
| Objectives | 4 | Provide an explicit statement of the objective(s) or question(s) the review addresses. | Introduction (page 4) |
| **METHODS** | | |  |
| Eligibility criteria | 5 | Specify the inclusion and exclusion criteria for the review and how studies were grouped for the syntheses. | Methods (page 6) |
| Information sources | 6 | Specify all databases, registers, websites, organisations, reference lists and other sources searched or consulted to identify studies. Specify the date when each source was last searched or consulted. | Methods (page 6) |
| Search strategy | 7 | Present the full search strategies for all databases, registers and websites, including any filters and limits used. | supplementary appendix |
| Selection process | 8 | Specify the methods used to decide whether a study met the inclusion criteria of the review, including how many reviewers screened each record and each report retrieved, whether they worked independently, and if applicable, details of automation tools used in the process. | Methods (page 6) |
| Data collection process | 9 | Specify the methods used to collect data from reports, including how many reviewers collected data from each report, whether they worked independently, any processes for obtaining or confirming data from study investigators, and if applicable, details of automation tools used in the process. | Methods (page 6) |
| Data items | 10a | List and define all outcomes for which data were sought. Specify whether all results that were compatible with each outcome domain in each study were sought (e.g. for all measures, time points, analyses), and if not, the methods used to decide which results to collect. | Methods (page 6 and 7) |
|  | 10b | List and define all other variables for which data were sought (e.g. participant and intervention characteristics, funding sources). Describe any assumptions made about any missing or unclear information. | Methods (page 6 and 7) |
| Study risk of bias assessment | 11 | Specify the methods used to assess risk of bias in the included studies, including details of the tool(s) used, how many reviewers assessed each study and whether they worked independently, and if applicable, details of automation tools used in the process. | Methods (page 6) |
| Effect measures | 12 | Specify for each outcome the effect measure(s) (e.g. risk ratio, mean difference) used in the synthesis or presentation of results. | Methods (page 6 and 7) |
| Synthesis methods | 13a | Describe the processes used to decide which studies were eligible for each synthesis (e.g. tabulating the study intervention characteristics and comparing against the planned groups for each synthesis (item #5)). | Methods (page 6 and 7) |
|  | 13b | Describe any methods required to prepare the data for presentation or synthesis, such as handling of missing summary statistics, or data conversions. | Methods (page 6 and 7) |
|  | 13c | Describe any methods used to tabulate or visually display results of individual studies and syntheses. | Methods (page 6 and 7) |
|  | 13d | Describe any methods used to synthesize results and provide a rationale for the choice(s). If meta-analysis was performed, describe the model(s), method(s) to identify the presence and extent of statistical heterogeneity, and software package(s) used. | Methods (page 6 and 7) |
|  | 13e | Describe any methods used to explore possible causes of heterogeneity among study results (e.g. subgroup analysis, meta-regression). | Methods (page 7 and 8) |
|  | 13f | Describe any sensitivity analyses conducted to assess robustness of the synthesized results. | Methods (page 7 and 8) |
| Reporting bias assessment | 14 | Describe any methods used to assess risk of bias due to missing results in a synthesis (arising from reporting biases). | Methods (page 6) |
| Certainty assessment | 15 | Describe any methods used to assess certainty (or confidence) in the body of evidence for an outcome. | N/A |
| **RESULTS** | | |  |
| Study selection | 16a | Describe the results of the search and selection process, from the number of records identified in the search to the number of studies included in the review, ideally using a flow diagram. | Results page 9 and figure 1 |
|  | 16b | Cite studies that might appear to meet the inclusion criteria, but which were excluded, and explain why they were excluded. | N/A |
| Study characteristics | 17 | Cite each included study and present its characteristics. | Supplementary table 2 and 3 |
| Risk of bias in studies | 18 | Present assessments of risk of bias for each included study. | Supplementary table 2 and 3 |
| Results of individual studies | 19 | For all outcomes, present, for each study: (a) summary statistics for each group (where appropriate) and (b) an effect estimate and its precision (e.g. confidence/credible interval), ideally using structured tables or plots. | Supplementary table 2 and 3  Figures 2 and 3  Supplementary figures 2A-P, 3A-F, 4A-P, 5A-C, supplementary tables 4-13, tables 1 and 2 |
| Results of syntheses | 20a | For each synthesis, briefly summarise the characteristics and risk of bias among contributing studies. | Supplementary table 2  Supplementary table 37 |
|  | 20b | Present results of all statistical syntheses conducted. If meta-analysis was done, present for each the summary estimate and its precision (e.g. confidence/credible interval) and measures of statistical heterogeneity. If comparing groups, describe the direction of the effect. | Figures 2 and 3  Supplementary figures 2A-P, 3A-F, 4A-P, 5A-C, supplementary tables 4-13, tables 1 and 2 |
|  | 20c | Present results of all investigations of possible causes of heterogeneity among study results. | Supplementary figures 16-23  Supplementary tables 14-20 |
|  | 20d | Present results of all sensitivity analyses conducted to assess the robustness of the synthesized results. | Supplementary figure 1  supplementary table 21-36 |
| Reporting biases | 21 | Present assessments of risk of bias due to missing results (arising from reporting biases) for each synthesis assessed. | Supplementary figures 6-15 |
| Certainty of evidence | 22 | Present assessments of certainty (or confidence) in the body of evidence for each outcome assessed. | N/A |
| **DISCUSSION** | | |  |
| Discussion | 23a | Provide a general interpretation of the results in the context of other evidence. | Discussion (pages 13-16) |
|  | 23b | Discuss any limitations of the evidence included in the review. | Discussion (pages 13-16) |
|  | 23c | Discuss any limitations of the review processes used. | Discussion (pages 13-16) |
|  | 23d | Discuss implications of the results for practice, policy, and future research. | Discussion (pages 13-16) |
| **OTHER INFORMATION** | | |  |
| Registration and protocol | 24a | Provide registration information for the review, including register name and registration number, or state that the review was not registered. | Methods (page 6) |
|  | 24b | Indicate where the review protocol can be accessed, or state that a protocol was not prepared. | Methods (page 6) |
|  | 24c | Describe and explain any amendments to information provided at registration or in the protocol. | N/A |
| Support | 25 | Describe sources of financial or non-financial support for the review, and the role of the funders or sponsors in the review. | Declaration of interests and acknowledgements (page 17) |
| Competing interests | 26 | Declare any competing interests of review authors. | Declaration of interests and acknowledgements (page 17) |
| Availability of data, code and other materials | 27 | Report which of the following are publicly available and where they can be found: template data collection forms; data extracted from included studies; data used for all analyses; analytic code; any other materials used in the review. | Data availability statement (page 17) |

*From:*  Page MJ, McKenzie JE, Bossuyt PM, Boutron I, Hoffmann TC, Mulrow CD, et al. The PRISMA 2020 statement: an updated guideline for reporting systematic reviews. BMJ 2021;372:n71. doi: 10.1136/bmj.n71. This work is licensed under CC BY 4.0. To view a copy of this license, visit <https://creativecommons.org/licenses/by/4.0/>

**Supplementary table 1 – PRISMA checklist**

| **Study** | **Diagnostic group** | **Definition of cohort** | **Location of region of interest** | **Number of cases** | **Number of controls** | **Male (%) – cases** | **Male (%) - controls** | **Age mean (SD) – cases** | **Age mean (SD) – controls** | **Antipsychotic free (%) – cases** | **Antipsychotic naive (%) – cases** |
| --- | --- | --- | --- | --- | --- | --- | --- | --- | --- | --- | --- |
| Hardy 2011^1^ | Schizophrenia | Clinical diagnosis of schizophrenia or schizoaffective disorder | Combined findings from rostral and caudal ACC | 22 | 11 | 59 | 64 | 39.36 (10.57) | 35.5 (10.7) | 4.55 | NR |
| Modinos 2011^2^ | UHR/CHR | High schizotypal characteristics | 133mm above the anterior section of the genu of the corpus callosum at 90 degrees to the anterior commissure | 22 | 21 | 50 | 57 | 27.36 (7.61) | 27 (5.64) | NR | NR |
| Reid 2019^3^ | Schizophrenia | Clinical diagnosis of schizophrenia or schizoaffective disorder | Dorsal ACC | 21 | 21 | 76 | 76 | 23.2 (4.4) | 23.5 (4.5) | 4.76 | 0 |
| Rowland 2016^4^ | Schizophrenia | SCID for DSM-IV-TR of schizophrenia or schizoaffective disorder^5^ | The voxel was prescribed on the midsagittal slice and positioned parallel to the genu of the corpus callosum and scal, and with the anterior boundary of the voxel placed in line with the genu of the corpus callosum | 27 | 29 | 63 | 48 | 34.4 (13.1) | 29.7 (9.4) | 18.52 | NR |
| Smucny 2022^6^ | Schizophrenia | Clinically defined recent onset schizophrenia spectrum disorders | dlPFC: left middle frontal gyrus over Brodmann areas 9 and 46 angled to be parallel to the brain surface  Occipital lobe: centred on the midline of the calcarine fissures | 40 | 47 | 68 | 68 | 20.55 (3.3) | 21.17 (3.35) | 10 | NR |
| Liu 2015^7^ | FEP | First episode psychosis as per ICD-10 criteria | Bilateral body of the hippocampus, which were identified in normal-appearing hippocampal tissue at the midbrain level | 40 | 17 | 45 | 41 | 24.7 (6.48) | 26.94 (6.11) | 100 | 100 |
| Lutkenhoff 2010^8^ | Schizophrenia | Schizophrenic spectrum disorders as per DSM-IV^5^ | Hippocampus: hippocampus, Para hippocampal gyrus, fusiform gyrus, and collateral sulcus  mPFC: parts of the cingulate sulcus, cingulate gyrus, frontal pole and superior frontal gyrus  FWM: middle frontal gyrus and supperior frontal gyrus focussed on white matter | 9 | 21 | 56 | 57 | 48.8 (11.5) | 55.7 (3.8) | NR | NR |
| MacKinley 2022^9^ | FEP | Clinical diagnosis of first episode psychosis | Bilateral dorsal ACC | 57 | 30 | 84 | 63 | 22.75 (4.28) | 21.57 (3.45) | 47.4 | 47.4 |
| Marsman 2014^10^ | Schizophrenia | Clinical diagnosis of schizophrenia | Medial prefrontal and medial parietal occipital | 14 | 18 | 76 | 70 | 27.6 (6.1) | 27.7 (5.3) | NR | NR |
| Martinez-Granados 2008^11^ | Schizophrenia | DSM-IV criteria for schizophrenia^5^ | Bilateral thalamus | 49 | 37 | 100 | 100 | 40 (11) | 33 (11) | 0 | 0 |
| Merritt 2019^12^ | FEP | First episode of psychosis within the past two years as defined by ICD-10 or DSM-IV criteria | ACC: 16mm superior to the anterior portion of the genu of the corpus callosum on the midline sagittal localiser, avoiding the corpus callosum  Left thalamus: positioned from the axial image, using the coronal and sagittal localisers to minimise cerebrospinal fluid (CSF) content in the voxel. | 23 | 15 | 74 | 80 | 25.5 (5.1) | 24.5 (4.5) | 17.39 | 17.39 |
| Meyer 2016^13^ | Schizophrenia | Schizophrenia or schizoaffective disorder diagnosed on the basis of diagnostic interview for genetic studies^14^ | Hippocampus located as per MR imaging (Atlas of human white matter^15^) | 19 | 11 | 58 | 45 | 40.6 (10.1) | 33.7 (10.1) | 5.26 | NR |
| Miyaoka 2005^16^ | Schizophrenia | Schizophrenia as per DSM-IV criteria | Left hippocampus.  Left basal ganglia.  Cerebellar vermis | 30 | 15 | 50 | 53 | 33.25 (10.69) | 41.7 (14.7) | NR | NR |
| Molina 2005^17^ | Schizophrenia | Schizophrenia as per DSM-IV criteria | Left dorsolateral prefrontal cortex | 28 | 15 | 59 | 60 | 30.3 (8.2) | 28.3 (7.7) | NR | NR |
| Molina 2007^18^ | Schizophrenia | Schizophrenia diagnosed via SCID for DSM-IV criteria^5^ | Left dorsolateral prefrontal cortex | 11 | 10 | 100 | NR | 36.7 (5.8) | 27.2 (4.9) | 0 | 0 |
| Moore 2002^19^ | Schizophrenia | Schizophrenia diagnosed via SCID for DSM-IV criteria^5^ | Right and left medial temporal lobes | 20 | 20 | 70 | 75 | 33.8 (7.1) | 28 (8.3) | 0 | 0 |
| Natsubori 2014^20^ | UHR/CHR | UHR according to SIPS or BIPS criteria^21^ | in the mid-sagittal T2 slice, a  VOI  was placed closest to the most  anterior part of genu of the corpus callosum with the  center of the posterior plane of the VOI. This VOI  contained predominantly the GM of mPFC, including  primarily the anterior cingulate and paracingulate gyri  bilaterally | 24 | 26 | 50 | 50 | 21.7 (3.8) | 22.3 (3.2) | 58.33 | 58.33 |
| Natsubori 2014^20^ | FEP | DSM-IV criteria and less than 16 cumulative weeks of antipsychotics | As above | 19 | 19 | 74 | 74 | 25.4 (6.3) | 26.3 (1.5) | 5.26 | 5.26 |
| Natsubori 2014^20^ | Schizophrenia | DSM-IV schizophrenia as per SCID interview | As above | 25 | 28 | 60 | 61 | 32.7 (8.6) | 32.8 (4.3) | 0 | 0 |
| O'Neill 2004^22^ | Schizophrenia | DSM-IV schizophrenia | Frontal cortex  Superior anterior cingulate  Parietal cortex  Occipital cortex  Striatum  Thalamus  Frontal white matter | 11 | 20 | 64 | 50 | 12.3 (3.8) | 11.7 (2.9) | 18.18 | 18.18 |
| Ohara 2000^23^ | Schizophrenia | ICD 10 criteria for schizophrenia | Striatum | 9 | 9 | 90 | 90 | 27.1 (2.73) | 29.1 (4.56) | 30 | 30 |
| Ohrmann 2008^24^ | Schizophrenia | DSM-IV criteria for schizophrenia | ACC: bilateral rostral ACC  dlPFC: positioned in the left prefrontal cortex as per Rajkowska and Goldman-Rakic^25^ | 43 | 37 | 76 | 53 | 27.9 (8.2) | 27.2 (5.9) | 0 | 0 |
| Ohrmann 2007^26^ | FEP | First episode neuroleptic naive schizophrenia patients as per Structured clinical interview for DSM-IV^5^ | ACC: Rostral ACC  DLPFC: Brodmann area 9,10, 56 | 15 | 20 | 67 | 65 | 27 (6.9) | 28.1 (6.5) | 100 | 100 |
| van Elst 2005^27^ | Schizophrenia | Schizophrenia patients as per Structured clinical interview for DSM-IV^5^ | Left dlPFC  Left hippocampus | 21 | 32 | 62 | 72 | 28.5 (6.42) | 28.2 (5.66) | 0 | 0 |
| Omori 2000^28^ | Schizophrenia | DSM-IV criteria for schizophrenia | Left thalamus  Left frontal lobe | 20 | 16 | 60 | 63 | 30.2 (5.8) | 28.7 (4.1) | 20 | NR |
| Ongür 2010^29^ | Schizophrenia | Schizophrenia patients as per Structured clinical interview for DSM-IV^5^ | Anterior cingulate cortex  Parietal-occipital cortex | 21 | 19 | 67 | 63 | 39 (10.8) | 36.3 (9.8) | 4.76 | NR |
| Ota 2012^30^ | Schizophrenia | Schizophrenia patients as per Structured clinical interview for DSM-IV^5^ | Frontal white matter  Parietal lobe | 46 | 27 | 50 | 48 | 43.23 (13.54) | 42.8 (15.1) | 0 | 0 |
| Ota 2015^31^ | Schizophrenia | Schizophrenia patients as per Structured clinical interview for DSM-IV^5^ | Left frontal white matter  Left parietal lobe | 17 | 22 | 53 | 50 | 40.6 (12.3) | 41.9 (14.9) | NR | NR |
| Ozcelik 2020^32^ | Schizophrenia | Schizophrenia patients as per Structured clinical interview for DSM-IV^5^ | Hippocampus | 16 | 8 | 63 | 63 | 10.94 (3.66) | 11.38 (3.2) | 12.5 | 0 |
| Pae 2004^33^ | Schizophrenia | Clinical diagnosis of schizophrenia | Frontal white matter | 24 | 20 | 46 | 50 | 31 (6.1) | 29 (3.4) | 100 | 41.67 |
| Plitman 2018^34^ | Schizophrenia | DSM-IV diagnosis of schizophrenia confirmed by MINI interview^35^ | Striatum: placed on an oblique axial  image obtained parallel to the anterior commissure-posterior commis-  sure (AC-PC) line; the center of the voxel was 14 mm superior to the AC-  PC line. | 12 | 9 | 58 | 73 | 45 (12.09) | 40.73 (12.95) | 0 | 0 |
| Plitman 2016^36^ | FEP | DSM-IV for schizophrenia, less than two years of psychotic symptoms and antipsychotic naive | Striatum: The lower end  of the dorsal-caudate voxel (associative striatum) was  located 3mm dorsal to the anterior commissure to  include maximum gray matter (GM) and with a dorsal  extension (thickness) of 2 cm. | 60 | 60 | 62 | 63 | 24.67 (7.68) | 23.03 (4.87) | 100 | 100 |
| Reid 2013^37^ | Schizophrenia | Clinical diagnosis of schizophrenia or schizoaffective disorder | Substantia nigra | 35 | 22 | 74 | 59 | 37.9 (12) | 37.9 (12.4) | 0 | 0 |
| Reid 2010^38^ | Schizophrenia | Clinical diagnosis of schizophrenia or schizoaffective disorder | Bilateral dorsal ACC | 26 | 23 | 69 | 65 | 40.4 (13.1) | 37.2 (12.4) | 0 | 0 |
| Reid 2016^39^ | Schizophrenia | Clinical diagnosis of schizophrenia or schizoaffective disorder | Bilateral dosrsal ACC  Left hippocampus | 23 | 18 | 69 | 70 | 33.8 (11) | 37.1 (11.1) | 48.27 | 20.69 |
| Rowland 2013^40^ | Schizophrenia | Diagnosis of schizophrenia as per Structured clinical interview for DSM-IV-TR^5^ | The ACC voxel was prescribed on the midsagit-  tal slice and positioned parallel to the genu of the corpus  callosum and scalp with the midline of the voxel placed  directly above the most anterior tip of the genu of the corpus callosum.  CSO: prescribed on the axial  slice superior to the corpus callosum and positioned parallel and midline to the body of the corpus callosum. | 21 | 20 | 76 | 60 | 40.15 (11.97) | 41.4 (9.72) | 0 | 0 |
| Rowland 2009^41^ | Schizophrenia | Diagnosis of schizophrenia as per Structured clinical interview for DSM-IV^5^ | Left dlPFC  Left inferior parietal lobe | 20 | 11 | 80 | 73 | 38.5 (8.25) | 43 (6) | 5 | 0 |
| Sarramea-Crespo 2008^42^ | Schizophrenia | Diagnosis of schizophrenia as per Structured clinical interview for DSM-IV^5^ | ACC | 14 | 15 | 21 | 27 | 34 (9) | 34 (8) | 14.3 | NR |
| Seese 2011^43^ | Schizophrenia | Schizophrenia as per (K-SADS-PL)^44^ | Inferior frontal gyrus  Middle frontal gyrus  Superior temporal gyrus | 28 | 34 | 54 | 44 | 14.1 (3) | 11.5 (2.9) | 17.86 | NR |
| Shakory 2018^45^ | UHR/CHR | As per criteria of prodromal syndromes^46^ | The voxel was oriented along the long  axis of the head of the left hippocampus | 25 | 31 | 48 | 42 | 21 (2.1) | 22.2 (3.1) | 76 | NR |
| Shakory 2018^45^ | FEP | SCID for DSM-IV Schizophrenia and within 36 months of presentation^5^ | As above | 16 | 31 | 94 | 42 | 24.5 (5.8) | 22.2 (3.1) | 81.25 | NR |
| Sharma 1992^47^ | Schizophrenia | Clinical diagnosis of schizophrenia | Occipital cortex  Basal ganglia | 4 | 9 | NR | 67 | NR (NR) | 31 (4.8) | 0 | 0 |
| Shiori 1996^48^ | Schizophrenia | Diagnoses if schizophrenia as per DSM-III-R criteria | Left basal ganglia | 21 | 21 | 57 | NR | 29.5 (10.2) | 29.5 (9.6) | 0 | 0 |
| Shirayama 2010^49^ | Schizophrenia | SCID for DSM-IV Schizophrenia^5^ | mPFC: placed on the corpus callosum  and centered on the intrahemispheric fissure, not containing the  orbitofrontal cortex. | 19 | 18 | 78 | 63 | 30.5 (5.6) | 31.4 (8.4) | 5.26 | 0 |
| Sigmundsson 2003^50^ | Schizophrenia | DSM-IV Schizophrenia | Bilateral dlPFC | 25 | 26 | 96 | 85 | 34.9 (8) | 31.8 (6.7) | 0 | 0 |
| Singh 2018^51^ | Schizophrenia | Schizophrenia as per the Diagnostic Interview for Genetic  Studies (DIGS)^14^ | Right hippocampus | 28 | 28 | 43 | 50 | 33.89 (9.34) | 31.44 (7.36) | 0 | 0 |
| Sivaraman 2017^52^ | FEP | First episode psychosis as per the Diagnostic Interview for Genetic  Studies (DIGS) and consensus of two board certified psychiatrists^14^ | Left associative striatum centered on internal capsule to include  the caudate and putamen and maximize the gray matter, without  containing any portion of the left lateral ventricle medially, anteriorly  and superiorly | 14 | 18 | 71 | 61 | 22.86 (5.78) | 23.22 (5.39) | 100 | 100 |
| Smesny 2022^53^ | UHR/CHR | CAARMS interview according to Personal Assessment and  Crisis Evaluation (PACE) criteria^54^: | mPFC  ACC  Thalamus | 69 | 61 | 59 | 54 | 26.2 (6.2) | 25.2 (4.8) | 68 | NR |
| Stanley 1995^55^ | FEP | First episode psychosis unmedicated as per SCID for DSM-III-TR | Left dlPFC | 13 | 6 | 85 | 100 | 26 (7) | 32 (11) | 100 | 100 |
| Stanley 1995^55^ | Schizophrenia | Chronic medicated patients with schizophrenia as per SCID for DSM-III-TR | Left dlPFC | 12 | 6 | 92 | 100 | 41 (5) | 32 (11) | 0 | 0 |
| Stone 2009^56^ | UHR/CHR | Patients meeting Personal Assessment  Crisis Evaluation criteria for the ARMS^57^: | ACC: ROI placed 13mm above the anterior section of the genu of the corpus callosum at 90 degrees to the AC-PC line  Left hippocampus: prescribed from a coronal SPGR image  Left thalamus: the point in the coronal slices where the thalamus was widest | 27 | 27 | 48 | 52 | 25 (5) | 25 (4) | 92.60 | 81.50 |
| Szulc 2011^58^ | Schizophrenia | Schizophrenia as per DSM-IV | dlPFC: cortex of the superior and medial frontal gyrus  Temporabl: infero-lateral region of the temporal lobe  Thalamus: placed on the slice where the thalamus is most promiment | 42 | 26 | 71 | 62 | 32.2 (6) | 29.9 (5.3) | 0 | 0 |
| Tanaka 2006^59^ | Schizophrenia | DSM-IV for schizophrenia | dlPFC: Brodmann area 9 | 14 | 13 | 71 | 77 | 29.4 (4.1) | 29.5 (4.1) | 0 | 0 |
| Tarumi 2020^60^ | Schizophrenia | Schizophrenia as per DSM-V | ACC: the tip of the voxel was placed on top of the most anterior par of genu paralleling to the cingulate cortex  Striatum: positioned on an oblique axial image acquired parallel to the anterior-commissure-posterior commissure line and it was centre was 14mm superior to the AC-PC line | 26 | 28 | 45 | 45 | 42.4 (13) | 43.7 (11.7) | 0 | 0 |
| Tarumi 2020^60^ | TRS | TRS defined as TRRIP working group consensus criteria ^61^ | As above | 25 | 28 | 43 | 45 | 43.9 (11.6) | 43.7 (11.7) | 0 | 0 |
| Tayoshi 2009^62^ | Schizophrenia | DSM-IV-TR for schizophrenia | ACC  Left basal ganglia | 30 | 25 | 52 | 47 | 34.9 (10.7) | 33.8 (9.5) | 0 | 0 |
| Tibbo 2000^63^ | Schizophrenia | DSM-IV for schizophrenia | the entire  cerebellar vermis; however, the size of the voxel  required to optimize the signal-to-noise ratio resulted  inclusion of small portions of the cerebellar hemi-  spheres as well. | 12 | 12 | 100 | 100 | 34.1 | 27.9 | 0 | 0 |
| Tibbo 2013^64^ | FEP | Patients with FEP were  only eligible for inclusion if they had at least two active  DSM-IV-TR symptoms of psychosis of more than 1  month but less than 12 months duration, as assessed by  a psychiatrist. | mPFC | 33 | 41 | 70 | 72 | 21.6 (3.4) | 21.9 (3.1) | 100 | 24.2 |
| Uhl 2011^65^ | UHR/CHR | UHR – any of (1) BLIPs (2) attenuated psychotic symptoms on SIPS/SOPS  (3) as per BSABS-P (4) genetic risk combined with worsening of social functioning | ACC: midsagittal superior to anterior border of the corpus callosum  mPFC: midsagittal anterior to the corpus callosum  Left hippocampus: voxel comprising  the left hippocampal head with the voxel being tilted along the  hippocampal axis. | 26 | 25 | 63 | 52 | 30 (4.5) | 21 (5.2) | 83.3 | NR |
| Uhl 2011^65^ | FEP | First episode patients met DSM-IV criteria for schizophrenia and  had not experienced an episode with psychotic symptoms longer  than one week before the current episode which itself did not last  longer than one year by the time of examination. | As above | 22 | 25 | 71 | 52 | 24 (6.3) | 21 (5.2) | 83.3 | NR |
| Vingerhoets 2019^66^ | Unspecified psychosis | Psychosis spectrum disorders as per the Comprehensive assessment of symptoms and history (CASH)^67^ | ACC  Striatum | 30 | 30 | 70 | 67 | 27.33 (4.66) | 25.4 (5) | 100 | 26.68 |
| Wang 2019^68^ | FEP | Recruited within 24 months of first onset of psychotic symptoms and assess using SCID for DSM-IV^5^ | Bilateral dorsal ACC  Left centrum semiovale  Left orbitofrontal region  Bilateral thalamus | 81 | 91 | 70 | 46 | 22.3 (4.4) | 23.3 (3.9) | NR | 0 |
| Wang 2022^69^ | Schizophrenia | Schziophrenias as per DSM-IV-TR by two  trained senior psychiatrists using Mini-International Neuropsychiatric  Interviews^35^ | mPFC: was placed anterior to the genu corpus  callosum and parallel to the anterior-to-posterior commissure | 114 | 59 | 47 | 42 | 26.25 (5.8) | 27.25 (6.1) | NR | NR |
| Wijtenburg 2021^70^ | Schizophrenia | Schizophrenias as per Structured Clinical Interview for DSM-IV-  TR (SCID)^5^ | ACC, left CSO,  left DLFPC, left hippocampus, and bilateral thalamus | 40 | 38 | 58 | 100 | 34.2 (12.4) | 30.5 (12.5) | 0 | 0 |
| Wijtenburg 2017^71^ | Schizophrenia | Schizophrenias as per Structured Clinical Interview for DSM-IV-  TR (SCID)^5^ | mPFC | 95 | 93 | 63 | 54 | 37.2 (13.2) | 37.4 (14) | 0 | 0 |
| Wood 2003^72^ | UHR/CHR | patients were recruited if they had experienced either brief  self-limiting bursts of acute psychotic symptoms with a  duration of less than 1 week (BLIPS), and/or subthreshold  attenuated psychotic symptoms (Attenuated group), and/or  they had a family history of a psychotic disorder or per-  sonal history of schizotypal personality disorder in con-  junction with a marked deterioration in global functioning  maintained for at least 1 month | Medial temporal lobe: the midpoint  of the medial temporal voxel was positioned 4.5 mm  posterior to the amygdala  dlPFC: midpoint of the middle frontal voxel was positioned 7.5 mm anterior to the  genu of the corpus callosum | 56 | 21 | 57 | 62 | 19.5 (3.5) | 34.1 (10) | 100 | 100 |
| Wood 2003^72^ | FEP | SCID for DSM-IV schizophrenia, during initial treatment period and aged between 15 and 30^5^ |  | 30 | 21 | 64 | 62 | 21.7 (3.2) | 34.1 (10) | 9 | NR |
| Yamasue 2003^73^ | Schizophrenia | Schizophrenia as per DSM-IV (SCID)^5^ | Left putamen | 16 | 15 | 63 | 60 | 30.7 (6.9) | 28.4 (3.4) | 0 | 0 |
| Yamasue 2002^74^ | Schizophrenia | Schizophrenia as per DSM-IV (SCID)^5^ | ACC | 15 | 13 | 75 | 62 | 30.4 (7) | 28.8 (3.6) | NR | NR |
| Yasukawa 2005^75^ | Schizophrenia | Schizophrenia as per DSM-IV (SCID)^5^ | Left ACC  Left insular cortex  Left thalamus | 30 | 20 | 53 | 50 | 32.65 (5.69) | 36.1 (6.8) | 23.3 | NR |
| Zabala 2007^76^ | FEP | First-episode psychosis was assessed using  the Kiddie-Schedule for Affective Disorders and Schizophrenia-Present and Lifetime Version (K-SADS-PL),  following DSM-IV criteria^44^. | DLPFC: The voxel was first placed anterior to the middle  frontal gyrus in axial orientation, and then rotated (yaw,  pitch, and roll) to maximize the gray matter (GM) con-  tent inside the voxel. | 8 | 32 | 88 | 70 | 15.63 (2.13) | 15.42 (1.52) | 0 | 0 |
| Ongür 2008^77^ | Schizophrenia | Schizophrenia as per SCID for DSM-IV^5^ | ACC  Parietal lobe | 17 | 21 | 52 | 59 | 41.8 (9.8) | 34.3 (10) | 0 | 0 |
| Aoyama 2011^78^ | Schizophrenia | Schizophrenia as per SCID for DSM-IV^5^ | ACC  Thalamus | 17 | 17 | 82 | 77 | 25 (7) | 19 (10) | 100 | NR |
| Birur 2020^79^ | FEP | Medication naïve FEP as per clinical assessment and Diagnostic Interview for Genetic Studies^14^ | mPFC | 22 | 21 | 64 | 62 | 22.73 (5.52) | 22.95 (5.01) | 100 | 100 |
| Bluml 1999^80^ | Schizophrenia | Schizophrenia as per DSM-IV criteria | Parietal lobe | 13 | 15 | NR | NR | 34.3 (5.8) | 25.3 (4.7) | 15.4 | NR |
| Bossong 2019^81^ | UHR/CHR | CHR as per the Comprehensive Assessment of the At-Risk Mental State^82^ | Left hippocampus | 86 | 30 | 50 | 47 | 22.4 (3.5) | 24.7 (3.8) | 90 | 84 |
| Bustillo 2011^83^ | Schizophrenia | Schizophrenia as per DSM-IV criteria | Gray matter (multiple locations): broad coverage of bilateral  frontal and parietal regions | 30 | 28 | 80 | 64 | 39.04 (7.74) | 39.75 (7.88) | 0 | 0 |
| Bustillo 2010^84^ | Schizophrenia | Schizophrenia as per DSM-IV criteria | ACC  Left frontal white matter  Left thalamus | 14 | 8 | 86 | 80 | 27.2 (8.9) | 21.28 (1.99) | NR | 14 |
| Da Silva 2019^85^ | UHR/CHR | CHR as per prodromal risk syndrome as assessed by  the Criteria of Prodromal Syndromes^46^, and not meeting DSM-IV Axis 1 disorders | mPFC | 35 | 18 | 54 | 33 | 20.57 (1.63) | 21.28 (1.99) | 86 | 86 |
| Deicken 2001^86^ | Schizophrenia | Schizophrenia as per DSM-IV criteria | Cerebellum | 20 | 15 | 100 | 100 | 34.47 (10.58) | 37.47 (11.62) | 0 | 0 |
| Deicken 1999^87^ | Schizophrenia | Schizophrenia as per DSM-IV criteria | Hippocampus: individual voxels selected that were most inclusive of hippocampal grey matter | 23 | 18 | 80 | 66 | 38.5 (10.7) | 36.2 (7.1) | 0 | 0 |
| Demjaha 2014^88^ | TRS | Defined as DSM-IV criteria for schizophrenia and modified Kane criteria for treatment resistance^89^ | ACC | 6 | 10 | NR | NR | NR | NR | NR | NR |
| Demjaha 2014^88^ | Schizophrenia | DSM-IV criteria for schizophrenias per operational criteria checklist ^90^ | ACC | 14 | 10 | NR | NR | NR | NR | NR | NR |
| Egerton 2018^91^ | FEP | Patients were within the first 2 years  of onset of the first psychotic episode, with previous anti-  psychotic exposure of <15 days in the last year and met criteria for schizophrenia as per DSM-IV criteria via the Mini International Neuropsychiatric  Interview^35^ | ACC  Thalamus | 71 | 60 | 68 | 68 | 24.7 (5.4) | NR | 63 | 63 |
| Gan 2017^92^ | Schizophrenia | Schizophrenia as per DSM-IV criteria | mPFC: anterior to the bilateral ventricle anterior an-  gles and avoided influences of the sulci and cerebrospinal fluid.  Thalamus: front of bilateral ventricle pos-  terior angles. | 66 | 35 | 59 | 59 | 27.7 (11) | 26.7 (5.3) | 100 | NR |
| Mcilwain 2015^93^ | FEP | Schizophrenia as per DSM-IV criteria with early treatment response | Left dlPFC  ACC  Left putamen | 15 | 16 | 80 | 81 | 30.7 (7.2) | 34.1 (7.9) | 0 | 0 |
| Mcilwain 2015^93^ | TRS | DSM-IV schizophrenia requiring clozapine treatment | Left dlPFC  ACC  Left putamen | 16 | 16 | 75 | 81 | 33.7 (8.6) | 34.1 (7.9) | 0 | 0 |
| Kragulijac 2013^94^ | Schizophrenia | Schizophrenia as per the consensus of two clinicians using the Diagnostic Interview for Genetic Studies^14^ | Left hippocampus | 27 | 27 | 74 | 74 | 32.63 (9.28) | 32.85 (9.39) | NR | 41 |
| Larabi 2015^95^ | Schizophrenia | Clinical diagnosis of schizophrenia | Left dlPFC | 88 | 35 | 78 | 68 | 32.95 (10.77) | 26.63 (9.86) | 18 | 0 |
| Lim 1998^96^ | Schizophrenia | Schizophrenia as per DSM-IV criteria | Frontal white matter | 10 | 9 | 100 | 100 | 43.6 (5.9) | 41.6 (7.2) | 0 | 0 |
| Auer 2001^97^ | Schizophrenia | Clinical diagnosis of schizophrenia | Left thalamus: included the medio-dorsal nuclei extending to lateral nuclei and partially surrounding structures.  Parietal lobe: positioned to minimise contributions from CSF and grey matter | 30 | 17 | 63 | 53 | 33.9 | 31.2 | 3 | 0 |
| Aydin 2008^98^ | FEP | Schizophrenia as per DSM-IV criteria: no past diagnosis of nonaffective psychosis; no previous  inpatient care and antipsychotic treatment. | Corpus callosum | 14 | 15 | 71 | 66 | 25 (5.46) | 24.8 (4.53) | 29 | 29 |
| Aydin 2008^98^ | UHR/CHR | Brief, limited intermittent psychotic symptoms (BLIPS);  attenuated symptoms; or trait plus state risk factors  (i.e., genetic risk plus decrease in functioning) | Corpus callosum | 17 | 15 | 82 | 66 | 19.58 (3.24) | 24.8 (4.53) | 100 | 100 |
| Bartolomeo 2019^99^ | Unspecified psychosis | DSM-IV diagnosis of schizophrenia, schizoaffective, schizophreniform or psychosis not otherwise specified, as determined by the Structured Clinical Interview for DSM-IV-TR | Other frontal lobe | 34 | 19 | 71 | 74 | 22 (4.3) | 22.9 (3.6) | NR | NR |
| Bertolino 1998^100^ | Schizophrenia | Schizophrenia as per DSM-IV criteria | Thalamus  Striatum  Hippocampus  Temporal lobe  Other frontal lobe  dlPFC  PCC  ACC  Frontal white matter  Centrum semiovale | 12 | 12 | 75 | 75 | 39.6 (11.8) | 40.1 (7.87) | 100 | 42 |
| Bertolino 1996^101^ | Schizophrenia | Schizophrenia as per DSM-IV criteria | Hippocampus  dlPFC | 10 | 10 | 80 | 80 | 37.4 (8.6) | 33.1 (5.45) | 0 | 0 |
| Blasi 2004^102^ | Unspecified psychosis | First episode affective psychosis as per DSM-IV criteria | Hippocampus  Occipital lobe  Striatum  Thalamus  Temporal lobe  Other frontal lobe  Frontal white matter  PCC  ACC  dlPFC  Centrum semiovale | 17 | 17 | 59 | 59 | 26.8 (7.6) | 25.5 (6.8) | 0 | 0 |
| Block 2000^103^ | Schizophrenia | Schizophrenia as per DSM-IV criteria using the Schedule for Affective Disor-  ders and Schizophrenia-Lifetime Version^104^ | Other frontal lobe: anterior to the precentral sulcus in the left  frontal cortex and subjacent white matter.  Striatum: putamen and parts of globus pallidus and thalamus. | 25 | 19 | 72 | 37 | 35.6 (8.3) | 40.2 (15.3) | NR | NR |
| Brandt 2016^105^ | Schizophrenia | Clinical diagnosis of schizophrenia | Dorsal ACC: positioned just superior to the genu of the corpus callosum | 27 | 27 | 79 | 83 | 37.5 (16.7) | 36.6 (1.6) | 0 | 0 |
| Brooks 1998^106^ | Schizophrenia | Childhood schizophrenia as per Kiddie Schedule of Affective Disorders and Schizo-  phrenia (K-SADS)^44^ | Frontal white matter | 16 | 12 | 56 | 50 | 10.8 (1.6) | 11 (1.6) | 25 | NR |
| Bustillo 2014^107^ | Schizophrenia | Schizophrenia as per DSM-IV-TR (SCID)^5^ | ACC: positioned parallel to and above the corpus callosum, starting from the genu of the corpus callosum and extending 3 cm posteriorly | 84 | 81 | 85 | 67 | 36.7 (13.9) | 35.2 (11.8) | 3.6 | NR |
| Bustillo 2017^108^ | Schizophrenia | Schizophrenia as per DSM-IV-TR (SCID)^5^ | White matter (multiple locations)  Grey matter (multiple locations) | 104 | 97 | 86 | 71 | 36.3 (13.8) | 37.1 (12.3) | 7 | NR |
| Bustillo 2019^109^ | Unspecified psychosis | Bipolar-I with history of psychotic features during manic or mixed  episodes and schizophrenia patients as per DSM-IV-TR (SCID) | White matter (multiple locations)  Grey matter (multiple locations) | 43 | 41 | 60 | 63 | 38.7 (12.9) | 38.8 (13.6) | 14 | NR |
| Bustillo 2001^110^ | Schizophrenia | Schizophrenia as per DSM-IV (SCID)^5^ | Left frontal white matter  Right caudate nuclei | 35 | 17 | 66 | 52 | 41.2 (8.7) | 38.8 (11.6) | 0 | 0 |
| Bustillo 2002a^111^ | Schizophrenia | Schizophrenia as per DSM-IV (SCID)^5^ | Left frontal white matter  Left occipital lobe | 10 | 10 | 80 | 80 | 27.2 (8.1) | 26.8 (5.9) | 60 | 60 |
| Bustillo 2008^112^ | Schizophrenia | Schizophrenia as per DSM-V (SCID)^113^ | Striatum  Frontal white matter  Occipital lobe  Cerebellum | 27 | 19 | 81 | 86 | 24.7 (6.9) | 24.7 (5.3) | 47 | 47 |
| Bustillo 2002b^114^ | Schizophrenia | Schizophrenia as per DSM-IV (SCID)^5^ | Caudate | 13 | 12 | 73 | 91 | 26 (9.2) | 32.5 (8.1) | 0 | 0 |
| Byun 2009^115^ | UHR/CHR | Met the criteria for UHR as per CAARMS checklist for psychosis and at least one of following: attenuated psychotic symptoms, brief, limited intermittent psychotic symptoms (BLIPS), or trait plus state  risk factors^54^ | ACC: perpendicularly to the tip  of the genu of the corpus callosum and centered at the  interhemispheric fissure.  Left dlPFC: 7.5 mm anterior to the genu of the corpus callosum  Left thalamus | 20 | 20 | 55 | 40 | 21.8 (4.11) | 22 (4.11) | NR | 55 |
| Callicott 1998^116^ | Schizophrenia | Schizophrenia as per DSM-IV (SCID)^5^ | Hippocampus  dlPFC  Other frontal lobe  ACC  PCC  Centrum semiovale  Occipital lobe  Frontal white matter  Thalamus  Striatum  Temporal lobe | 47 | 66 | 91 | 64 | 34.2 (8.8) | 32.9 (8.2) | 17 | 0 |
| Cecil 1999^117^ | FEP | Neuroleptic naïve first episode psychosis patients diagnosed via the SCID-P | dlPFC  Temporal lobe | 10 | 10 | NR | NR | 27 (5) | 34.2 (6.2) | 100 | 100 |
| Chang 2007^118^ | Schizophrenia | Schizophrenia as per DSM-IV (SCID)^5^ | Frontal white matter  Temporal lobe  Occipital lobe | 23 | 22 | NR | NR | 66.3 (7.2) | 70 (5.3) | 9 | 0 |
| Chiu 2018^119^ | FEP | First episode psychosis as per DSM-IV (SCID)^5^ | ACC: anterior tip of the genu of the corpus callosum | 19 | 14 | 58 | 64 | 29.11 (6.68) | 27.71 (5.88) | 0 | 0 |
| Crocker 2014^120^ | FEP | Active symptoms for at least one month but less than 12 months and meeting criteria for schizophrenia as per DSM-IV-TR | mPFC | 29 | 45 | 21 | 80 | 21.9 (3.4) | 22.4 (3.3) | 100 | 21 |
| de la Fuente Sandoval 2013^121^ | FEP | First episode psychosis as per DSM-IV (SCID) – symptoms for less than one year and antipsychotic naïve | Right Cerebellar cortex  Right associative striatum | 24 | 18 | 54 | 44 | 26.58 (8.49) | 24.56 (5.07) | 100 | 100 |
| de la Fuente Sandoval 2011^122^ | UHR/CHR | Met Structured Interview for Prodromal  Syndromes (SIPS) criteria^46^ | Right dorsal caudate  Right cerebellum | 18 | 40 | 77 | 70 | 19.56 (3.46) | 21.83 (4.47) | 100 | 100 |
| de la Fuente Sandoval 2018^123^ | FEP | Antipsychotic naïve first episode psychosis as per DSM-IV (SCID)^5^ | mPFC: portions of Brodmann areas 24, 32, and 10 and the  pregenual anterior cingulate cortex)  Dorsal caudate bilateral | 45 | 47 | 71 | 50 | 23 (6.1) | 23 (3.8) | 100 | 100 |
| de la Fuente Sandoval 2015^124^ | UHR/CHR | Met Structured Interview for Prodromal  Syndromes (SIPS) criteria^46^ | mPFC: portions of Brodmann areas 24, 32, and 10 and the  pregenual anterior cingulate cortex)  Dorsal caudate bilateral | 23 | 24 | 65 | 79 | 20.7 (4.1) | 21.4 (3.3) | 100 | 100 |
| Deicken 2000^125^ | Schizophrenia | Schizophrenia as per DSM-IV (SCID)^5^ | Left thalamus | 17 | 10 | 100 | 100 | 35.7 (9.3) | 39.5 (12.8) | 0 | 0 |
| Deicken 1997a^126^ | Schizophrenia | Schizophrenia as per DSM-III-R (SCID)^5^ | Frontal white matter | 24 | 15 | 88 | 73 | 35.7 (12.2) | 36.6 (6.8) | 0 | 0 |
| Deicken 1998^127^ | Schizophrenia | Schizophrenia as per DSM-III-R (SCID)^5^ | Hippocampus | 30 | 18 | 80 | 67 | 38.5 (10.7) | 36.2 (7.1) | 7 | 0 |
| Deicken 1997b^128^ | Schizophrenia | Schizophrenia as per DSM-III-R (SCID)^5^ | ACC | 26 | 16 | 85 | 75 | 37 (10.7) | 35.8 (7.3) | 8 | 0 |
| Delamillieure 2000a^129^ | Schizophrenia | Schizophrenia as per DSM-IV (SCID)^5^ | Thalamus | 27 | 24 | NR | NR | 30 (7) | 30.2 (7.1) | NR | NR |
| Delamillieure 2002^130^ | Schizophrenia | Schizophrenia as per DSM-IV (SCID)^5^ | Hippocampus | 17 | 14 | 82 | 79 | 31.25 (6.09) | 30.14 (6.39) | 29 | 29 |
| Delamillieure 2000b^131^ | Schizophrenia | Schizophrenia as per DSM-IV (SCID)^5^ | mPFC | 22 | 21 | NR | NR | NR | NR | 0 | 0 |
| Ende 2001^132^ | Schizophrenia | Schizophrenia as per DSM-III-R (SCID)^5^ | Thalamus | 15 | 15 | 67 | 53 | 33 (7) | 31.9 (6.7) | 0 | 0 |
| Fannon 2003^133^ | FEP | Schizophrenia as per DSM-IV (SCID) experiencing first episode of psychosis and age 16-40^5^ | Hippocampus  Frontal white matter  Striatum | 12 | 9 | 55 | 72 | 26.1 (5.5) | 25.3 (6.7) | 100 | 100 |
| Fujimoto 1996^134^ | Schizophrenia | Schizophrenia as per DSM-III-R (SCID)^5^ | Striatum | 14 | 12 | 100 | 83 | 39 (5.6) | 38 (7.3) | 0 | 0 |
| Fukuzako 2000^135^ | Schizophrenia | Schizophrenia as per DSM-III-R (SCID)^5^ | Left medial temporal lobe | 64 | 51 | 53 | 49 | 36.5 (7.2) | 35.5 (7.9) | 0 | 0 |
| Fukuzako 1995^136^ | Schizophrenia | Schizophrenia as per DSM-III-R (SCID)^5^ | Frontal white matter  Left medial temporal lobe | 15 | 15 | 27 | 27 | 39.3 (7.6) | 38.8 (7.8) | 0 | 0 |
| Galinska 2009^137^ | Schizophrenia | Meeting ICD-10 criteria for schizophrenia^138^ | Left temporal lobe  Frontal white matter  Other frontal lobe (left)  Left thalamus | 30 | 19 | 67 | 68 | 22.5 (3.6) | 22.5 (3.3) | 3 | 3 |
| Galinska-Skok 2018^139^ | Schizophrenia | Meeting ICD-10 and DSM-IV criteria for schizophrenia^5^ | Left temporal lobe: middle and inferior temporal gyri  Frontal white matter | 21 | 20 | 48 | 50 | 37.76 (8.04) | 36.95 (7.41) | 0 | 0 |
| Gallinat 2016^140^ | Schizophrenia | Schizophrenia as per DSM-IV (SCID)^5^ | Left hippocampus  ACC | 29 | 29 | 66 | 66 | 27.6 (6.8) | 30.9 (8.4) | 0 | 0 |
| Gan 2014^141^ | Schizophrenia | Schizophrenia as per DSM-IV (SCID)^5^ | mPFC  Thalamus | 41 | 28 | 63 | 57 | 16.6 (1.2) | 16.8 (1.4) | 100 | 100 |
| Granata 2013^142^ | Schizophrenia | Schizophrenia as per DSM-IV (SCID)^5^ | Left dlPFC  Bilateral thalami  Cerebellar vermis | 22 | 12 | NR | NR | 37.4 | NR | 0 | 0 |
| Hagino 2002^143^ | Schizophrenia | Schizophrenia as per ICD-10^138^ | Bilateral thalami  Other frontal lobe: inferior frontal gyrus corresponding  to the pars triangularis | 13 | 13 | 85 | 85 | 23.7 (5.1) | 20.9 (2.3) | 0 | 0 |
| Hasan 2014^144^ | FEP | First episode of schizophrenia as per DSM-IV (SCID)^5^ | Left hippocampus | 46 | 49 | 70 | 39 | 29.22 (7.49) | 39.56 (12.19) | 4 | 4 |
| He 2018^145^ | UHR/CHR | Met Structured Interview for Prodromal  Syndromes (SIPS) criteria^46^ and any of brief Intermittent  Psychotic Syndrome (BIPS), Attenuated Positive Symptom Syn-  drome (APSS) and Genetic Risk and Deterioration Syndrome  (GRDS). | ACC | 74 | 32 | 51 | 53 | 21.67 (5.75) | 22.19 (3.14) | 100 | 100 |
| He 2012^146^ | FEP | First episode of schizophrenia as per DSM-IV (SCID)^5^ | Bilateral frontal white matter  Bilateral hippocampi | 63 | 63 | 57 | 57 | 23.94 (8.48) | 23.97 (8.38) | 100 | 100 |
| Heimberg 1998^147^ | Schizophrenia | Schizophrenia as per DSM-III-R (SCID)^5^ | Left striatum  Left other frontal lobe  Left temporal lobe  Bilateral thalami | 24 | 26 | 100 |  | 43.3 (9.6) | 40 (10.9) | 39 | 0 |
| Huang 2019^148^ | FEP | First episode of schizophrenia meeting ICD-10 criteria and antipsychotic naïve^138^ | dlPFC | 25 | 30 | 48 | 53 | 19.92 (4.65) | 19.23 (4.41) | 100 | 100 |
| Huang 2017^149^ | FEP | First episode of schizophrenia meeting ICD-10 criteria and antipsychotic naïve^138^ | dlPFC | 58 | 43 | 50 | 37 | 22.66 (7.64) | 23.07 (7.49) | 100 | 100 |
| Iwata 2019^150^ | TRS | DSM-IV schizophrenia and TRS as per TRRIP working group^61^ | Striatum: dorsal caudate - positioned  on an oblique axial image acquired parallel to the  anterior commissure–posterior commissure (AC-PC)  line; the voxel was  and  its center was 14 mm superior to the AC-PC line.  ACC: dorsal - positioned on an oblique axial image acquired parallel to the AC-PC line and an oblique sagittal image  acquired to parallel to head midline. The tip of the  voxel was placed on top of the most anterior part of  genu with paralleling to the cingulate cortex.  dlPFC: in AC-PC  space markers were placed on the most anterior  point of the frontal pole and the tip of the temporal  pole. Using these markers, the posterior and anterior boundaries of the DLPFC were determinted | 22 | 26 | 70 | 73 | 40.5 (11.2) | 40.8 (13.2) | 0 | 0 |
| Iwata 2019^150^ | Schizophrenia | Schizophrenia as per DSM-IV (SCID)^5^ | As above | 21 | 26 | 76 | 73 | 46.3 (12.7) | 40.8 (13.2) | 0 | 0 |
| Jakary 2005^151^ | Schizophrenia | Schizophrenia as per DSM-IV (SCID)^5^ | Thalamus | 22 | 22 | 100 | 100 | 34.5 (9.4) | 36.4 (11.3) | 0 | 0 |
| Jessen 2013^152^ | Schizophrenia | Schizophrenia as per DSM-V^113^ | ACC  Other frontal lobe | 20 | 20 | 70 | 55 | 34.5 (10.2) | 30.7 (9.1) | 0 | 0 |
| Jessen 2006^153^ | Schizophrenia | Schizophrenia as per DSM-IV (SCID)^5^ | Other frontal lobe  ACC  Left temporal lobe | 13 | 31 | 90 | 55 | 33.1 (7.2) | 34.8 (13.5) | 0 | 0 |
| Jessen 2006^153^ | UHR/CHR | Meeting criteria for at risk states as per German Research Network on Schizophrenia ^154^ | As above | 17 | 31 | 47 | 55 | 28.7 (7) | 34.8 (13.5) | 89 | 89 |
| Kegeles 2000^155^ | Schizophrenia | Schizophrenia as per DSM-IV (SCID)^5^ | Anterior hippocampi bilaterally | 10 | 10 | 100 | 100 | 28 (7) | 29 (5) | 70 | 10 |
| Kim 2018^156^ | FEP | First episode psychosis with less than a year of symptoms and no more than a single hospitalisation and meeting DSM-IV criteria for schizophrenia spectrum disorders or bipolar disorder with psychotic features (SCID) | ACC  PCC | 40 | 49 | 85 | 39 | 23.62 (4.73) | 25.14 (5.86) | 35 | NR |
| Kirtas 2016^157^ | FEP | Schizophrenia as per DSM-IV (SCID) with less than one month of antipsychotic treatment ^5^ | dlPFC  Hippocampus | 19 | 30 | 47 | 53 | 29.32 (9.92) | 30.93 (6.58) | 5 | 5 |
| Kirtas 2016^157^ | Schizophrenia | Schizophrenia as per DSM-IV (SCID) with at least three years of symptoms ^5^ | As above | 30 | 30 | 60 | 53 | 34.33 (11.19) | 30.93 (6.58) | 0 | 0 |
| Klar 2010^158^ | Schizophrenia | Fulfilling DSM-IV and ICD-10 criteria for schizophrenia^138^ | Hippocampus | 29 | 44 | 34 | 45 | 27.6 (6.8) | 30.9 (7.9) | 7 | NR |
| Kragulijac 2019^159^ | Schizophrenia | Schizophrenia diagnoses established by review of medical records, the Diag-  nostic Interview for Genetic Studies^14^ | Left hippocampus  ACC | 61 | 31 | 72 | 71 | 27.84 (8.69) | 28.58 (9.81) | 100 | 66 |
| Lebedeva 2014^160^ | Schizophrenia | Fulfilling ICD-10 criteria for schizophrenia^138^ | dlPFC  Corpus callosum: genu | 22 | 28 | 100 | 100 | 21.6 (2.9) | 22.8 (1.9) | 0 | 0 |
| Legind 2019^161^ | Schizophrenia | Fulfilling ICD-10 criteria for schizophrenia spectrum disorders^138^ | Left thalamus  ACC | 50 | 85 | 57 | 52 | 40.6 (9.67) | 40.9 (10.63) | 52 | NR |
| Bartha 1997^162^ | FEP | Never treated patients meeting DM-III-R criteria for schizophrenia^5^ | mPFC: Brodmann  areas 24, 32, and 9 | 10 | 10 | 80 | 80 | 24.4 (5.1) | 26.3 (6.4) | 100 | 100 |
| Basoglu 2006^163^ | Schizophrenia | Meeting criteria for schizophrenia as per DSM-IV with diagnosis for over two years ^5^ | Thalamus  Temporal lobe: placed between  the ascendant ramus and the lateral sulcus in the  temporal cortex | 15 | 10 | 100 | 100 | 40.1 (11.2) | 30.9 (7.2) | NR | NR |
| Basoglu 2006^163^ | FEP | First episode of schizophreniform disorder as per DSM-IV and not previously exposed to antipsychotic medication | As above | 13 | 10 | 100 | 100 | 21.9 (2.5) | 30.9 (7.2) | 100 | 100 |
| Chiappelli 2015^164^ | Schizophrenia | Schizophrenia as per DSM-IV (SCID)^5^ | Frontal white matter | 38 | 36 | 74 | 67 | 39.3 (12.8) | 39.1 (12.9) | 8 | NR |
| Choe 1994^165^ | Schizophrenia | Schizophrenia as per DSM-III-R (SCID)^5^ | mPFC | 23 | 10 | 43 | 50 | NR | NR | 100 | 100 |
| Maier 2000^166^ | Schizophrenia | Clinical diagnosis of schizophrenia | Hippocampus | 26 | 38 | NR | NR | 36.38 (8.54) | 27.87 (9.73) | NR | NR |
| Premkumar 2010^167^ | Schizophrenia | Schizophrenia as per DSM-IV (SCID)^5^ | Dorsal ACC | 30 | 15 | 80 | 87 | 36.4 (8.64) | 35.4 (13.34) | 0 | 0 |
| Stanley 2007^168^ | FEP | First episode psychosis, antipsychotic naïve meeting criteria for schizophrenia as per DSM-IV (SCID)^5^ | Left dlPFC | 18 | 61 | 72 | 64 | 23.4 (6.5) | 24 (6.6) | 100 | 100 |
| Taylor 2017^169^ | Schizophrenia | Schizophrenia as per DSM-IV (SCID)^5^ | Left ACC  Left thalamus | 16 | 18 | 81 | 61 | 22.7 (2.9) | 23.9 (4.6) | 12.5 | 0 |
| Terpstra 2005^170^ | Schizophrenia | Schizophrenia as per DSM-IV (SCID)^5^ | ACC | 13 | 3 | 62 | 33 | 26 (5) | 20 (3) | 0 | 0 |
| Theberge 2007^171^ | FEP | Antipsychotic naive with first episode of psychosis and meeting criteria for schizophrenia as per DSM-IV (SCID)^5^ | Left ACC  Left thalamus | 16 | 16 | 88 | 88 | 25 (8) | 29 (12) | 100 | 100 |
| Tunc-Skarka 2009^172^ | Schizophrenia | Schizophrenia as per DSM-IV (SCID)^5^ | Frontal white matter | 23 | 29 | 65 | 41 | 31.73 (8.77) | 32.45 (9.65) | 52.2 | NR |
| Venkatraman 2006^173^ | Schizophrenia | Clinical diagnosis of schizophrenia | Bilateral ACC  Bilateral hippocampi | 29 | 24 | NR | NR | 23.5 (5) | 23.2 (4) | NR | NR |
| Ueno 2022^174^ | TRS | DSM-IV schizophrenia and TRS as per TRRIP working group^61^ | ACC: an oblique axial image  acquired parallel to the AC-PC line and  an oblique sagittal image acquired parallel to the head midline. The tip of the  voxel was placed on top of the most  anterior part of the genu and parallel the  cingulate cortex. | 47 | 35 | 82 | 69 | 40.1 (13) | 43 (15.2) | 0 | 0 |
| Ueno 2022^174^ | Schizophrenia | Schizophrenia as per DSM-IV (SCID)^5^ | As above | 16 | 35 | 81 | 69 | 46.6 (14) | 43 (15.2) | 0 | 0 |
| Cadenhead 2023^175^ | FEP | First episode of psychosis with less than a year of symptoms, antipsychotic naïve and aged 18-30 and meeting criteria for Schizophrenia as per DSM-IV (SCID) | Caudate: 3 mm dorsal to the anterior commissure | 13 | 9 | 84.6 | 77 | 22.5 (4.6) | 21.3 (3.8) | 100 | 100 |
| Chiappelii 2024^176^ | Schizophrenia | Schizophrenia as per DSM-V (SCID)^113^ | Left ACC | 35 | 32 | 71.4 | 39.4 | 39.6 (15.5) | 44.8 (16.8) | 8.6 | 0 |
| Fan 2024^177^ | CHR/UHR | CHR as per screened  using the Structured Interview for Prodromal Syndrome  and fulfilled 1 of 3 criteria, namely attenuated positive symptom syndrome, brief intermittent psychotic syndrome, or gen-  etic risk and deterioration syndrome.^46^ | Dorsal ACC | 63 | 33 | 55.6 | 58.2 | 19.48 (4.72) | 20.6 (3.49) | NR | NR |
| Fan 2024^177^ | FEP | Schizophrenia as per DSM-V (SCID)^113^ | As above | 96 | 34 | 56.2 | 58.2 | 21.21 (5.6) | 20.6 (3.49) | NR | NR |
| Koster 2024^178^ | FEP | Clinical diagnosis of first episode of psychosis with less than one year of antipsychotic medication use | dorsal ACC:  parallel to the corpus callosum on the sagittal midline, equal to the mid  medial frontal cortex | 59 | 35 | 72.9 | 68.6 | 23.85 (4.47) | 22.9 (4.34) | NR | NR |
| León-Ortiz 2023^179^ | FEP | Clinical diagnosis of first episode of psychosis and antipsychotic naïve | Bilateral mPFC: portions of Brodmann areas 10,  24, 32, and the pregenual anterior cingulate cortex | 26 | 14 | NR | NR | NR | NR | 100 | 100 |
| Wang 2023^180^ | FEP | Clinical diagnosis of first episode psychosis within 24 months of first psychotic manifestation | ACC | 33 | 44 | 71 | 54 | 22.53 (4.33) | 23.67 (3.31) | NR | NR |
| Allam 2024^181^ | FEP | Clinical diagnosis of first episode psychosis meeting DSM-V criteria for schizophrenia using the MINI^182^ | Temporal lobe  Hippocampus  Thalamus  dlPFC  Other frontal lobe  Parietal lobe  ACC | 12 | 20 | NR | 60 | NR | 31.1 (3.8) | 100 | NR |
| Allam 2024^181^ | Schizophrenia | DSM-V criteria for schizophrenia using the MINI^182^ | As above | 18 | 20 | NR | 60 | NR | 31.1 (3.8) | 100 | NR |
| Kubota 2023^183^ | Schizophrenia | Schizophrenia as per DSM-V (SCID)^113^ | Striatum  dlPFC | 24 | 17 | 55.6 | 51.9 | 41.8 (9.5) | 42.3 (8) | 18.5 | NR |
| Stanley 2023^184^ | Schizophrenia | Schizophrenia as per DSM-V (SCID)^113^ | Right dlPFC  Right hippocampus  Voxel locations a mapped with Woodcock et al 2018^185^ | 40 | 39 | 79.5 | 74 | 31.8 (8.4) | 28.6 (6.7) | 0 | 0 |
| Olbrich 2008^186^ | FEP | Schizophrenia as per DSM-IV (SCID)^5^ | Left dlPFC  Left Hippocampus | 8 | 25 | 77 | 31.3 | 28.4 (7.3) | 28.2 (5.8) | NR | NR |

**Supplementary table 2 – Clinical characteristics of included studies.** CHR indicates clinical high risk; UHR, ultra-high risk; TRS, treatment resistant schizophrenia; FEP, first episode psychosis; dlPFC, dorsolateral prefrontal cortex; mPFC, medial prefrontal cortex; ACC, anterior cingulate cortex; PCC, posterior cingulate cortex; CSO, centrum semiovale; FWM, frontal white matter; DSM, diagnostic and statistical manual of mental disorders; ICD, international classification of diseases; SCID, structured clinical interview for DSM disorders; PC, posterior commissure; AC, anterior commissure; TRRIP, treatment resistance and response in psychosis.

| **Study** | **Magnet strength (tesla)** | **Vendor** | **Acquisition sequence** | **FWHM (median)** | **CRLB (median)** | **SNR (median)** | **Metabolite reported** | **CSF Corrected** | **Creatinine Corrected** | **TE (ms)** | **TR (ms)** |
| --- | --- | --- | --- | --- | --- | --- | --- | --- | --- | --- | --- |
| Hardy 2011^1^ | 3 | Siemens | PRESS | 5.3 | NR | NR | Choline | Yes | No | 35 | 1800 |
| Modinos 2011^2^ | 3 | GE | PRESS | 4.72 | 3.09 | 25.73 | Choline | Yes | No | 30 | 3000 |
| Reid 2019^3^ | 7 | Siemens | STEAM | 9 | 3.4 | 31.7 | GPC + PCh | No | No | 5 | 10000 |
| Rowland 2016^4^ | 7 | Philips | STEAM | 9.6 | NR | 46.7 | GPC + PCh | Yes | No | 14 | 3000 |
| Smucny 2022^6^ | 3 | Siemens | PRESS | 4.93 | NR | 39.22 | total choline (phosphocholine + glycerophosphorylcholine) | Yes | Yes | 30 | 1500 |
| Liu 2015^7^ | 3 | GE | PRESS | NR | NR | NR | total choline (phosphocholine + glycerophosphorylcholine) | No | Yes | 35 | 1500 |
| Lutkenhoff 2010^8^ | 3 | GE | PRESS | 9.86 | 9.3 | 5.2 | GPC + PCh | Yes | No | 30 | 3000 |
| MacKinley 2022^9^ | 7 | Siemens | semi-LASER | 7.63 | 1.89 | NR | Choline | Yes | No | 100 | 7500 |
| Marsman 2014^10^ | 7 | Philips | semi-LASER | 9 | 2 | 51.9 | Choline | No | No | 28 | 5000 |
| Martinez-Granados 2008^11^ | 1.5 | Philips | PRESS | NR | NR | NR | Choline | No | Yes | 272 | 2700 |
| Merritt 2019^12^ | 3 | GE | PRESS | 4.93 | 3.3 | 20.04 | Choline | Yes | No | 30 | 3000 |
| Meyer 2016^13^ | 3 | Siemens | PRESS | NR | NR | NR | Choline | No | No | 35 | 1400 |
| Miyaoka 2005^16^ | 1.5 | GE | PRESS | NR | NR | NR | Choline | No | Yes | 30 | 1500 |
| Molina 2005^17^ | 1.5 | Philips | PRESS | NR | NR | NR | Choline | No | Yes | 136 | 1500 |
| Molina 2007^18^ | 1.5 | Philips | PRESS | NR | NR | NR | Choline | No | Yes | 136 | 1500 |
| Moore 2002^19^ | 1.5 | GE | PRESS | NR | NR | 11.7 | Cytosolic choline compounds | No | Yes | 135 | 2000 |
| Natsubori 2014^20^ | 3 | GE | STEAM | 8.25 | 4.4 | 12 | GPC + PCh | Yes | No | 15 | 3000 |
| Natsubori 2014^20^ | 3 | GE | STEAM | 8.38 | 4 | 8.7 | GPC + PCh | Yes | No | 15 | 3000 |
| Natsubori 2014^20^ | 3 | GE | STEAM | 10.96 | 5.3 | NR | GPC + PCh | Yes | No | 15 | 3000 |
| O'Neill 2004^22^ | 1.5 | GE | CHESS | NR | NR | NR | choline compounds | Yes | No | 272 | 2300 |
| Ohara 2000^23^ | 1.5 | GE | CHESS | NR | NR | NR | choline compounds | No | Yes | 272 | 2300 |
| Ohrmann 2008^24^ | 1.5 | Philips | PRESS | NR | NR | NR | choline-containing compounds | No | No | 40 | 1500 |
| Ohrmann 2007^26^ | 1.5 | Siemens | STEAM | NR | NR | NR | choline-containing compounds | Yes | No | 32 | 1896 |
| van Elst 2005^27^ | 2 | Bruker | PRESS | NR | NR | NR | choline-containing compounds | Yes | No | 20 | 2500 |
| Omori 2000^28^ | 1.5 | GE | PRESS | NR | NR | NR | Choline | No | Yes | 30 | 3000 |
| Ongür 2010^29^ | 4 | Varian | MEGAPRESS OFF | NR | NR | 3.8 | choline-containing compounds | No | Yes | 136 | 2000 |
| Ota 2012^30^ | 1.5 | Siemens | PRESS | NR | NR | NR | Choline | No | No | 68 | 2000 |
| Ota 2015^31^ | 1.5 | Siemens | PRESS | NR | NR | NR | GPC + PCh | No | No | 30 | 1500 |
| Ozcelik 2020^32^ | 1.5 | Siemens | PRESS | NR | NR | NR | GPC + PCh | No | Yes | 35 | 1500 |
| Pae 2004^33^ | 1.5 | GE | STEAM | NR | NR | NR | Choline/phosphocholine | No | Yes | 20 | 2000 |
| Plitman 2018^34^ | 3 | GE | STEAM | NR | NR | 13.75 | GPC + PCh | Yes | No | 35 | 2000 |
| Plitman 2016^36^ | 3 | GE | PRESS | 9.856 | NR | 14.32 | GPC + PCh | Yes | Yes | 35 | 2000 |
| Reid 2013^37^ | 3 | Siemens | PRESS | 9.856 | 7.9 | 7.84 | Choline | No | Yes | 80 | 2000 |
| Reid 2010^38^ | 3 | Siemens | PRESS | 9.86 | NR | NR | Choline | No | Yes | 80 | 2000 |
| Reid 2016^39^ | 3 | Siemens | PRESS | NR | 2.6 | 10.8 | Choline | Yes | Yes | 80 | 2000 |
| Rowland 2013^40^ | 3 | Philips | PRESS | 4.9 | NR | NR | GPC + PCh | Yes | No | 35 | 2000 |
| Rowland 2009^41^ | 3 | Philips | PRESS | NR | NR | NR | choline-containing compounds | Yes | No | 35 | 2000 |
| Sarramea-Crespo 2008^42^ | 1.5 | GE | PRESS | NR | NR | NR | Choline | Yes | Yes | 35 | 1500 |
| Seese 2011^43^ | 1.5 | Siemens | PRESS | NR | NR | NR | choline compounds | Yes | No | 30 | 1500 |
| Shakory 2018^45^ | 3 | GE | PRESS | NR | NR | 14.36 | GPC+PCh | Yes | No | 35 | 2000 |
| Shakory 2018^45^ | 3 | GE | PRESS | 7.27 | NR | 13.63 | GPC+PCh | Yes | No | 35 | 2000 |
| Sharma 1992^47^ | 1.5 | GE | STEAM | 7.27 | NR | NR | choline containing compounds | No | Yes | 28.5 | 3000 |
| Shiori 1996^48^ | 1.5 | GE | STEAM | NR | NR | NR | choline-containing compounds | No | Yes | 135 | 2000 |
| Shirayama 2010^49^ | 3 | GE | PRESS | NR | 3 | NR | GPC+PCh | No | Yes | 30 | 5000 |
| Sigmundsson 2003^50^ | 1.5 | GE | PRESS | NR | NR | NR | Choline | No | No | 136 | 2000 |
| Singh 2018^51^ | 3 | Siemens | PRESS | NR | NR | NR | GPC+PCh | No | Yes | 33 | 2000 |
| Sivaraman 2017^52^ | 3 | Siemens | PRESS | NR | 2.53 | 15.49 | Choline | Yes | No | 80 | 2000 |
| Smesny 2022^53^ | 3 | Siemens | PRESS | 8.48 | NR | 21 | GPC, PCh | Yes | No | 30 | 2000 |
| Stanley 1995^55^ | 1.5 | Siemens | STEAM | 6.1 | NR | NR | choline-containing compounds | Yes | No | 20 | 1500 |
| Stanley 1995^55^ | 1.5 | Siemens | STEAM | NR | NR | NR | choline-containing compounds | Yes | No | 20 | 1500 |
| Stone 2009^56^ | 3 | GE | PRESS | NR | NR | 19 | GPC | Yes | No | 30 | 3000 |
| Szulc 2011^58^ | 1.5 | Picker | PRESS | 5.3 | NR | NR | choline-containing compounds | No | Yes | 35 | 1500 |
| Tanaka 2006^59^ | 1.5 | GE | PRESS | NR | NR | NR | choline-containing compounds | No | No | 30 | 3000 |
| Tarumi 2020^60^ | 3 | GE | PRESS | NR | NR | 22 | GPC + PCh | Yes | No | 35 | 2000 |
| Tarumi 2020^60^ | 3 | GE | PRESS | 3.70 | NR | 21.1 | GPC + PCh | Yes | No | 35 | 2000 |
| Tayoshi 2009^62^ | 3 | GE | STEAM | 3.70 | NR | NR | choline-containing compounds | Yes | No | 18 | 5000 |
| Tibbo 2000^63^ | 3 | Magnex | PRESS | NR | NR | NR | Choline | No | Yes | 120 | 2000 |
| Tibbo 2013^64^ | 3 | Magnex | STEAM | NR | NR | NR | Choline | Yes | No | 240 | 3000 |
| Uhl 2011^65^ | 1.5 | Siemens | PRESS | NR | NR | NR | Choline | No | Yes | 140 | 1500 |
| Uhl 2011^65^ | 1.5 | Siemens | PRESS | NR | NR | NR | Choline | No | Yes | 140 | 1500 |
| Vingerhoets 2019^66^ | 3 | Philips | PRESS | NR | NR | NR | GPC + PCh | Yes | Yes | 45 | 2000 |
| Wang 2019^68^ | 7 | Philips | STEAM | NR | 2.6 | 49.2 | tCho (phosphocholine plus glycerophosphocholine) | Yes | No | 14 | 3000 |
| Wang 2022^69^ | 3 | Siemens | PRESS | 9.3 | NR | 21.47 | GPC + PCh | Yes | No | 30 | 3000 |
| Wijtenburg 2021^70^ | 7 | Philips | STEAM | 9.24 | NR | 51.2 | total choline (phosphocholine + glycerophosphorylcholine) | Yes | No | 14 | 3000 |
| Wijtenburg 2017^71^ | 3 | Siemens | PR-STEAM | 9.6 | NR | NR | total choline (phosphocholine + glycerophosphorylcholine) | No | Yes | 6.5 | 2000 |
| Wood 2003^72^ | 1.5 | GE | PRESS | 4.68 | NR | NR | choline-containing compounds | No | Yes | 135 | 1500 |
| Wood 2003^72^ | 1.5 | GE | PRESS | NR | NR | NR | choline-containing compounds | No | Yes | 135 | 1500 |
| Yamasue 2003^73^ | 1.5 | GE | PRESS | NR | NR | NR | choline-containing compounds | No | Yes | 35 | 2000 |
| Yamasue 2002^74^ | 1.5 | GE | PRESS | NR | NR | NR | Choline | No | Yes | 35 | 2000 |
| Yasukawa 2005^75^ | 1.5 | GE | PRESS | NR | NR | NR | choline-containing compounds | Yes | No | 30 | 1500 |
| Zabala 2007^76^ | 1.5 | Philips | PRESS | NR | NR | NR | Choline | Yes | No | 136 | 1500 |
| Ongür 2008^77^ | 4 | Varian | PRESS | NR | 6 | NR | Choline | Yes | No | 30-500 | 2000 |
| Aoyama 2011^78^ | 4 | Varian | STEAM | 9.4 | NR | NR | Choline | Yes | No | 20 | 2000 |
| Birur 2020^79^ | 3 | Siemens | PRESS | NR | 3.24 | 13.72 | Choline | No | Yes | 80 | 2000 |
| Bluml 1999^80^ | 1.5 | NR | PRESS | 6.86 | NR | NR | total choline | Yes | No | 30 | 1500 |
| Bossong 2019^81^ | 3 | GE | PRESS | NR | 4.2 | 13.3 | GPC | Yes | No | 30 | 3000 |
| Bustillo 2011^83^ | 4 | Bruker | PEPSI | NR | NR | NR | Choline | Yes | No | 15 | 2000 |
| Bustillo 2010^84^ | 4 | Varian | STEAM | NR | NR | NR | Choline | No | No | 20 | 2000 |
| Da Silva 2019^85^ | 3 | GE | MEGA-PRESS OFF | 7.57 | NR | NR | total choline | NR | Yes | 68 | 1500 |
| Deicken 2001^86^ | 1.5 | Siemens | PRESS | NR | NR | NR | Choline | Yes | No | 135 | 1960 |
| Deicken 1999^87^ | 1.5 | Siemens | PRESS | NR | NR | NR | Choline | NR | No | 135 | 1800 |
| Demjaha 2014^88^ | 3 | GE | PRESS | NR | NR | NR | Choline | NR | No | 30 | 3000 |
| Demjaha 2014^88^ | 3 | GE | PRESS | NR | NR | NR | Choline | NR | No | 30 | 3000 |
| Egerton 2018^91^ | 3 | NR | PRESS | 4.93 | 3.73 | 22.54 | total choline | No | Yes | 30 | 3000 |
| Gan 2017^92^ | 1.5 | Siemens | NR | NR | NR | NR | Choline | No | Yes | 35 | 1500 |
| Mcilwain 2015^93^ | 3 | Siemens | PRESS | NR | NR | NR | Choline | Yes | Yes | 30 | 2000 |
| Mcilwain 2015^93^ | 3 | Siemens | PRESS | NR | NR | NR | Choline | Yes | Yes | 30 | 2000 |
| Kragulijac 2013^94^ | 3 | Siemens | PRESS | 8.86 | NR | 11.39 | Choline | No | Yes | 80 | 2000 |
| Larabi 2015^95^ | 3 | Philips | PRESS | 4.93 | 4.08 | 20.92 | total choline (GPC + PCh) | Yes | No | 144 | 2000 |
| Lim 1998^96^ | 1.5 | GE | NR | NR | NR | NR | Choline | No | No | 144 | 2000 |
| Auer 2001^97^ | 1.5 | GE | PRESS | NR | NR | NR | choline-containing compounds | No | No | 35 | 2000 |
| Aydin 2008^98^ | 1.5 | Siemens | STEAM | 2.55 | NR | 16.7 | Choline between 3.19 ppm and 3.24 ppm | No | No | 30-300 | 3500 |
| Aydin 2008^98^ | 1.5 | Siemens | STEAM | 2.55 | NR | 16.7 | Choline between 3.19 ppm and 3.24 ppm | No | No | 30-300 | 3500 |
| Bartolomeo 2019^99^ | 3 | Siemens | PRESS | NR | NR | NR | composed of PCh and GPC | Yes | No | 30 | 1500 |
| Bertolino 1998^100^ | 1.5 | GE | spin-echo MRSI | NR | NR | NR | Choline | No | Yes | 272 | 2200 |
| Bertolino 1996^101^ | 1.5 | GE | spin-echo MRSI | NR | NR | NR | choline-containing compounds | No | Yes | 272 | 2200 |
| Blasi 2004^102^ | 1.5 | GE | spin-echo MRSI | NR | NR | NR | choline-containing compounds | No | Yes | 272 | 2200 |
| Block 2000^103^ | 1.5 | Philips | PRESS | NR | NR | NR | choline-containing compounds | No | Yes | 30-272 | 2000 |
| Brandt 2016^105^ | 7 | Philips | STEAM | NR | NR | NR | total choline (GPC + PCh) | No | No | 14 | 3000 |
| Brooks 1998^106^ | 1.5 | GE | PRESS | NR | NR | NR | choline-containing compounds | No | Yes | 136 | 2000 |
| Bustillo 2014^107^ | 1.5 | Philips | PRESS | 6.16 | 2.9 | 36.3 | GPC + PCh | Yes | No | 40 | 1500 |
| Bustillo 2017^108^ | 7 | Philips | STEAM | 4.56 | NR | 14.8 | GPC + PCh | Yes | No | 40 | 1500 |
| Bustillo 2019^109^ | 1.5 | GE | PRESS | 4.68 | NR | 14.6 | GPC + PCh | Yes | No | 40 | 1500 |
| Bustillo 2001^110^ | 3 | Siemens | PRESS | NR | NR | NR | choline containing compounds | Yes | No | 30 | 2000 |
| Bustillo 2002a^111^ | 3 | Siemens | PRESS | NR | NR | NR | Choline | Yes | No | 40 | 2000 |
| Bustillo 2008^112^ | 3 | Siemens | PRESS | 4.85 | NR | 7.5 | PCh | Yes | No | 40 | 2000 |
| Bustillo 2002b^114^ | 1.5 | GE | STEAM | NR | NR | NR | Choline | Yes | No | 40 | 2000 |
| Byun 2009^115^ | 1.5 | Siemens | PRESS | NR | NR | NR | Choline | Yes | No | 40 | 6000 |
| Callicott 1998^116^ | 1.5 | GE | MRSI | NR | NR | NR | choline-containing compounds | No | Yes | 272 | 2200 |
| Cecil 1999^117^ | 1.5 | GE | STEAM | NR | NR | NR | choline-containing compounds (3.2 ppm) | No | Yes | 21 | 2000 |
| Chang 2007^118^ | 4 | Varian | NR | NR | NR | NR | choline-containing compounds | Yes | No | 30 | 3000 |
| Chiu 2018^119^ | 3 | Philips | PRESS | NR | 4.88 | NR | Choline | Yes | No | 39 | 2000 |
| Crocker 2014^120^ | 3 | Magnex | STEAM | 1.67 | NR | 14.3 | choline containing compounds | Yes | No | 240 | 3000 |
| de la Fuente Sandoval 2013^121^ | 3 | GE | PRESS | 6.16 | 3.44 | 17 | GPC+ PCh | Yes | No | 35 | 2000 |
| de la Fuente Sandoval 2011^122^ | 3 | GE | PRESS | 8.62 | NR | NR | GPC+ PCh | Yes | No | 35 | 2000 |
| de la Fuente Sandoval 2018^123^ | 3 | GE | MEGA-PRESS | NR | NR | 18.77 | total choline | Yes | No | 68 | 1500 |
| de la Fuente Sandoval 2015^124^ | 3 | GE | MEGA-PRESS | 11.63 | NR | 18.93 | total choline | Yes | Yes | 68 | 1500 |
| Deicken 2000^125^ | 1.5 | Siemens | NR | NR | NR | NR | Choline | No | No | 135 | 1960 |
| Deicken 1997a^126^ | 1.5 | Siemens | PRESS | NR | NR | NR | Choline | No | No | 135 | 1800 |
| Deicken 1998^127^ | 1.5 | Siemens | PRESS | NR | NR | NR | Choline | No | Yes | 135 | 1800 |
| Deicken 1997b^128^ | 1.5 | Siemens | PRESS | NR | NR | NR | Choline | No | No | 135 | 1800 |
| Delamillieure 2000a^129^ | 1.5 | GE | STEAM | NR | NR | NR | Choline | No | Yes | 30 | 1500 |
| Delamillieure 2002^130^ | 1.5 | GE | STEAM | NR | NR | NR | Choline | No | Yes | 30 | 1500 |
| Delamillieure 2000b^131^ | 1.5 | GE | STEAM | NR | NR | NR | Choline | No | Yes | 30 | 1500 |
| Ende 2001^132^ | 1.5 | Siemens | PRESS | NR | NR | NR | choline-containing compounds | No | No | 135 | 1500 |
| Fannon 2003^133^ | 1.5 | GE | PRESS | NR | NR | NR | choline-containing compounds | Yes | Yes | 35 | 1500 |
| Fujimoto 1996^134^ | 2 | Siemens | NR | NR | NR | NR | choline-containing compounds | No | Yes | 35 | 1500 |
| Fukuzako 2000^135^ | 2 | Siemens | STEAM | NR | NR | NR | Choline containing compounds | No | Yes | 135 | 1500 |
| Fukuzako 1995^136^ | 2 | Siemens | STEAM | NR | NR | NR | Choline containing compounds | No | Yes | 60 | 2000 |
| Galinska 2009^137^ | 1.5 | Picker eclipse | PRESS | NR | NR | NR | Choline containing compounds | Yes | Yes | 135 | 1500 |
| Galinska-Skok 2018^139^ | 1.5 | Picker eclipse | PRESS | NR | NR | NR | Choline containing compounds | Yes | Yes | 35 | 1500 |
| Gallinat 2016^140^ | 3 | Bruker biospin | NR | NR | NR | NR | Choline | Yes | No | 35 | 1500 |
| Gan 2014^141^ | 1.5 | Siemens | NR | NR | NR | NR | Choline | Yes | Yes | 135 | 1500 |
| Granata 2013^142^ | 1.5 | Siemens | NR | NR | NR | NR | Choline containing compounds | Yes | Yes | 135 | 1365 |
| Hagino 2002^143^ | 1.5 | Siemens | PRESS | NR | NR | NR | Choline containing compounds | No | Yes | 270 | 1500 |
| Hasan 2014^144^ | 1.5 | Siemens | NR | NR | NR | NR | Choline containing compounds | Yes | Yes | 30 | 1500 |
| He 2018^145^ | 3 | Siemens | PRESS | NR | NR | NR | Choline | Yes | Yes | 30 | 3000 |
| He 2012^146^ | 3 | GE | PRESS | NR | NR | NR | Choline containing compounds | Yes | Yes | 35 | 1500 |
| Heimberg 1998^147^ | 1.5 | GE | STEAM | NR | NR | NR | Choline containing compounds | Yes | Yes | 30 | 2000 |
| Huang 2019^148^ | 3 | Phillips | CHESS | NR | NR | NR | Choline containing compounds | Yes | Yes | 9.2 | 2000 |
| Huang 2017^149^ | 3 | Phillips | CHESS | NR | NR | NR | Choline containing compounds | Yes | Yes | 9.2 | 2000 |
| Iwata 2019^150^ | 3 | GE | NR | 9.3 | NR | 12.3 | GPC+ PCh | Yes | No | 35 | 2000 |
| Iwata 2019^150^ | 3 | GE | NR | 8.6 | NR | 12.5 | GPC+ PCh | Yes | No | 35 | 2000 |
| Jakary 2005^151^ | 1.5 | Siemens | NR | NR | NR | NR | Choline containing compounds | Yes | No | 135 | 1960 |
| Jessen 2013^152^ | 3 | Phillips | PRESS | NR | NR | NR | Choline containing compounds | Yes | No | 140 | 2000 |
| Jessen 2006^153^ | 1.5 | Phillips | PRESS | NR | NR | NR | Choline containing compounds | Yes | Yes | 272 | 2000 |
| Jessen 2006^153^ | 1.5 | Phillips | PRESS | NR | NR | NR | Choline containing compounds | Yes | Yes | 272 | 2000 |
| Kegeles 2000^155^ | 1.5 | GE | STEAM | NR | NR | NR | Choline containing compounds | Yes | Yes | 20 | NR |
| Kim 2018^156^ | 4 | Varian | PRESS | NR | NR | NR | GPC+ PCh | Yes | Yes | NR | 2000 |
| Kirtas 2016^157^ | 1.5 | GE | NR | NR | NR | NR | GPC+ PCh | Yes | Yes | 85 | 3000 |
| Kirtas 2016^157^ | 1.5 | GE | NR | NR | NR | NR | GPC+ PCh | Yes | Yes | 85 | 3000 |
| Klar 2010^158^ | 3 | Bruker biospin | PRESS | NR | NR | NR | Total choline | Yes | Yes | 80 | 3000 |
| Kragulijac 2019^159^ | 3 | Siemens | PRESS | 4.82 | 2.62 | 10.97 | Choline | Yes | Yes | 80 | 2000 |
| Lebedeva 2014^160^ | 3 | Phillips | PRESS | NR | NR | NE | Choline containing compounds | Yes | No | 35 | 2000 |
| Legind 2019^161^ | 3 | Phillips | PRESS | 7.39 | 5.76 | 10.91 | Choline | Yes | Yes | 30 | 3000 |
| Bartha 1997^162^ | 1.5 | Siemens | STEAM | 5.74 | NR | 20 | Choline containing compounds | No | No | 20 | 1500 |
| Basoglu 2006^163^ | 1.5 | Siemens | PRESS | NR | NR | NR | Choline | No | Yes | 135 | 1600 |
| Basoglu 2006^163^ | 1.5 | Siemens | PRESS | NR | NR | NR | Choline | No | Yes | 135 | 1600 |
| Chiappelli 2015^164^ | 3 | Siemens | PRESS | NR | 4.3 | NR | GPC+ PCh | No | No | 30 | 2000 |
| Choe 1994^165^ | 1.5 | GE | NR | NR | NR | NR | Choline containing compounds | No | Yes | 30 | 2000 |
| Maier 2000^166^ | 1.5 | GE | STEAM | NR | NR | NR | Choline | No | No | 10 | 6000 |
| Premkumar 2010^167^ | 1.5 | GE | NR | NR | NR | NR | GPC+ PCh | Yes | No | 35 | 2000 |
| Stanley 2007^168^ | 1.5 | GE | STEAM | NR | NR | NR | GPC+ PCh | Yes | No | 20 | 6000 |
| Taylor 2017^169^ | 7 | Siemens | STEAM | NR | NR | NR | Choline | Yes | No | 10 | 300 |
| Terpstra 2005^170^ | 4 | Siemens | STEAM | NR | NR | NR | GPC | Yes | No | 5 | 450 |
| Theberge 2007^171^ | 4 | Siemens | STEAM | NR | NR | NR | Choline containing compounds | Yes | No | 20 | 2000 |
| Tunc-Skarka 2009^172^ | 3 | Siemens | PRESS | NR | NR | NR | GPC+ PCh | Yes | No | 30 | 6000 |
| Venkatraman 2006^173^ | 4 | GE | PRESS | NR | NR | NR | GPC+ PCh | Yes | No | 30 | 3000 |
| Ueno 2022^174^ | 4 | Siemens | STEAM | NR | 2.13 | NR | GPC+ PCh | Yes | Yes | 68 | 1500 |
| Ueno 2022^174^ | 3 | Siemens | PRESS | NR | 2.13 | NR | GPC+ PCh | Yes | Yes | 68 | 1500 |
| Cadenhead 2023^175^ | 3 | GE | PRESS | NR | NR | 17.18 | GPC+ PCh | Yes | No | 30 | 1500 |
| Chiappelii 2024^176^ | 3 | Siemens | PRESS | NR | NR | NR | Choline | Yes | No | 30 | 2000 |
| Fan 2024^177^ | 3 | Siemens | PRESS | 6.16 | 3.05 | 26.35 | GPC+ PCh | Yes | Yes | 30 | 3000 |
| Fan 2024^177^ | 3 | Siemens | PRESS | 6.16 | 3.19 | 25.08 | GPC+ PCh | Yes | Yes | 30 | 3000 |
| Koster 2024^178^ | 3 | Philips | PRESS | 26.8 | 2 | 15.5 | GPC+ PCh | Yes | Yes | 35 | 2000 |
| León-Ortiz 2023^179^ | 3 | Siemens | PRESS | 9.86 | 3.08 | 28.69 | phosphocholine + choline | Yes | No | 35 | 2000 |
| Wang 2023^180^ | 7 | Philips | STEAM | NR | 2.29 | NR | GPC+ PCh | Yes | No | 14 | 3000 |
| Allam 2024^181^ | 1.5 | GE | CHESS | NR | NR | NR | Choline | No | Yes | 35 | 1600 |
| Allam 2024^181^ | 1.5 | GE | CHESS | NR | NR | NR | Choline | No | Yes | 35 | 1600 |
| Kubota 2023^183^ | 3 | Siemens | SPECIAL | NR | 4 | NR | GPC + PC | Yes | No | 8.5 | 3000 |
| Stanley 2023^184^ | 3 | Siemens | PRESS | 5.3 | NR | NR | GPC + PC | Yes | No | 23 | 3370 |
| Olbrich 2008^186^ | 2 | MedSpec | PRESS | NR | NR | NR | Choline | Yes | No | 5.5 | 1700 |

**Supplementary table 3 – MRS Quality parameters of included studies.** FWHM indicates full width at half maximum; CRLB, Cramér–Rao lower bound; SNR, signal-to-noise ratio; CSF, cerebrospinal fluid; TE, echo time; TR, repetition time; PRESS, point-resolved spectroscopy; STEAM, stimulated echo acquisition mode; LASER, localization by adiabatic selective refocusing; MEGA-PRESS, mescher–garwood point-resolved spectroscopy; CHESS, chemical shift selective suppression; ¹H-MRS, proton magnetic resonance spectroscopy; tCho, total choline; GPC, glycerophosphocholine; PCh, phosphocholine; NR, not reported**.**

| **Psychosis spectrum disorders** | | | | | | | | |
| --- | --- | --- | --- | --- | --- | --- | --- | --- |
| **Region** | **Cohorts** | **Cases** | **Controls** | **VR** | **Lower 95% CI** | **Upper 95% CI** | **P value** | **I2 (%)** |
| ACC | 65 | 2010 | 1676 | 0.09 | -0.01 | 0.18 | 0.084 | 74.2 |
| dlPFC | 39 | 1013 | 939 | 0.08 | -0.01 | 0.17 | 0.091 | 43.0 |
| Occipital lobe | 10 | 211 | 241 | 0.18 | -0.06 | 0.42 | 0.141 | 63.5 |
| Hippocampus | 39 | 1070 | 868 | 0.20 | 0.07 | 0.32 | 0.002 | 68.4 |
| mPFC | 27 | 1006 | 828 | 0.06 | -0.06 | 0.17 | 0.316 | 59.6 |
| Frontal white matter | 24 | 566 | 521 | -0.02 | -0.15 | 0.11 | 0.766 | 48.4 |
| Thalamus | 35 | 1033 | 914 | 0.07 | -0.04 | 0.18 | 0.207 | 60.3 |
| Striatum | 36 | 798 | 696 | -0.03 | -0.22 | 0.16 | 0.754 | 83.2 |
| Cerebellum | 7 | 158 | 131 | -0.12 | -0.41 | 0.16 | 0.408 | 61.9 |
| Temporal lobe | 22 | 567 | 440 | -0.03 | -0.16 | 0.11 | 0.701 | 49.7 |
| Other frontal lobe | 16 | 322 | 288 | 0.11 | -0.00 | 0.23 | 0.057 | 0.0 |
| Parietal lobe | 10 | 205 | 172 | -0.04 | -0.24 | 0.16 | 0.678 | 40.1 |
| Centrum semiovale | 6 | 218 | 244 | 0.12 | -0.01 | 0.25 | 0.079 | 0.0 |
| Global grey matter | 3 | 177 | 170 | 0.86 | -0.56 | 2.28 | 0.237 | 98.7 |
| Global white matter | 3 | 177 | 170 | 0.22 | -0.15 | 0.60 | 0.241 | 80.7 |
| Corpus callosum | 3 | 43 | 25 | 0.09 | -0.29 | 0.48 | 0.650 | 0.0 |
| PCC | 4 | 116 | 144 | 0.23 | 0.05 | 0.41 | 0.011 | 0.0 |

**Supplementary table 4: VR Meta-analysis Results Summary for Psychosis spectrum disorders in All Brain Regions**

| **CHR** | | | | | | | | |
| --- | --- | --- | --- | --- | --- | --- | --- | --- |
| **Region** | **Studies** | **Cases** | **Controls** | **VR** | **Lower 95% CI** | **Upper 95% CI** | **P value** | **I2 (%)** |
| ACC | 8 | 319 | 224 | -0.09 | -0.23 | 0.06 | 0.246 | 21.5 |
| Hippocampus | 4 | 164 | 81 | 0.17 | -0.09 | 0.42 | 0.197 | 34.9 |
| mPFC | 6 | 233 | 153 | 0.21 | 0.06 | 0.36 | 0.006 | 0.0 |
| Thalamus | 3 | 116 | 108 | 0.14 | -0.17 | 0.44 | 0.378 | 54.8 |
| Striatum | 2 | 41 | 64 | -1.26 | -3.56 | 1.02 | 0.280 | 98.4 |
| Temporal lobe | 2 | 73 | 27 | 0.48 | 0.14 | 0.83 | 0.006 | 0.0 |

**Supplementary table 5: VR Meta-analysis Results Summary for CHR in All Brain Regions**

| **Established psychosis** | | | | | | | | |
| --- | --- | --- | --- | --- | --- | --- | --- | --- |
| **Region** | **Cohorts** | **Cases** | **Controls** | **VR** | **Lower 95% CI** | **Upper 95% CI** | **P value** | **I2 (%)** |
| ACC | 52 | 1570 | 1477 | 0.11 | -0.01 | 0.23 | 0.069 | 78.7 |
| dlPFC | 36 | 955 | 919 | 0.07 | -0.03 | 0.17 | 0.151 | 45.2 |
| Occipital lobe | 10 | 211 | 241 | 0.18 | -0.06 | 0.42 | 0.141 | 63.5 |
| Hippocampus | 35 | 906 | 811 | 0.20 | 0.07 | 0.33 | 0.003 | 70.8 |
| mPFC | 21 | 773 | 699 | 0.02 | -0.12 | 0.15 | 0.832 | 66.3 |
| Frontal white matter | 24 | 566 | 521 | -0.02 | -0.15 | 0.11 | 0.766 | 48.4 |
| Thalamus | 32 | 917 | 806 | 0.06 | -0.05 | 0.18 | 0.291 | 61.0 |
| Striatum | 31 | 693 | 632 | 0.05 | -0.08 | 0.18 | 0.481 | 59.0 |
| Cerebellum | 6 | 140 | 91 | -0.03 | -0.28 | 0.22 | 0.800 | 37.7 |
| Temporal lobe | 20 | 494 | 440 | -0.07 | -0.21 | 0.060 | 0.278 | 43.9 |
| Other frontal lobe | 15 | 303 | 288 | 0.11 | -0.01 | 0.23 | 0.073 | 0.0 |
| Parietal lobe | 10 | 205 | 172 | -0.04 | -0.24 | 0.16 | 0.678 | 40.1 |
| Centrum semiovale | 6 | 218 | 244 | 0.12 | -0.01 | 0.25 | 0.079 | 0.0 |
| Global grey matter | 3 | 177 | 170 | 0.86 | -0.56 | 2.28 | 0.237 | 98.7 |
| Global white matter | 3 | 177 | 170 | 0.22 | -0.15 | 0.60 | 0.241 | 80.7 |
| Corpus callosum | 2 | 26 | 25 | 0.17 | -0.23 | 0.58 | 0.408 | 0.0 |
| PCC | 4 | 116 | 144 | 0.23 | 0.05 | 0.41 | 0.011 | 0.0 |

**Supplementary table 6: VR Meta-analysis Results Summary for Established psychosis in All Brain Regions**

| **TRS** | | | | | | | | |
| --- | --- | --- | --- | --- | --- | --- | --- | --- |
| **Region** | **Studies** | **Cases** | **Controls** | **VR** | **Lower 95% CI** | **Upper 95% CI** | **P value** | **I2 (%)** |
| ACC | 5 | 121 | 115 | 0.25 | -0.14 | 0.64 | 0.212 | 74.7 |
| dlPFC | 2 | 38 | 42 | 0.24 | -0.26 | 0.74 | 0.352 | 57.7 |
| Striatum | 3 | 64 | 65 | 0.04 | -0.39 | 0.47 | 0.861 | 65.1 |

**Supplementary table 7: VR Meta-analysis Results Summary for TRS in All Brain Regions**

| **CHR** | | | | | | | | |
| --- | --- | --- | --- | --- | --- | --- | --- | --- |
| **Region** | **Studies** | **Cases** | **Controls** | **SMD** | **Lower 95% CI** | **Upper 95% CI** | **P value** | **I2 (%)** |
| ACC | 8 | 319 | 288 | 0.19 | 0.03 | 0.36 | 0.020 | 0.3 |
| Hippocampus | 4 | 164 | 105 | 0.15 | -0.11 | 0.40 | 0.253 | 0.0 |
| mPFC | 6 | 233 | 175 | 0.36 | 0.16 | 0.57 | <0.001 | 3.5 |
| Thalamus | 3 | 116 | 108 | -0.09 | -0.38 | 0.20 | 0.546 | 12.8 |
| Striatum | 2 | 41 | 64 | 0.21 | -0.19 | 0.61 | 0.296 | 0.0 |
| Temporal lobe | 2 | 73 | 52 | -0.06 | -0.49 | 0.37 | 0.794 | 19.5 |

**Supplementary table 8: SMD Meta-analysis Results Summary for CHR in All Brain Regions**

| **Established psychosis** | | | | | | | | |
| --- | --- | --- | --- | --- | --- | --- | --- | --- |
| **Region** | **Cohorts** | **Cases** | **Controls** | **SMD** | **Lower 95% CI** | **Upper 95% CI** | **P value** | **I2 (%)** |
| ACC | 52 | 1570 | 1477 | 0.21 | 0.09 | 0.33 | <0.001 | 59.5 |
| dlPFC | 36 | 955 | 919 | 0.11 | -0.02 | 0.24 | 0.107 | 45.6 |
| Occipital lobe | 10 | 211 | 241 | 0.24 | 0.05 | 0.43 | 0.012 | 0.0 |
| Hippocampus | 35 | 906 | 811 | 0.08 | -0.03 | 0.18 | 0.151 | 12.9 |
| mPFC | 21 | 773 | 699 | 0.08 | -0.05 | 0.21 | 0.220 | 25.8 |
| Frontal white matter | 24 | 566 | 521 | 0.03 | -0.11 | 0.18 | 0.653 | 23.9 |
| Thalamus | 32 | 917 | 806 | -0.10 | -0.22 | 0.01 | 0.083 | 26.0 |
| Striatum | 31 | 693 | 632 | 0.25 | 0.10 | 0.40 | <0.001 | 40.3 |
| Cerebellum | 6 | 140 | 91 | -0.14 | -0.48 | 0.20 | 0.426 | 37.3 |
| Temporal lobe | 20 | 494 | 440 | -0.11 | -0.35 | 0.13 | 0.366 | 66.0 |
| Other frontal lobe | 15 | 303 | 288 | 0.32 | 0.10 | 0.54 | 0.004 | 39.0 |
| Parietal lobe | 10 | 205 | 172 | 0.46 | 0.04 | 0.88 | 0.033 | 73.1 |
| Centrum semiovale | 6 | 218 | 244 | 0.18 | -0.01 | 0.36 | 0.061 | 0.0 |
| Global grey matter | 3 | 177 | 170 | 0.11 | -0.11 | 0.32 | 0.331 | 0.0 |
| Global white matter | 3 | 177 | 170 | 0.06 | -0.15 | 0.28 | 0.551 | 0.0 |
| Corpus callosum | 2 | 26 | 25 | -0.33 | -0.91 | 0.25 | 0.264 | 8.4 |
| PCC | 4 | 116 | 144 | 0.33 | -0.25 | 0.90 | 0.265 | 77.9 |

**Supplementary table 9: SMD Meta-analysis Results Summary for established psychosis in All Brain Regions**

| **TRS** | | | | | | | | |
| --- | --- | --- | --- | --- | --- | --- | --- | --- |
| **Region** | **Studies** | **Cases** | **Controls** | **SMD** | **Lower 95% CI** | **Upper 95% CI** | **P value** | **I2 (%)** |
| ACC | 5 | 121 | 115 | 0.65 | 0.38 | 0.91 | <0.001 | 0.0 |
| dlPFC | 2 | 38 | 42 | 1.13 | 0.65 | 1.60 | <0.001 | 0.0 |
| Striatum | 3 | 64 | 65 | 0.29 | -0.24 | 0.83 | 0.278 | 55.6 |

**Supplementary table 10: SMD Meta-analysis Results Summary for TRS in All Brain Regions**

| **CHR** | | | | | | | | |
| --- | --- | --- | --- | --- | --- | --- | --- | --- |
| **Region** | **Studies** | **Cases** | **Controls** | **CVR** | **Lower 95% CI** | **Upper 95% CI** | **P value** | **I2 (%)** |
| ACC | 8 | 319 | 288 | -0.10 | -0.27 | 0.08 | 0.293 | 48.3 |
| Hippocampus | 4 | 164 | 105 | 0.14 | -0.09 | 0.37 | 0.240 | 32.6 |
| mPFC | 6 | 233 | 175 | 0.15 | 0.00 | 0.30 | 0.050 | 0.0 |
| Thalamus | 3 | 116 | 108 | 0.14 | -0.21 | 0.49 | 0.421 | 65.1 |
| Striatum | 2 | 41 | 64 | -1.33 | -3.65 | 0.99 | 0.260 | 98.1 |
| Temporal lobe | 2 | 73 | 52 | 0.53 | 0.16 | 0.90 | 0.004 | 37.5 |

**Supplementary table 11: CVR Meta-analysis Results Summary for CHR in All Brain Regions**

| **Established psychosis** | | | | | | | | |
| --- | --- | --- | --- | --- | --- | --- | --- | --- |
| **Region** | **Cohorts** | **Cases** | **Controls** | **CVR** | **Lower 95% CI** | **Upper 95% CI** | **P value** | **I2 (%)** |
| ACC | 52 | 1570 | 1477 | 0.09 | -0.02 | 0.19 | 0.098 | 69.6 |
| dlPFC | 36 | 955 | 919 | 0.07 | -0.00 | 0.14 | 0.055 | 0.0 |
| Occipital lobe | 10 | 211 | 241 | 0.13 | -0.13 | 0.37 | 0.310 | 61.7 |
| Hippocampus | 35 | 906 | 811 | 0.16 | 0.05 | 0.27 | 0.004 | 50.3 |
| mPFC | 21 | 773 | 699 | 0.00 | -0.14 | 0.14 | 0.975 | 66.3 |
| Frontal white matter | 24 | 566 | 521 | -0.03 | -0.16 | 0.11 | 0.708 | 47.0 |
| Thalamus | 32 | 917 | 806 | 0.08 | -0.03 | 0.20 | 0.155 | 56.5 |
| Striatum | 31 | 693 | 632 | -0.00 | -0.12 | 0.11 | 0.942 | 46.0 |
| Cerebellum | 6 | 140 | 91 | -0.01 | -0.28 | 0.26 | 0.945 | 43.1 |
| Temporal lobe | 20 | 494 | 440 | -0.03 | -0.15 | 0.08 | 0.569 | 25.6 |
| Other frontal lobe | 15 | 303 | 288 | 0.04 | -0.08 | 0.17 | 0.481 | 0.0 |
| Parietal lobe | 10 | 205 | 172 | -0.11 | -0.29 | 0.08 | 0.250 | 25.7 |
| Centrum semiovale | 6 | 218 | 244 | 0.09 | -0.04 | 0.23 | 0.172 | 0.0 |
| Global grey matter | 3 | 177 | 170 | 0.81 | -0.62 | 2.25 | 0.267 | 97.8 |
| Global white matter | 3 | 177 | 170 | 0.22 | -0.14 | 0.57 | 0.226 | 78.0 |
| Corpus callosum | 2 | 26 | 25 | 0.19 | -0.22 | 0.60 | 0.369 | 0.0 |
| PCC | 4 | 116 | 144 | 0.16 | -0.02 | 0.34 | 0.081 | 0.0 |

**Supplementary table 12: CVR Meta-analysis Results Summary for established psychosis in All Brain Regions**

| **TRS** | | | | | | | | |
| --- | --- | --- | --- | --- | --- | --- | --- | --- |
| **Region** | **Studies** | **Cases** | **Controls** | **CVR** | **Lower 95% CI** | **Upper 95% CI** | **P value** | **I2 (%)** |
| ACC | 5 | 121 | 115 | 0.17 | -0.24 | 0.57 | 0.421 | 75.3 |
| dlPFC | 2 | 38 | 42 | 0.12 | -0.44 | 0.68 | 0.674 | 65.9 |
| Striatum | 3 | 64 | 65 | 0.01 | -0.49 | 0.51 | 0.972 | 73.6 |

**Supplementary table 13: CVR Meta-analysis Results Summary for TRS in All Brain Regions**

| **Region** | **Cohorts** | **Effect estimate** | **Lower 95% CI** | **Upper 95% CI** | **P value** |
| --- | --- | --- | --- | --- | --- |
| ACC | 65 | -0.018 | -0.079 | 0.044 | 0.572 |
| dlPFC | 39 | 0.054 | -0.037 | 0.145 | 0.245 |
| Occipital lobe | 10 | 0.008 | -0.114 | 0.130 | 0.893 |
| Hippocampus | 39 | 0.027 | -0.053 | 0.106 | 0.513 |
| mPFC | 27 | -0.048 | -0.122 | 0.026 | 0.205 |
| Frontal white matter | 24 | 0.037 | -0.143 | 0.217 | 0.688 |
| Thalamus | 35 | -0.017 | -0.076 | 0.042 | 0.576 |
| Striatum | 36 | 0.022 | -0.160 | 0.204 | 0.812 |
| Cerebellum | 7 | 0.295 | -0.031 | 0.620 | 0.076 |
| Temporal lobe | 22 | 0.252 | -0.128 | 0.633 | 0.194 |
| Other frontal lobe | 16 | 0.348 | -0.044 | 0.740 | 0.082 |
| Parietal lobe | 10 | -0.246 | -0.639 | 0.147 | 0.220 |
| Centrum semiovale | 6 | -0.021 | -0.091 | 0.049 | 0.562 |

**Supplementary table 14: Meta regression of magnet strength for SMD in psychosis spectrum disorders**

| **Region** | **Cohorts** | **Effect estimate** | **Lower 95% CI** | **Upper 95% CI** | **P value** |
| --- | --- | --- | --- | --- | --- |
| ACC | 60 | -0.002 | -0.016 | 0.012 | 0.766 |
| dlPFC | 36 | 0.019 | 0.000 | 0.039 | 0.051 |
| Occipital lobe | 9 | -0.002 | -0.016 | 0.011 | 0.759 |
| Hippocampus | 37 | -0.011 | -0.027 | 0.006 | 0.199 |
| mPFC | 24 | -0.012 | -0.027 | 0.004 | 0.135 |
| Frontal white matter | 24 | 0.010 | -0.002 | 0.022 | 0.094 |
| Thalamus | 31 | 0.005 | -0.012 | 0.022 | 0.583 |
| Striatum | 35 | -0.006 | -0.022 | 0.011 | 0.508 |
| Cerebellum | 5 | -0.020 | -0.088 | 0.048 | 0.571 |
| Temporal lobe | 20 | 0.012 | -0.008 | 0.031 | 0.248 |
| Other frontal lobe | 14 | -0.023 | -0.052 | 0.005 | 0.105 |
| Parietal lobe | 8 | -0.011 | -0.063 | 0.042 | 0.695 |
| Centrum semiovale | 6 | 0.003 | -0.027 | 0.032 | 0.864 |

**Supplementary table 15: Meta regression of mean age for SMD in psychosis spectrum disorders**

| **Region** | **Cohorts** | **Effect estimate** | **Lower 95% CI** | **Upper 95% CI** | **P value** |
| --- | --- | --- | --- | --- | --- |
| ACC | 26 | 0.021 | -0.006 | 0.048 | 0.120 |
| dlPFC | 7 | 0.013 | -0.139 | 0.164 | 0.872 |
| Hippocampus | 10 | -0.022 | -0.067 | 0.024 | 0.354 |
| mPFC | 16 | -0.006 | -0.041 | 0.028 | 0.717 |
| Thalamus | 7 | -0.010 | -0.058 | 0.038 | 0.693 |
| Striatum | 12 | 0.166 | 0.009 | 0.323 | 0.039 |

**Supplementary table 16: Meta regression of FWHM (median) for SMD in psychosis spectrum disorders**

| **Region** | **Cohorts** | **Effect estimate** | **Lower 95% CI** | **Upper 95% CI** | **P value** |
| --- | --- | --- | --- | --- | --- |
| ACC | 20 | 0.002 | -0.009 | 0.014 | 0.702 |
| dlPFC | 6 | 0.004 | -0.055 | 0.064 | 0.886 |
| Hippocampus | 9 | -0.027 | -0.125 | 0.071 | 0.592 |
| mPFC | 14 | -0.002 | -0.014 | 0.010 | 0.752 |
| Thalamus | 6 | -0.042 | -0.108 | 0.024 | 0.216 |
| Striatum | 13 | 0.044 | -0.016 | 0.104 | 0.151 |

**Supplementary table 17: Meta regression of SNR (median) for SMD in psychosis spectrum disorders**

| **Region** | **Cohorts** | **Effect estimate** | **Lower 95% CI** | **Upper 95% CI** | **P value** |
| --- | --- | --- | --- | --- | --- |
| ACC | 60 | -0.001 | -0.010 | 0.008 | 0.798 |
| dlPFC | 34 | 0.005 | -0.005 | 0.016 | 0.328 |
| Occipital lobe | 8 | -0.030 | -0.055 | -0.004 | 0.021 |
| Hippocampus | 35 | -0.008 | -0.016 | -0.001 | 0.031 |
| mPFC | 25 | 0.013 | -0.001 | 0.026 | 0.066 |
| Frontal white matter | 23 | -0.011 | -0.020 | -0.002 | 0.017 |
| Thalamus | 31 | 0.007 | 0.000 | 0.014 | 0.052 |
| Striatum | 34 | -0.005 | -0.016 | 0.006 | 0.347 |
| Cerebellum | 6 | -0.009 | -0.025 | 0.007 | 0.275 |
| Temporal lobe | 18 | -0.006 | -0.019 | 0.007 | 0.361 |
| Other frontal lobe | 14 | -0.005 | -0.022 | 0.012 | 0.572 |
| Parietal lobe | 7 | 0.010 | -0.049 | 0.070 | 0.735 |
| Centrum semiovale | 6 | 0.001 | -0.019 | 0.022 | 0.887 |

**Supplementary table 18: Meta regression of Male sex (percentage) for SMD in psychosis spectrum disorders**

| **Region** | **Cohorts** | **Effect estimate** | **Lower 95% CI** | **Upper 95% CI** | **P value** |
| --- | --- | --- | --- | --- | --- |
| ACC | 48 | 0.000 | -0.004 | 0.003 | 0.944 |
| dlPFC | 32 | -0.006 | -0.009 | -0.002 | 0.001 |
| Occipital lobe | 9 | -0.005 | -0.013 | 0.003 | 0.195 |
| Hippocampus | 32 | 0.003 | 0.000 | 0.005 | 0.029 |
| mPFC | 21 | 0.000 | -0.003 | 0.004 | 0.908 |
| Frontal white matter | 22 | -0.001 | -0.006 | 0.003 | 0.470 |
| Thalamus | 28 | 0.001 | -0.002 | 0.004 | 0.633 |
| Striatum | 31 | 0.002 | -0.001 | 0.006 | 0.165 |
| Cerebellum | 6 | 0.005 | -0.002 | 0.011 | 0.187 |
| Temporal lobe | 20 | 0.000 | -0.006 | 0.005 | 0.870 |
| Other frontal lobe | 14 | -0.005 | -0.010 | 0.001 | 0.100 |
| Parietal lobe | 8 | 0.006 | -0.005 | 0.017 | 0.301 |

**Supplementary table 19: Meta regression of medication free (percentage) for SMD in psychosis spectrum disorders**

| **Region** | **Cohorts** | **Effect estimate** | **Lower 95% CI** | **Upper 95% CI** | **P value** |
| --- | --- | --- | --- | --- | --- |
| ACC | 32 | -0.001 | -0.005 | 0.004 | 0.779 |
| dlPFC | 24 | -0.005 | -0.009 | -0.001 | 0.007 |
| Occipital lobe | 7 | -0.009 | -0.019 | 0.001 | 0.081 |
| Hippocampus | 23 | 0.004 | 0.001 | 0.006 | 0.014 |
| mPFC | 12 | 0.000 | -0.004 | 0.004 | 0.857 |
| Frontal white matter | 19 | -0.002 | -0.007 | 0.002 | 0.345 |
| Thalamus | 22 | -0.001 | -0.004 | 0.003 | 0.645 |
| Striatum | 25 | 0.002 | -0.002 | 0.006 | 0.288 |
| Cerebellum | 5 | 0.005 | -0.003 | 0.013 | 0.261 |
| Temporal lobe | 15 | -0.002 | -0.007 | 0.003 | 0.417 |
| Other frontal lobe | 12 | -0.005 | -0.011 | 0.001 | 0.078 |
| ACC | 32 | -0.001 | -0.005 | 0.004 | 0.779 |

**Supplementary table 20: Meta regression of medication naive (percentage) for SMD in psychosis spectrum disorders**

| **Region** | **Cohorts** | **Effect estimate** | **Lower 95% CI** | **Upper 95% CI** | **P value** |
| --- | --- | --- | --- | --- | --- |
| ACC | 22 | -0.003 | -0.010 | 0.005 | 0.508 |
| mPFC | 7 | 0.009 | 0.000 | 0.019 | 0.059 |
| Thalamus | 5 | 0.000 | -0.008 | 0.008 | 0.966 |
| Striatum | 10 | 0.006 | -0.010 | 0.023 | 0.458 |
| Hippocampus | 8 | 0.007 | -0.006 | 0.021 | 0.286 |

**Supplementary table 21: Meta regression of smoking (percentage) for SMD in psychosis spectrum disorders**

| **Region** | **Cohorts** | **Effect estimate** | **Lower 95% CI** | **Upper 95% CI** | **P value** |
| --- | --- | --- | --- | --- | --- |
| ACC | 19 | -0.123 | -0.245 | 0.000 | 0.049 |
| mPFC | 9 | -0.158 | -0.316 | 0.000 | 0.050 |
| Striatum | 5 | -0.115 | -0.311 | 0.081 | 0.251 |
| dlPFC | 5 | -0.182 | -0.333 | -0.031 | 0.018 |

**Supplementary table 22: Meta regression of CRLB (raw scores) for SMD in psychosis spectrum disorders**

| **Medication free** | | | | | |
| --- | --- | --- | --- | --- | --- |
| **Region** | **Cohorts** | **SMD** | **Lower 95% CI** | **Upper 95% CI** | **P value** |
| Hippocampus | 8 | 0.17 | -0.11 | 0.44 | 0.244 |
| dlPFC | 10 | -0.28 | -0.48 | -0.09 | 0.004 |
| Frontal white matter | 5 | -0.11 | -0.35 | 0.14 | 0.394 |
| Striatum | 12 | 0.39 | 0.14 | 0.65 | 0.003 |
| mPFC | 11 | 0.18 | 0.02 | 0.34 | 0.032 |
| ACC | 9 | 0.36 | 0.10 | 0.61 | 0.006 |
| Temporal lobe | 7 | -0.10 | -0.48 | 0.29 | 0.628 |
| Thalamus | 9 | -0.04 | -0.23 | 0.15 | 0.695 |
| Other frontal lobe | 4 | 0.23 | -0.22 | 0.69 | 0.315 |
| PCC | 2 | -0.01 | -0.35 | 0.33 | 0.943 |
| Occipital lobe | 2 | 0.27 | -0.07 | 0.61 | 0.121 |
| Centrum semiovale | 2 | 0.20 | -0.14 | 0.54 | 0.255 |
| Cerebellum | 2 | 0.26 | -0.44 | 0.95 | 0.465 |
| Parietal lobe | 2 | 1.04 | 0.40 | 1.68 | 0.002 |
| **Medicated** | | | | | |
| **Region** | **Cohorts** | **SMD** | **Lower 95% CI** | **Upper 95% CI** | **P value** |
| ACC | 46 | 0.21 | 0.08 | 0.35 | 0.001 |
| dlPFC | 26 | 0.28 | 0.12 | 0.44 | 0.001 |
| Occipital lobe | 8 | 0.24 | 0.03 | 0.45 | 0.024 |
| Thalamus | 23 | -0.11 | -0.24 | 0.02 | 0.106 |
| Hippocampus | 26 | 0.05 | -0.07 | 0.16 | 0.415 |
| Temporal lobe | 15 | -0.11 | -0.37 | 0.14 | 0.388 |
| mPFC | 12 | 0.20 | 0.01 | 0.38 | 0.039 |
| Other frontal lobe | 11 | 0.27 | 0.02 | 0.51 | 0.037 |
| Parietal lobe | 7 | 0.47 | 0.03 | 0.91 | 0.037 |
| Striatum | 23 | 0.19 | 0.03 | 0.35 | 0.018 |
| Frontal white matter | 18 | 0.03 | -0.15 | 0.21 | 0.752 |
| Centrum semiovale | 4 | 0.23 | -0.01 | 0.47 | 0.064 |
| Cerebellum | 4 | -0.29 | -0.63 | 0.05 | 0.093 |
| Global grey matter | 3 | 0.11 | -0.11 | 0.32 | 0.331 |
| Global white matter | 3 | 0.06 | -0.15 | 0.28 | 0.551 |
| Corpus callosum | 2 | -0.26 | -0.88 | 0.36 | 0.414 |
| PCC | 3 | 0.43 | -0.28 | 1.13 | 0.235 |
| **Medication naïve** | | | | | |
| **Region** | **Cohorts** | **SMD** | **Lower 95% CI** | **Upper 95% CI** | **P value** |
| Hippocampus | 5 | 0.17 | -0.28 | 0.63 | 0.462 |
| dlPFC | 8 | -0.26 | -0.49 | -0.03 | 0.028 |
| Striatum | 10 | 0.48 | 0.19 | 0.77 | 0.001 |
| mPFC | 8 | 0.13 | -0.21 | 0.46 | 0.453 |
| Temporal lobe | 5 | -0.25 | -0.74 | 0.24 | 0.311 |
| Other frontal lobe | 2 | 0.39 | -0.30 | 1.08 | 0.269 |
| ACC | 4 | 0.60 | 0.04 | 1.17 | 0.037 |
| Frontal white matter | 3 | -0.17 | -0.46 | 0.12 | 0.261 |
| Thalamus | 5 | -0.15 | -0.40 | 0.11 | 0.255 |
| Cerebellum | 2 | 0.26 | -0.44 | 0.95 | 0.465 |

**Supplementary table 23: Subgroup analysis of medication naïve, medication free and medicated in psychosis spectrum disorders**

| **Region** | **Cohorts** | **Effect estimate** | **Lower 95% CI** | **Upper 95% CI** | **P value** |
| --- | --- | --- | --- | --- | --- |
| Hippocampus | 39 | 0.019 | 0.008 | 0.031 | 0.001 |
| Frontal white matter | 24 | 0.022 | 0.001 | 0.043 | 0.043 |
| Cerebellum | 7 | 0.041 | -0.014 | 0.095 | 0.142 |
| ACC | 65 | 0.010 | -0.004 | 0.025 | 0.168 |
| Temporal lobe | 22 | 0.015 | -0.014 | 0.044 | 0.313 |
| Occipital lobe | 10 | 0.008 | -0.013 | 0.029 | 0.456 |
| dlPFC | 39 | 0.004 | -0.013 | 0.021 | 0.636 |
| Centrum semiovale | 6 | -0.004 | -0.023 | 0.014 | 0.642 |
| Other frontal lobe | 16 | 0.006 | -0.020 | 0.032 | 0.652 |
| Thalamus | 35 | -0.002 | -0.016 | 0.011 | 0.738 |
| mPFC | 27 | 0.002 | -0.016 | 0.020 | 0.850 |
| Striatum | 36 | 0.001 | -0.014 | 0.017 | 0.869 |
| Parietal lobe | 10 | -0.002 | -0.058 | 0.054 | 0.945 |

**Supplementary table 24: Meta regression of year of publication for SMD in psychosis spectrum disorders**

| **Region** | **Cohorts** | **Effect estimate** | **Lower 95% CI** | **Upper 95% CI** | **P value** |
| --- | --- | --- | --- | --- | --- |
| Hippocampus | 37 | -0.000079 | -0.00018 | 0.000024 | 0.133 |
| Temporal lobe | 22 | 0.00031 | -0.00025 | 0.00087 | 0.279 |
| mPFC | 27 | -0.000066 | -0.00019 | 0.000059 | 0.300 |
| Striatum | 36 | -0.000087 | -0.00029 | 0.00012 | 0.405 |
| ACC | 64 | -0.000026 | -0.000096 | 0.000044 | 0.467 |
| Occipital lobe | 10 | -0.000062 | -0.00028 | 0.00016 | 0.578 |
| Centrum semiovale | 6 | -0.00012 | -0.00056 | 0.00031 | 0.586 |
| Cerebellum | 7 | 0.00032 | -0.00086 | 0.0015 | 0.592 |
| Frontal white matter | 24 | -0.000038 | -0.00019 | 0.00011 | 0.615 |
| Parietal lobe | 10 | -0.00036 | -0.0020 | 0.0012 | 0.657 |
| dlPFC | 39 | 0.00002 | -0.00011 | 0.00015 | 0.765 |
| Thalamus | 35 | 0.000017 | -0.00011 | 0.00014 | 0.785 |
| Other frontal lobe | 16 | -0.000093 | -0.00092 | 0.00073 | 0.824 |

**Supplementary table 25: Meta regression of TR (ms) for SMD in psychosis spectrum disorders**

| **Region** | **Cohorts** | **Effect estimate** | **Lower 95% CI** | **Upper 95% CI** | **P value** |
| --- | --- | --- | --- | --- | --- |
| mPFC | 27 | 0.0016 | 0.00011 | 0.0032 | 0.036 |
| Frontal white matter | 24 | -0.0013 | -0.0027 | 0.00018 | 0.087 |
| Hippocampus | 39 | -0.00081 | -0.0019 | 0.00028 | 0.147 |
| Temporal lobe | 21 | 0.0013 | -0.00090 | 0.0034 | 0.249 |
| Occipital lobe | 10 | 0.00091 | -0.00082 | 0.0026 | 0.305 |
| Other frontal lobe | 15 | 0.00081 | -0.0014 | 0.0030 | 0.467 |
| Parietal lobe | 9 | -0.0020 | -0.0085 | 0.0048 | 0.577 |
| Centrum semiovale | 6 | 0.00042 | -0.0011 | 0.0019 | 0.579 |
| Cerebellum | 7 | -0.0012 | -0.0088 | 0.0052 | 0.712 |
| dlPFC | 39 | -0.00030 | -0.0020 | 0.0014 | 0.733 |
| Thalamus | 35 | 0.00014 | -0.0010 | 0.0013 | 0.814 |
| ACC | 63 | 0.000082 | -0.0013 | 0.0015 | 0.907 |
| Striatum | 35 | 0.0000015 | -0.0017 | 0.0017 | 0.999 |

**Supplementary table 26: Meta regression of TE (ms) for SMD in psychosis spectrum disorders**

| **Region** | **Cohorts** | **Effect estimate** | **Lower 95% CI** | **Upper 95% CI** | **P value** |
| --- | --- | --- | --- | --- | --- |
| dlPFC | 36 | 0.000079 | 0.000052 | 0.00011 | <0.001 |
| Temporal lobe | 18 | 0.000022 | -0.000013 | 0.000058 | 0.214 |
| Thalamus | 30 | -0.000014 | -0.000053 | 0.000025 | 0.478 |
| ACC | 57 | -0.00000056 | -0.000015 | 0.0000097 | 0.677 |
| Cerebellum | 7 | -0.000015 | -0.00011 | 0.000077 | 0.744 |
| Occipital lobe | 9 | -0.0000042 | -0.000037 | 0.000029 | 0.803 |
| Frontal white matter | 23 | 0.00000033 | -0.000025 | 0.000032 | 0.824 |
| Hippocampus | 33 | 0.0000038 | -0.000030 | 0.000038 | 0.828 |
| Centrum semiovale | 6 | -0.0000015 | -0.000017 | 0.000014 | 0.855 |
| Striatum | 34 | 0.0000018 | -0.000019 | 0.000023 | 0.867 |
| mPFC | 23 | -0.0000011 | -0.000018 | 0.000016 | 0.896 |
| Parietal lobe | 7 | 0.000054 | -0.000080 | 0.000091 | 0.902 |
| Other frontal lobe | 13 | -0.00000094 | -0.000025 | 0.000023 | 0.938 |

**Supplementary table 27: Meta regression of ROI size (mm^3^) for SMD in psychosis spectrum disorders**

| **Region** | **Cohorts** | **Effect estimate** | **Lower 95% CI** | **Upper 95% CI** | **P value** |
| --- | --- | --- | --- | --- | --- |
| Thalamus | 10 | -0.011 | -0.028 | 0.007 | 0.241 |
| Striatum | 13 | 0.004 | -0.009 | 0.016 | 0.566 |
| Frontal white matter | 8 | 0.003 | -0.011 | 0.017 | 0.675 |
| mPFC | 6 | 0.004 | -0.034 | 0.042 | 0.841 |
| Hippocampus | 14 | -0.001 | -0.015 | 0.013 | 0.883 |
| Other frontal lobe | 6 | 0.001 | -0.017 | 0.019 | 0.940 |
| dlPFC | 12 | 0.000 | -0.019 | 0.019 | 0.987 |
| Temporal lobe | 5 | 0.000 | -0.017 | 0.017 | 0.988 |
| ACC | 27 | 0.000 | -0.014 | 0.014 | 0.995 |

**Supplementary table 28: Meta regression of antidepressant use percentage for SMD in psychosis spectrum disorders**

| **Motion exclusion applied vs not or not reported** | | | | | | |
| --- | --- | --- | --- | --- | --- | --- |
| **Brain Region** | **Restriction** | **Cohorts** | **SMD** | **Lower 95% CI** | **Upper 95% CI** | **P value** |
| ACC | Motion-excluded | 53 | 0.197 | 0.109 | 0.285 | <0.001 |
|  | Not motion-excluded | 12 | 0.467 | -0.025 | 0.960 | 0.063 |
| Centrum semiovale | Motion-excluded | 6 | 0.176 | -0.008 | 0.359 | 0.061 |
| Cerebellum | Not motion-excluded | 4 | -0.261 | -0.607 | 0.085 | 0.139 |
|  | Motion-excluded | 3 | 0.020 | -0.589 | 0.629 | 0.948 |
| dlPFC | Motion-excluded | 30 | 0.181 | 0.031 | 0.330 | 0.018 |
|  | Not motion-excluded | 9 | 0.036 | -0.303 | 0.374 | 0.836 |
| Frontal white matter | Not motion-excluded | 6 | 0.169 | -0.090 | 0.428 | 0.202 |
|  | Motion-excluded | 18 | -0.006 | -0.179 | 0.167 | 0.943 |
| Hippocampus | Motion-excluded | 28 | 0.088 | -0.022 | 0.198 | 0.115 |
|  | Not motion-excluded | 11 | 0.065 | -0.154 | 0.284 | 0.559 |
| mPFC | Motion-excluded | 25 | 0.131 | 0.023 | 0.239 | 0.018 |
| Occipital lobe | Motion-excluded | 9 | 0.246 | 0.055 | 0.436 | 0.011 |
| Other frontal lobe | Motion-excluded | 7 | 0.403 | 0.134 | 0.672 | 0.003 |
|  | Not motion-excluded | 9 | 0.214 | -0.114 | 0.542 | 0.200 |
| Parietal lobe | Not motion-excluded | 4 | 1.033 | 0.651 | 1.416 | <0.001 |
|  | Motion-excluded | 6 | 0.111 | -0.374 | 0.595 | 0.654 |
| Striatum | Motion-excluded | 30 | 0.271 | 0.116 | 0.427 | 0.001 |
|  | Not motion-excluded | 6 | 0.181 | -0.108 | 0.469 | 0.219 |
| Temporal lobe | Motion-excluded | 11 | -0.145 | -0.317 | 0.027 | 0.099 |
|  | Not motion-excluded | 11 | -0.049 | -0.465 | 0.367 | 0.817 |
| Thalamus | Not motion-excluded | 11 | -0.215 | -0.494 | 0.064 | 0.131 |
|  | Motion-excluded | 24 | -0.072 | -0.186 | 0.041 | 0.211 |
| **Risk of bias low vs moderate or high** | | | | | | |
| **Brain Region** | **Restriction** | **Cohorts** | **SMD** | **Lower 95% CI** | **Upper 95% CI** | **P value** |
| ACC | Moderate-High | 56 | 0.236 | 0.121 | 0.351 | <0.001 |
|  | Low | 9 | 0.180 | -0.056 | 0.417 | 0.135 |
| Centrum semiovale | Moderate-High | 6 | 0.176 | -0.008 | 0.359 | 0.061 |
| Cerebellum | Moderate-High | 7 | -0.128 | -0.410 | 0.153 | 0.371 |
| dlPFC | Moderate-High | 37 | 0.149 | 0.006 | 0.292 | 0.041 |
| Frontal white matter | Moderate-High | 22 | 0.080 | -0.065 | 0.225 | 0.279 |
| Hippocampus | Moderate-High | 36 | 0.072 | -0.030 | 0.173 | 0.168 |
|  | Low | 3 | 0.099 | -0.244 | 0.443 | 0.571 |
| mPFC | Moderate-High | 25 | 0.113 | -0.011 | 0.237 | 0.075 |
| Occipital lobe | Moderate-High | 10 | 0.241 | 0.053 | 0.429 | 0.012 |
| Other frontal lobe | Moderate-High | 14 | 0.227 | 0.011 | 0.443 | 0.040 |
| Parietal lobe | Moderate-High | 10 | 0.457 | 0.038 | 0.877 | 0.033 |
| Striatum | Moderate-High | 35 | 0.227 | 0.101 | 0.353 | <0.001 |
| Temporal lobe | Moderate-High | 22 | -0.109 | -0.324 | 0.107 | 0.322 |
| Thalamus | Moderate-High | 31 | -0.130 | -0.250 | -0.010 | 0.034 |
|  | Low | 4 | 0.039 | -0.200 | 0.278 | 0.749 |

**Supplementary table 29: Restriction-based sensitivity analyses for SMD in psychosis spectrum disorders**

| **Excluding studies at high risk of bias** | | | | | |
| --- | --- | --- | --- | --- | --- |
| **Brain Region** | **Cohorts** | **SMD** | **Lower 95% CI** | **Upper 95% CI** | **P value** |
| ACC | 41 | 0.233 | 0.113 | 0.352 | <0.001 |
| Cerebellum | 5 | -0.091 | -0.468 | 0.285 | 0.635 |
| dlPFC | 25 | 0.136 | -0.035 | 0.308 | 0.119 |
| Frontal white matter | 15 | -0.016 | -0.217 | 0.186 | 0.879 |
| Hippocampus | 25 | 0.083 | -0.033 | 0.200 | 0.162 |
| mPFC | 14 | 0.125 | -0.004 | 0.254 | 0.057 |
| Occipital lobe | 6 | 0.157 | -0.079 | 0.394 | 0.193 |
| Other frontal lobe | 7 | 0.602 | 0.377 | 0.826 | <0.001 |
| Parietal lobe | 6 | 0.489 | -0.006 | 0.985 | 0.053 |
| Striatum | 27 | 0.217 | 0.075 | 0.359 | 0.003 |
| Temporal lobe | 9 | -0.157 | -0.351 | 0.037 | 0.112 |
| Thalamus | 20 | -0.021 | -0.138 | 0.096 | 0.724 |
| **Excluding studies that reported any substance misuse** | | | | | |
| **Brain Region** | **Cohorts** | **SMD** | **Lower 95% CI** | **Upper 95% CI** | **P value** |
| ACC | 57 | 0.217 | 0.105 | 0.329 | <0.001 |
| Centrum semiovale | 5 | 0.215 | -0.017 | 0.447 | 0.070 |
| Cerebellum | 5 | -0.312 | -0.609 | -0.015 | 0.039 |
| dlPFC | 36 | 0.137 | -0.009 | 0.282 | 0.065 |
| Frontal white matter | 24 | 0.033 | -0.111 | 0.177 | 0.653 |
| Hippocampus | 39 | 0.077 | -0.019 | 0.173 | 0.114 |
| mPFC | 22 | 0.118 | -0.029 | 0.266 | 0.116 |
| Occipital lobe | 10 | 0.241 | 0.053 | 0.429 | 0.012 |
| Other frontal lobe | 16 | 0.293 | 0.076 | 0.509 | 0.008 |
| Parietal lobe | 10 | 0.457 | 0.038 | 0.877 | 0.033 |
| Striatum | 30 | 0.267 | 0.122 | 0.413 | <0.001 |
| Temporal lobe | 22 | -0.109 | -0.324 | 0.107 | 0.322 |
| Thalamus | 33 | -0.085 | -0.197 | 0.027 | 0.137 |
| **Excluding studies that did not explicitly report excluding participants taking antidepressants** | | | | | |
| **Brain Region** | **Cohorts** | **SMD** | **Lower 95% CI** | **Upper 95% CI** | **P value** |
| ACC | 6 | 0.146 | -0.223 | 0.515 | 0.439 |
| dlPFC | 6 | 0.032 | -0.260 | 0.323 | 0.832 |
| Hippocampus | 5 | 0.188 | -0.100 | 0.475 | 0.201 |
| **Excluding studies that did not explicitly report excluding participants with psychiatric comorbidities** | | | | | |
| **Brain Region** | **Cohorts** | **SMD** | **Lower 95% CI** | **Upper 95% CI** | **P value** |
| ACC | 13 | 0.302 | 0.052 | 0.552 | 0.018 |
| dlPFC | 17 | -0.022 | -0.206 | 0.163 | 0.819 |
| Frontal white matter | 6 | -0.045 | -0.414 | 0.324 | 0.811 |
| Hippocampus | 10 | 0.022 | -0.165 | 0.209 | 0.818 |
| mPFC | 6 | 0.303 | 0.085 | 0.521 | 0.006 |
| Striatum | 9 | 0.168 | 0.002 | 0.334 | 0.047 |
| Temporal lobe | 7 | -0.179 | -0.487 | 0.129 | 0.254 |
| Thalamus | 8 | -0.023 | -0.266 | 0.220 | 0.853 |

**Supplementary table 30: Exclusion-based sensitivity analyses** **for SMD in psychosis spectrum disorders**

| **Brain Region** | **Cohorts** | **SMD** | **Lower 95% CI** | **Upper 95% CI** | **largest absolute change in the SMD** | **Most influential omission** |
| --- | --- | --- | --- | --- | --- | --- |
| ACC | 65 | 0.226 | 0.123 | 0.328 | 0.019 | Yasukawa 2005 |
| Centrum semiovale | 6 | 0.176 | -0.008 | 0.359 | 0.039 | Wang 2019 |
| Cerebellum | 7 | -0.128 | -0.410 | 0.153 | 0.133 | de la Fuente Sandoval 2013 |
| dlPFC | 39 | 0.148 | 0.011 | 0.284 | 0.031 | Smucny 2022 |
| Frontal white matter | 24 | 0.033 | -0.111 | 0.177 | 0.040 | Ota 2012 |
| Hippocampus | 39 | 0.077 | -0.019 | 0.173 | 0.022 | Callicott 1998 |
| mPFC | 27 | 0.121 | 0.009 | 0.234 | 0.019 | Wijtenburg 2017 |
| Occipital lobe | 10 | 0.241 | 0.053 | 0.429 | 0.060 | Smucny 2022 |
| Other frontal lobe | 16 | 0.293 | 0.076 | 0.509 | 0.072 | Omori 2000 |
| Parietal lobe | 10 | 0.457 | 0.038 | 0.877 | 0.126 | Ongur 2010 |
| Striatum | 36 | 0.252 | 0.120 | 0.384 | 0.030 | de la Fuente Sandoval 2013 |
| Temporal lobe | 22 | -0.109 | -0.324 | 0.107 | 0.050 | Yasukawa 2005 |
| Thalamus | 35 | -0.103 | -0.211 | 0.005 | 0.026 | Wang 2019 |

**Supplementary table 31: Leave one out analyses** **for SMD in psychosis spectrum disorders**

| **Brain Region** | **Subgroup** | **Cohorts** | **SMD** | **Lower 95% CI** | **Upper 95% CI** | **P value for SMD** | **QM statistic** | **P value for QM statistic** |
| --- | --- | --- | --- | --- | --- | --- | --- | --- |
| ACC | Non-Siemens | 43 | 0.251 | 0.096 | 0.407 | 0.002 | 0.033 | 0.855 |
|  | Siemens | 21 | 0.230 | 0.117 | 0.342 | <0.001 |  |  |
| Centrum semiovale | Non-Siemens | 6 | 0.176 | -0.008 | 0.359 | 0.061 | NA | NA |
| Cerebellum | Non-Siemens | 5 | -0.112 | -0.503 | 0.279 | 0.576 | NA | NA |
| dlPFC | Non-Siemens | 28 | 0.106 | -0.052 | 0.263 | 0.188 | 0.977 | 0.323 |
|  | Siemens | 11 | 0.262 | -0.007 | 0.531 | 0.056 |  |  |
| Frontal white matter | Non-Siemens | 18 | -0.008 | -0.165 | 0.150 | 0.924 | 0.659 | 0.417 |
|  | Siemens | 6 | 0.133 | -0.183 | 0.450 | 0.409 |  |  |
| Hippocampus | Non-Siemens | 27 | 0.080 | -0.046 | 0.205 | 0.212 | 0.000 | 0.990 |
|  | Siemens | 12 | 0.075 | -0.086 | 0.237 | 0.361 |  |  |
| mPFC | Non-Siemens | 16 | 0.117 | -0.060 | 0.293 | 0.195 | 0.024 | 0.877 |
|  | Siemens | 11 | 0.128 | -0.035 | 0.291 | 0.123 |  |  |
| Occipital lobe | Non-Siemens | 9 | 0.181 | -0.029 | 0.390 | 0.091 | NA | NA |
| Other frontal lobe | Non-Siemens | 14 | 0.227 | 0.011 | 0.443 | 0.040 | NA | NA |
| Parietal lobe | Non-Siemens | 8 | 0.581 | 0.128 | 1.034 | 0.012 | NA | NA |
| Striatum | Non-Siemens | 30 | 0.273 | 0.132 | 0.415 | <0.001 | 0.606 | 0.436 |
|  | Siemens | 6 | 0.129 | -0.269 | 0.527 | 0.525 |  |  |
| Temporal lobe | Non-Siemens | 17 | -0.129 | -0.384 | 0.127 | 0.323 | 0.120 | 0.729 |
|  | Siemens | 5 | -0.036 | -0.465 | 0.394 | 0.870 |  |  |
| Thalamus | Non-Siemens | 21 | -0.142 | -0.295 | 0.010 | 0.068 | 0.644 | 0.422 |
|  | Siemens | 13 | -0.028 | -0.192 | 0.135 | 0.733 |  |  |

**Supplementary table 32: Subgroup analyses for MRI machine vendor**

| **Brain Region** | **Subgroup** | **Cohorts** | **SMD** | **Lower 95% CI** | **Upper 95% CI** | **P value for SMD** | **QM statistic** | **P value for QM statistic** |
| --- | --- | --- | --- | --- | --- | --- | --- | --- |
| ACC | Non-PRESS | 20 | 0.255 | 0.111 | 0.399 | 0.001 | 0.133 | 0.715 |
|  | PRESS | 41 | 0.222 | 0.092 | 0.352 | 0.001 |  |  |
| Centrum semiovale | Non-PRESS | 5 | 0.174 | -0.019 | 0.366 | 0.077 | NA | NA |
| Cerebellum | PRESS | 6 | -0.128 | -0.453 | 0.197 | 0.441 | NA | NA |
| dlPFC | Non-PRESS | 16 | -0.007 | -0.246 | 0.232 | 0.955 | 2.123 | 0.145 |
|  | PRESS | 18 | 0.188 | 0.026 | 0.350 | 0.023 |  |  |
| Frontal white matter | Non-PRESS | 9 | 0.041 | -0.206 | 0.288 | 0.743 | 0.001 | 0.975 |
|  | PRESS | 14 | 0.045 | -0.142 | 0.232 | 0.638 |  |  |
| Hippocampus | Non-PRESS | 10 | -0.076 | -0.328 | 0.175 | 0.552 | 5.231 | 0.022 |
|  | PRESS | 25 | 0.141 | 0.026 | 0.256 | 0.016 |  |  |
| mPFC | Non-PRESS | 14 | 0.073 | -0.050 | 0.196 | 0.246 | 1.400 | 0.237 |
|  | PRESS | 11 | 0.260 | 0.012 | 0.508 | 0.040 |  |  |
| Occipital lobe | Non-PRESS | 7 | 0.296 | 0.062 | 0.530 | 0.013 | 0.709 | 0.400 |
|  | PRESS | 3 | 0.014 | -0.570 | 0.598 | 0.962 |  |  |
| Other frontal lobe | Non-PRESS | 7 | 0.255 | -0.074 | 0.585 | 0.129 | 0.177 | 0.674 |
|  | PRESS | 8 | 0.346 | 0.020 | 0.672 | 0.037 |  |  |
| Parietal lobe | Non-PRESS | 4 | 0.370 | -0.483 | 1.224 | 0.395 | 0.132 | 0.716 |
|  | PRESS | 6 | 0.520 | 0.039 | 1.000 | 0.034 |  |  |
| Striatum | Non-PRESS | 13 | 0.155 | -0.012 | 0.321 | 0.068 | 0.445 | 0.505 |
|  | PRESS | 20 | 0.300 | 0.077 | 0.522 | 0.008 |  |  |
| Temporal lobe | Non-PRESS | 9 | 0.125 | -0.155 | 0.405 | 0.382 | 4.423 | 0.035 |
|  | PRESS | 13 | -0.305 | -0.600 | -0.010 | 0.042 |  |  |
| Thalamus | Non-PRESS | 16 | -0.095 | -0.257 | 0.068 | 0.255 | 0.016 | 0.899 |
|  | PRESS | 16 | -0.114 | -0.302 | 0.074 | 0.233 |  |  |

**Supplementary table 33: Subgroup analyses for acquisition sequence**

| **Creatine correction only vs other correction methods** | | | | | | | | |
| --- | --- | --- | --- | --- | --- | --- | --- | --- |
| **Brain Region** | **Restriction** | **Cohorts** | **SMD** | **Lower 95% CI** | **Upper 95% CI** | **P value for SMD** | **QM statistic** | **P value for QM statistic** |
| ACC | Other | 36 | 0.207 | 0.096 | 0.317 | <0.001 | 0.151 | 0.698 |
|  | Creatine | 29 | 0.259 | 0.075 | 0.443 | 0.006 |  |  |
| Centrum semiovale | Creatine | 3 | 0.243 | -0.061 | 0.547 | 0.117 | 0.297 | 0.585 |
|  | Other | 3 | 0.137 | -0.093 | 0.367 | 0.244 |  |  |
| Cerebellum | Creatine | 3 | -0.277 | -0.681 | 0.127 | 0.179 | 0.581 | 0.446 |
|  | Other | 4 | -0.037 | -0.488 | 0.414 | 0.873 |  |  |
| dlPFC | Other | 21 | 0.187 | 0.045 | 0.330 | 0.010 | 0.754 | 0.385 |
|  | Creatine | 18 | 0.089 | -0.156 | 0.333 | 0.476 |  |  |
| Frontal white matter | Other | 13 | 0.065 | -0.146 | 0.276 | 0.548 | 0.195 | 0.659 |
|  | Creatine | 11 | -0.004 | -0.201 | 0.192 | 0.965 |  |  |
| Hippocampus | Other | 14 | 0.106 | -0.044 | 0.256 | 0.165 | 0.267 | 0.605 |
|  | Creatine | 25 | 0.057 | -0.070 | 0.185 | 0.379 |  |  |
| mPFC | Creatine | 13 | 0.238 | 0.015 | 0.462 | 0.037 | 1.992 | 0.158 |
|  | Other | 14 | 0.050 | -0.074 | 0.174 | 0.430 |  |  |
| Occipital lobe | Creatine | 5 | 0.360 | 0.117 | 0.603 | 0.004 | 2.314 | 0.128 |
|  | Other | 5 | 0.063 | -0.245 | 0.370 | 0.689 |  |  |
| Other frontal lobe | Other | 4 | 0.586 | 0.156 | 1.016 | 0.008 | 2.327 | 0.127 |
|  | Creatine | 12 | 0.205 | -0.035 | 0.445 | 0.094 |  |  |
| Parietal lobe | Other | 7 | 0.479 | 0.063 | 0.896 | 0.024 | 0.026 | 0.872 |
|  | Creatine | 3 | 0.435 | -0.765 | 1.634 | 0.477 |  |  |
| Striatum | Other | 17 | 0.453 | 0.195 | 0.711 | 0.001 | 4.790 | 0.029 |
|  | Creatine | 19 | 0.126 | -0.014 | 0.265 | 0.078 |  |  |
| Temporal lobe | Creatine | 20 | -0.143 | -0.378 | 0.092 | 0.232 | 0.854 | 0.355 |
| Thalamus | Other | 15 | -0.139 | -0.311 | 0.032 | 0.111 | 0.337 | 0.561 |
|  | Creatine | 20 | -0.073 | -0.211 | 0.064 | 0.297 |  |  |
| **Field strength** | | | | | | | | |
| **Brain Region** | **Restriction** | **Cohorts** | **SMD** | **Lower 95% CI** | **Upper 95% CI** | **P value** | **QM statistic** | **P value for QM statistic** |
| ACC | >=3T | 48 | 0.204 | 0.102 | 0.306 | <0.001 | 0.454 | 0.501 |
|  | <3T | 17 | 0.312 | 0.010 | 0.613 | 0.043 |  |  |
| Centrum semiovale | <3T | 3 | 0.243 | -0.061 | 0.547 | 0.117 | 0.297 | 0.585 |
|  | >=3T | 3 | 0.137 | -0.093 | 0.367 | 0.244 |  |  |
| Cerebellum | <3T | 4 | -0.317 | -0.636 | 0.002 | 0.051 | 3.153 | 0.076 |
|  | >=3T | 3 | 0.114 | -0.418 | 0.646 | 0.675 |  |  |
| dlPFC | >=3T | 14 | 0.265 | -0.020 | 0.551 | 0.069 | 1.392 | 0.238 |
|  | <3T | 25 | 0.048 | -0.080 | 0.175 | 0.466 |  |  |
| Frontal white matter | <3T | 18 | 0.040 | -0.136 | 0.216 | 0.656 | 0.018 | 0.893 |
|  | >=3T | 6 | 0.016 | -0.247 | 0.280 | 0.903 |  |  |
| Hippocampus | >=3T | 17 | 0.152 | 0.028 | 0.276 | 0.016 | 3.257 | 0.071 |
|  | <3T | 22 | -0.002 | -0.156 | 0.152 | 0.982 |  |  |
| mPFC | <3T | 10 | 0.219 | -0.102 | 0.539 | 0.181 | 1.189 | 0.276 |
|  | >=3T | 17 | 0.070 | -0.041 | 0.181 | 0.215 |  |  |
| Occipital lobe | >=3T | 3 | 0.348 | 0.038 | 0.657 | 0.028 | 0.722 | 0.395 |
|  | <3T | 7 | 0.178 | -0.061 | 0.417 | 0.144 |  |  |
| Other frontal lobe | <3T | 14 | 0.225 | 0.005 | 0.445 | 0.045 | 3.026 | 0.082 |
| Parietal lobe | <3T | 7 | 0.594 | 0.108 | 1.080 | 0.017 | 0.947 | 0.330 |
|  | >=3T | 3 | 0.146 | -0.693 | 0.984 | 0.734 |  |  |
| Striatum | >=3T | 18 | 0.261 | 0.095 | 0.427 | 0.002 | 0.055 | 0.815 |
|  | <3T | 18 | 0.244 | 0.030 | 0.459 | 0.025 |  |  |
| Temporal lobe | <3T | 21 | -0.136 | -0.356 | 0.084 | 0.226 | 1.131 | 0.288 |
| Thalamus | >=3T | 11 | -0.140 | -0.283 | 0.003 | 0.055 | 0.225 | 0.635 |
|  | <3T | 24 | -0.091 | -0.251 | 0.069 | 0.265 |  |  |

**Supplementary table 34: Subgroup analyses for field strength and creatine correction**

| **PANSS Total score** | | | | | |
| --- | --- | --- | --- | --- | --- |
| **Region** | **Cohorts** | **Effect estimate** | **Lower 95% CI** | **Upper 95% CI** | **P value** |
| ACC | 22 | 0.0034 | -0.0159 | 0.0227 | 0.7271 |
| dlPFC | 12 | -0.0239 | -0.0492 | 0.0013 | 0.0635 |
| Frontal white matter | 7 | -0.0061 | -0.0203 | 0.0081 | 0.3992 |
| Hippocampus | 12 | 0.0050 | -0.0020 | 0.0120 | 0.1578 |
| mPFC | 7 | 0.0076 | -0.0013 | 0.0165 | 0.0944 |
| Striatum | 13 | 0.0039 | -0.0058 | 0.0136 | 0.4272 |
| Thalamus | 12 | 0.0111 | -0.0012 | 0.0233 | 0.0760 |
| **PANSS Positive score** | | | | | |
| **Region** | **Cohorts** | **Effect estimate** | **Lower 95% CI** | **Upper 95% CI** | **P value** |
| ACC | 25 | -0.0522 | -0.1043 | -0.0002 | 0.0492 |
| dlPFC | 16 | -0.0515 | -0.1058 | 0.0028 | 0.0632 |
| Frontal white matter | 13 | -0.0208 | -0.0531 | 0.0114 | 0.2048 |
| Hippocampus | 14 | 0.0118 | -0.0177 | 0.0413 | 0.4324 |
| mPFC | 13 | 0.0194 | -0.0062 | 0.0450 | 0.1370 |
| Striatum | 18 | 0.0207 | -0.0230 | 0.0645 | 0.3532 |
| Thalamus | 9 | 0.0466 | 0.0083 | 0.0850 | 0.0171 |
| **PANSS Negative score** | | | | | |
| **Region** | **Cohorts** | **Effect estimate** | **Lower 95% CI** | **Upper 95% CI** | **P value** |
| ACC | 42 | 0.8307 | 0.1621 | 1.4994 | 0.0149 |
| dlPFC | 22 | 0.7507 | -0.3781 | 1.8795 | 0.1924 |
| Frontal white matter | 16 | -0.5176 | -1.3613 | 0.3261 | 0.2292 |
| Hippocampus | 23 | -0.2085 | -1.2804 | 0.8634 | 0.7031 |
| mPFC | 17 | 0.6558 | -1.1739 | 2.4854 | 0.4824 |
| Occipital lobe | 8 | -0.3141 | -1.2014 | 0.5732 | 0.4878 |
| Other frontal lobe | 7 | -0.7220 | -1.5531 | 0.1090 | 0.0886 |
| Parietal lobe | 5 | 1.1589 | -1.1554 | 3.4733 | 0.3264 |
| Striatum | 27 | 0.9027 | -0.1159 | 1.9213 | 0.0824 |
| Temporal lobe | 7 | -0.6761 | -1.4441 | 0.0918 | 0.0844 |
| Thalamus | 15 | 0.2857 | -0.4625 | 1.0339 | 0.4542 |

**Supplementary table 35: Meta regression of symptoms scores for SMD in psychosis spectrum disorders**

| **Region** | **Cohorts** | **Effect estimate** | **Lower 95% CI** | **Upper 95% CI** | **P value** |
| --- | --- | --- | --- | --- | --- |
| ACC | 8 | -0.0394 | -0.0599 | -0.0188 | 0.0002 |
| dlPFC | 7 | 0.0075 | -0.0296 | 0.0447 | 0.6914 |
| mPFC | 9 | 0.0115 | -0.0199 | 0.0430 | 0.4729 |
| Striatum | 5 | -0.0362 | -0.0742 | 0.0017 | 0.0612 |

**Supplementary table 36: Meta regression of IQ difference between cases and controls for SMD in psychosis spectrum disorders**

| **Study** | **Case definition** | **Case representativeness** | **Control selection** | **Control definition** | **Case-control comparability** | **Exposure ascertainment** | **Ascertainment method, cases and controls** | **Overall** |
| --- | --- | --- | --- | --- | --- | --- | --- | --- |
| Hardy 2011 | Low | Moderate | Moderate | Low | High | Moderate | Low | High |
| Modinos 2017 | Low | Moderate | Low | Low | Low | Moderate | Low | Moderate |
| Reid 2019 | Low | Moderate | Low | High | Low | Moderate | Low | Moderate |
| Rowland 2016 | Low | Moderate | Low | Low | Low | Moderate | Low | Moderate |
| Smucny 2022 | Low | Moderate | High | High | Low | High | High | High |
| Liu 2015 | Moderate | High | Moderate | High | High | Moderate | High | High |
| Lutkenhoff 2010 | Low | Low | High | High | Low | Moderate | Low | Moderate |
| MacKinley 2022 | Low | Moderate | Low | Low | Low | Moderate | Low | Moderate |
| Marsman 2014 | Low | High | High | High | Low | Moderate | Low | High |
| Martinez-Granados 2008 | Low | High | High | High | Low | Moderate | High | High |
| Merritt 2019 | Low | Moderate | High | High | High | Moderate | High | High |
| Meyer 2016 | Low | Moderate | Moderate | High | Low | High | High | High |
| Miyaoka 2005 | Low | Moderate | Moderate | Low | Low | Low | High | Moderate |
| Molina 2005 | Low | Low | High | High | Low | Moderate | High | High |
| Molina 2007 | Low | High | High | High | Low | Moderate | High | High |
| Moore 2002 | Low | Low | Low | Low | High | Moderate | Low | Moderate |
| Natsubori 2014 | Low | Moderate | High | High | Low | Moderate | High | High |
| O'Neill 2004 | Low | High | Low | Low | Low | Moderate | Low | Moderate |
| Ohara 2000 | Low | Moderate | High | High | High | Moderate | High | High |
| Ohrmann 2008 | Low | Moderate | Low | Low | High | Moderate | High | High |
| Ohrmann 2007 | Low | Moderate | High | Low | High | High | High | High |
| van Elst 2005 | Low | Moderate | Moderate | Low | High | Moderate | High | High |
| Omori 2000 | Low | Moderate | Moderate | High | Low | Moderate | High | High |
| Ongur 2010 | Low | Moderate | High | High | High | Moderate | High | High |
| Ota 2012 | Low | Moderate | Low | Low | Low | Moderate | Low | Moderate |
| Ota 2015 | Low | Moderate | Low | Low | Low | Moderate | Low | Moderate |
| Ozcelik 2020 | Low | Moderate | High | High | High | Moderate | Low | High |
| Pae 2004 | Low | Moderate | High | Low | High | Moderate | Low | High |
| Plitman 2018 | Low | Low | High | Low | Low | Moderate | Low | Moderate |
| Plitman 2016 | Low | Low | High | Low | Low | Moderate | Low | Moderate |
| Reid 2013 | Low | Moderate | Moderate | Low | Low | Moderate | Low | Moderate |
| Reid 2010 | Low | Moderate | Moderate | High | Low | Moderate | High | High |
| Reid 2016 | Low | Low | Moderate | Low | Low | Moderate | High | Moderate |
| Rowland 2013 | Low | High | High | Low | Low | Moderate | Low | Moderate |
| Rowland 2009 | Low | High | High | Low | Low | Moderate | Low | Moderate |
| Sarramea-Crespo 2008 | Low | High | High | High | Low | Moderate | High | High |
| Seese 2011 | Low | High | Low | Low | Low | Moderate | Low | Moderate |
| Shakory 2018 | Low | High | High | Low | Low | Moderate | Low | Moderate |
| Sharma 1992 | Low | Moderate | Moderate | Low | High | Moderate | High | High |
| Shiori 1996 | Low | Moderate | Moderate | High | Low | High | High | High |
| Shirayama 2010 | Low | Moderate | High | High | Low | High | High | High |
| Sigmundsson 2003 | Low | Moderate | Low | Low | Low | Low | Low | Low |
| Singh 2018 | Low | Moderate | Low | Low | Low | Moderate | Low | Moderate |
| Sivaraman 2017 | Low | Low | Low | Low | Low | Moderate | Low | Low |
| Smesny 2022 | Low | Low | Low | High | Low | High | Low | Moderate |
| Stanley 1995 | Low | Moderate | Low | Low | Low | Moderate | Low | Moderate |
| Stone 2009 | Low | Low | Low | Low | High | Moderate | Low | Moderate |
| Szulc 2011 | Low | Low | Low | Low | High | Low | High | Moderate |
| Tanaka 2006 | Low | Moderate | High | High | Low | Low | Low | Moderate |
| Tarumi 2020 | Low | Low | High | Low | Low | Low | High | Moderate |
| Tayoshi 2009 | Low | Moderate | Low | Low | High | Low | Low | Moderate |
| Tibbo 2000 | Low | Moderate | High | Low | High | High | High | High |
| Tibbo 2013 | Low | Low | High | Low | Low | Low | High | Moderate |
| Uhl 2011 | Low | Moderate | Low | Low | Low | Moderate | Low | Moderate |
| Vingerhoets 2019 | Low | Moderate | Low | Low | Low | Moderate | Low | Moderate |
| Wang 2019 | Low | High | High | High | High | Moderate | High | High |
| Wang 2022 | Low | Moderate | Low | Low | High | Moderate | Low | Moderate |
| Wijtenburg 2021 | Low | Moderate | High | High | High | Moderate | Low | High |
| Wijtenburg 2017 | Low | High | High | High | Low | Moderate | Low | High |
| Wood 2003 | Low | Moderate | Low | Low | High | Moderate | High | High |
| Yamasue 2003 | Low | Moderate | High | Low | Low | Moderate | High | High |
| Yamasue 2002 | Low | Moderate | High | Low | Low | Moderate | Low | Moderate |
| Yasukawa 2005 | Low | Moderate | High | Low | Low | Moderate | High | High |
| Zabala 2007 | Low | Low | Low | Low | Low | Moderate | Low | Low |
| Ongur 2008 | Low | Moderate | High | Low | High | Moderate | Low | High |
| Aoyama 2011 | Low | Low | Low | High | Low | Moderate | High | Moderate |
| Birur 2020 | Low | Moderate | High | Low | Low | Low | Low | Moderate |
| Bluml 1999 | Low | High | High | Low | High | Low | Low | Moderate |
| Bossong 2019 | Low | Low | Low | Low | High | Low | Low | Low |
| Bustillo 2011 | Low | Low | High | Low | Low | Moderate | Low | Moderate |
| Da Silva 2019 | Low | High | High | Low | High | Low | High | High |
| Deicken 2001 | Low | High | High | Low | High | Moderate | High | High |
| Deicken 1999 | Low | Moderate | High | Low | High | Moderate | Low | High |
| Demjaha 2014 | Low | High | High | Low | Low | High | High | High |
| Egerton 2018 | Low | Low | High | Low | High | Low | High | Moderate |
| Gan 2017 | Low | Low | Low | Low | Low | Moderate | Low | Low |
| Mcilwain 2015 | Low | Moderate | Low | Low | High | Moderate | Low | Moderate |
| Kragulijac 2013 | Low | Low | High | Low | Low | Low | Low | Low |
| Larabi 2015 | Low | Moderate | Moderate | Low | Low | Moderate | Low | Moderate |
| Lim 1998 | Low | Moderate | Low | High | High | Moderate | High | High |
| Auer 2001 | Low | High | Low | Low | Low | Moderate | High | Moderate |
| Aydin 2008 | Low | High | High | Low | Low | Moderate | Low | Moderate |
| Bartolomeo 2019 | Low | Moderate | Low | Low | Low | Low | Low | Low |
| Bertolinion 1998 | Low | Low | Low | High | Low | High | High | Moderate |
| Bertolinio 1996 | Low | Moderate | Moderate | High | Low | Moderate | High | High |
| Blasi 2004 | Low | High | High | High | Low | Moderate | High | High |
| Block 2000 | Low | Low | Low | High | Low | Moderate | Low | Moderate |
| Brandt 2016 | Low | Moderate | Low | Low | Low | Moderate | High | Moderate |
| Brooks 1998 | Low | Low | Low | Low | Low | Moderate | Low | Low |
| Bustillo 2014 | Low | Moderate | High | Low | Low | Moderate | Low | Moderate |
| Bustillo 2017 | Low | Moderate | High | Low | Low | Moderate | Low | Moderate |
| Bustillo 2019 | Low | Moderate | High | Low | Low | Moderate | Low | Moderate |
| Bustillo 2001 | Low | Moderate | High | Low | High | Moderate | Low | High |
| Bustillo 2002a | Low | Moderate | Low | Low | High | Moderate | Low | Moderate |
| Bustillo 2008 | Low | Moderate | Low | Low | High | Moderate | Low | Moderate |
| Bustillo 2002b | Low | Moderate | High | Low | High | Moderate | Low | High |
| Bustillo 2010 | Low | Moderate | Low | Low | High | Moderate | Low | Moderate |
| Byun 2009 | Low | Moderate | High | Low | Low | Low | Low | Moderate |
| Callicott 1998 | Low | Moderate | Low | Low | High | Moderate | Low | Moderate |
| Cecil 1999 | Low | Low | Low | Low | Low | High | High | Moderate |
| Chang 2007 | Low | Moderate | Low | Low | Low | Moderate | Low | Moderate |
| Chiu 2018 | Low | Moderate | Low | Low | Low | Moderate | Low | Moderate |
| Crocker 2014 | Low | Moderate | Low | Low | High | Moderate | Low | Moderate |
| de la Fuente Sandoval 2013 | Low | Low | High | Low | High | Moderate | Low | Moderate |
| de la Fuente Sandoval 2011 | Low | Moderate | High | Low | Low | Moderate | Low | Moderate |
| de la Fuente Sandoval 2017 | Low | Moderate | High | Low | Low | Moderate | Low | Moderate |
| de la Fuente Sandoval 2015 | Low | Moderate | High | Low | Low | Moderate | Low | Moderate |
| Deicken 2000 | Low | High | High | Low | High | Moderate | Low | High |
| Deicken 1997a | Low | High | High | Low | High | Moderate | Low | High |
| Deicken 1998 | Low | High | High | Low | Low | Moderate | Low | Moderate |
| Deicken 1997b | Low | High | High | Low | High | Moderate | Low | High |
| Delamillieure 2000a | High | High | High | Low | Low | High | High | High |
| Delamillieure 2002 | Low | High | Low | Low | Low | Moderate | High | Moderate |
| Delamillieure 2000b | Low | High | High | Low | High | Moderate | High | High |
| Ende 2001 | High | High | High | Low | High | High | High | High |
| Fannon 2003 | Low | High | High | Low | Low | Moderate | Low | Moderate |
| Fujimoto 1996 | Low | High | High | High | High | High | High | High |
| Fukuzako 2000 | High | Moderate | Moderate | Low | Low | High | High | High |
| Fukuzako 1995 | Low | High | Moderate | Low | Low | High | High | High |
| Galinska 2009 | Low | Moderate | High | High | Low | High | High | High |
| Galinska-Skok 2018 | Low | Moderate | High | High | Low | High | High | High |
| Gallinat 2016 | Low | High | Low | Low | Low | Low | High | Moderate |
| Gan 2014 | Low | Low | Low | Low | Low | Low | High | Low |
| Granata 2013 | Low | High | High | Low | Low | Moderate | Low | Moderate |
| Hagino | Low | Moderate | Low | Low | Low | Low | Low | Low |
| Hasan 2014 | Low | High | Low | Low | High | Low | Low | Moderate |
| He 2018 | Low | Moderate | High | Low | High | Moderate | Low | High |
| He 2012 | Low | Moderate | Low | Low | Low | Moderate | Low | Moderate |
| Heimberg 1998 | Low | Low | Moderate | Low | High | High | High | High |
| Huang 2019 | Low | Moderate | Low | High | High | Low | Low | Moderate |
| Huang 2017 | Low | Moderate | Low | Low | Low | Moderate | Low | Moderate |
| Iwata 2019 | Low | Moderate | High | Low | Low | Low | Low | Moderate |
| Jakary 2005 | Low | Low | High | Low | High | Moderate | Low | Moderate |
| Jessen 2013 | Low | Moderate | Low | Low | High | Low | Low | Moderate |
| Jessen 2006 | Moderate | Low | Low | Low | High | Moderate | High | High |
| Kegeles 2000 | Low | High | High | High | Low | Low | High | High |
| Kim 2018 | Low | High | High | High | Low | High | High | High |
| Kirtas 2016 | Low | Moderate | Moderate | Low | Low | Moderate | Low | Moderate |
| Klar 2010 | Low | Moderate | High | Low | High | Moderate | Low | High |
| Kragulijac 2019 | Low | Low | Low | Low | Low | Low | High | Low |
| Lebedeva 2014 | Low | Moderate | High | Low | High | Low | High | High |
| Legind 2019 | Low | Low | Low | Low | Low | Moderate | Low | Low |
| Bartha 1997 | Low | Moderate | Low | Low | Low | Moderate | Low | Moderate |
| Basoglu 2006 | Low | Moderate | High | Low | High | Low | Low | Moderate |
| Chiappelli 2015 | Low | Moderate | Low | Low | Low | Low | Low | Low |
| Choe 1994 | Low | High | High | High | High | Moderate | High | High |
| Maier 2000 | Low | High | High | High | High | High | High | High |
| Premkumar 2010 | Low | Moderate | Low | Low | Low | Low | Low | Low |
| Stanley 2007 | Low | Moderate | Low | Low | High | Moderate | High | High |
| Taylor 2017 | Low | Moderate | High | Low | Low | Moderate | Low | Moderate |
| Terpstra 2005 | Low | Moderate | High | High | High | Moderate | High | High |
| Theberge 2007 | Low | Moderate | Low | Low | High | Moderate | High | High |
| Tunc-Skarka 2009 | Low | Moderate | High | High | High | Low | High | High |
| Venkatraman 2006 | High | High | High | High | Low | High | High | High |
| Ueno 2022 | Low | Low | Low | Low | Low | Moderate | Low | Low |
| Cadenhead 2024 | Low | Low | Low | Low | High | Moderate | Low | Moderate |
| Chiappelli 2024 | Low | Low | Low | Low | Low | Moderate | Low | Low |
| Fan 2024 | Low | Low | Low | Low | High | Low | Low | Low |
| Koster 2024 | Low | Low | Low | Low | Low | Moderate | Low | Low |
| Leon-Ortiz 2023 | High | Low | High | Low | Low | High | High | High |
| Wang 2023 | Low | Low | High | Low | High | Moderate | High | High |
| Allam 2024 | Low | High | High | Low | Low | Moderate | High | High |
| Kubota 2023 | Low | High | Low | Low | Low | Moderate | Low | Moderate |
| Stanley 2023 | Low | Moderate | High | Low | Low | Moderate | Low | Moderate |
| Olbrich 2008 | Low | Moderate | Low | High | Low | Moderate | Low | Moderate |

**Supplementary table 37: Risk of bias findings for individual studies**

**
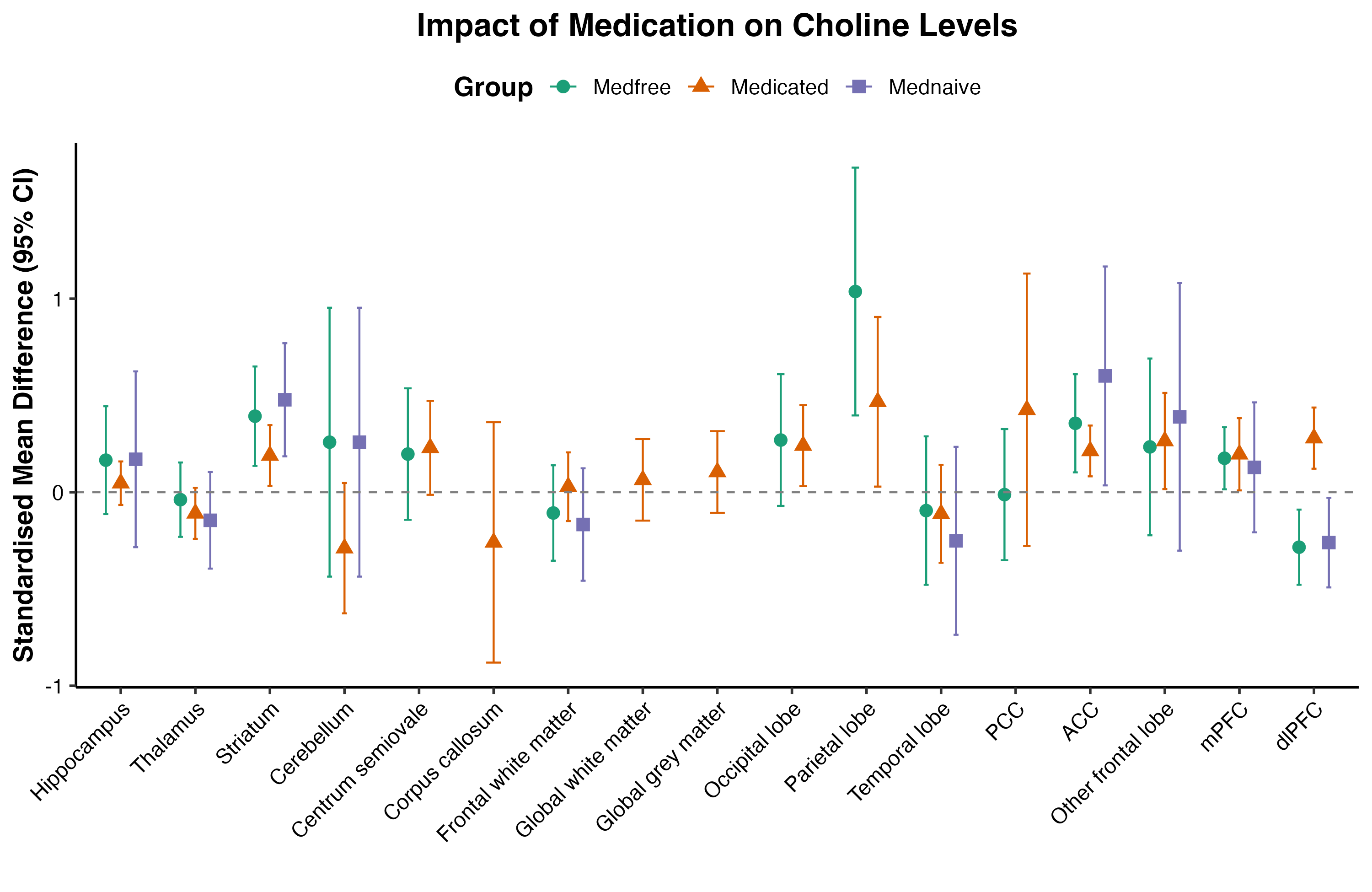
**

**Supplementary figure 1: Impact of medication status on SMD findings between psychosis cases and controls.** Negative SMD indicates lower choline levels in cases, positive SMD indicates higher choline levels in cases. dlPFC indicates dorsolateral prefrontal cortex; mPFC, medial prefrontal cortex; ACC, anterior cingulate cortex; PCC, posterior cingulate cortex

**
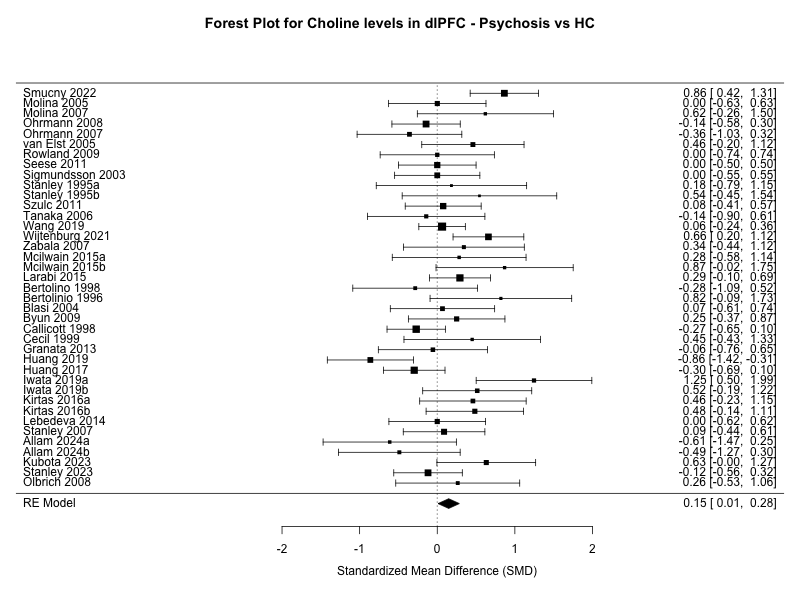
**

**
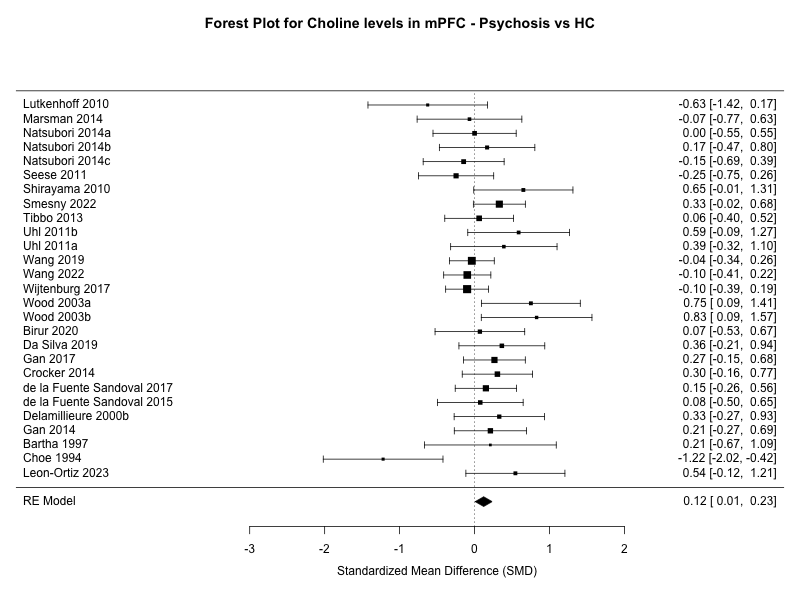
**


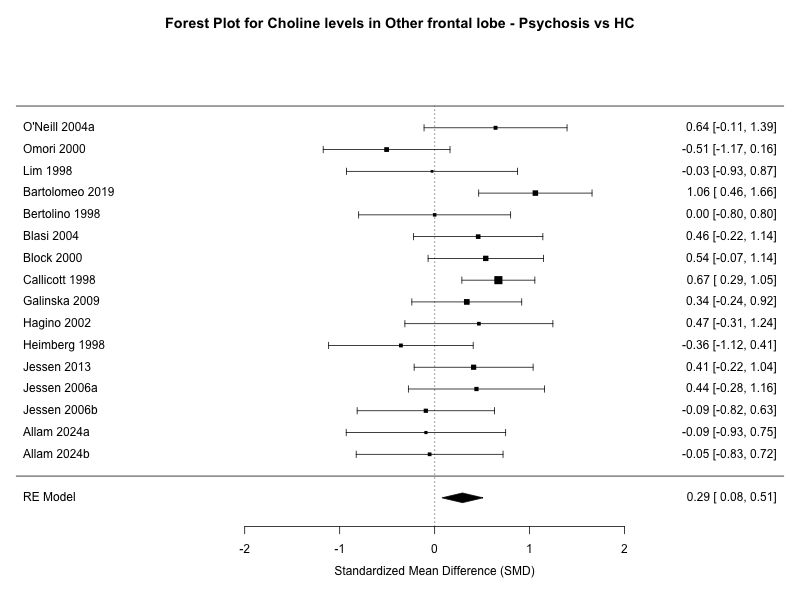


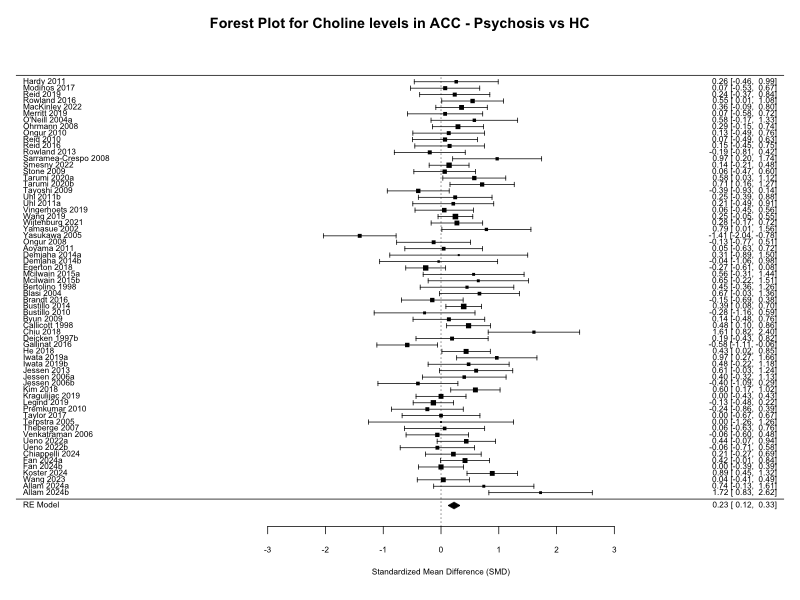


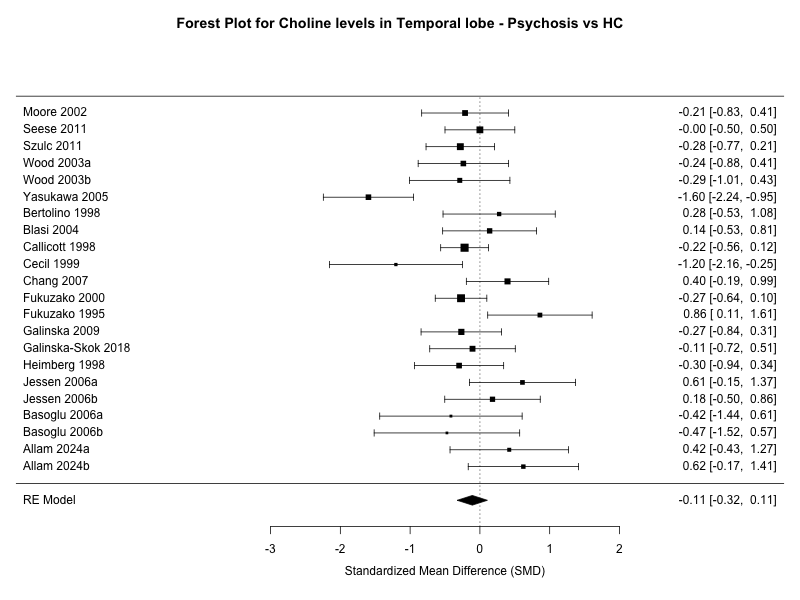


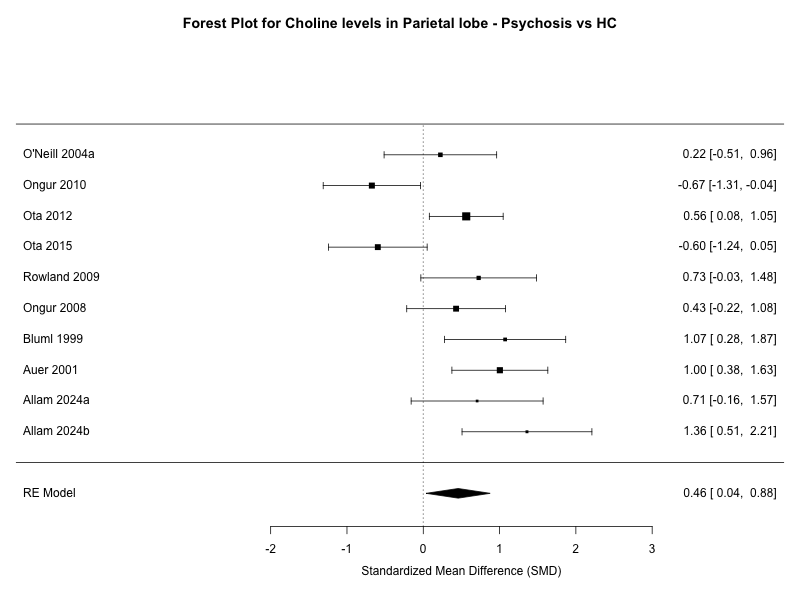


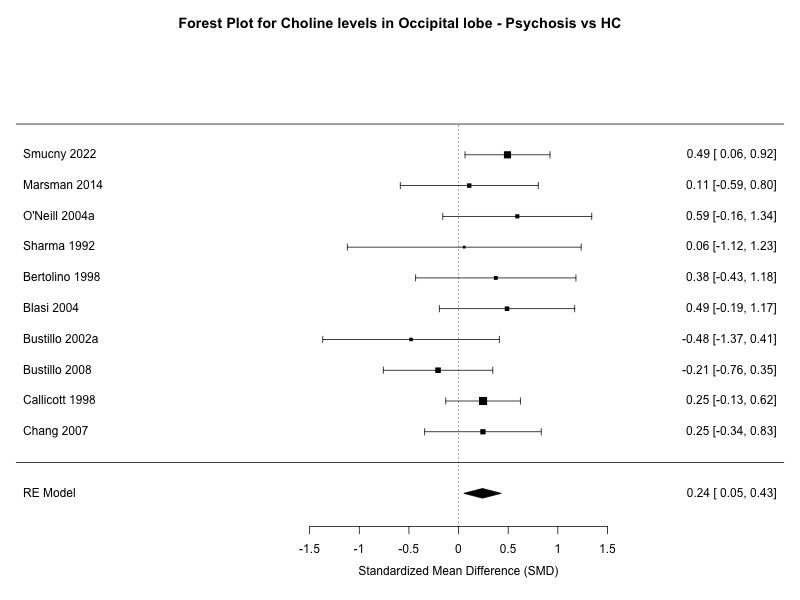


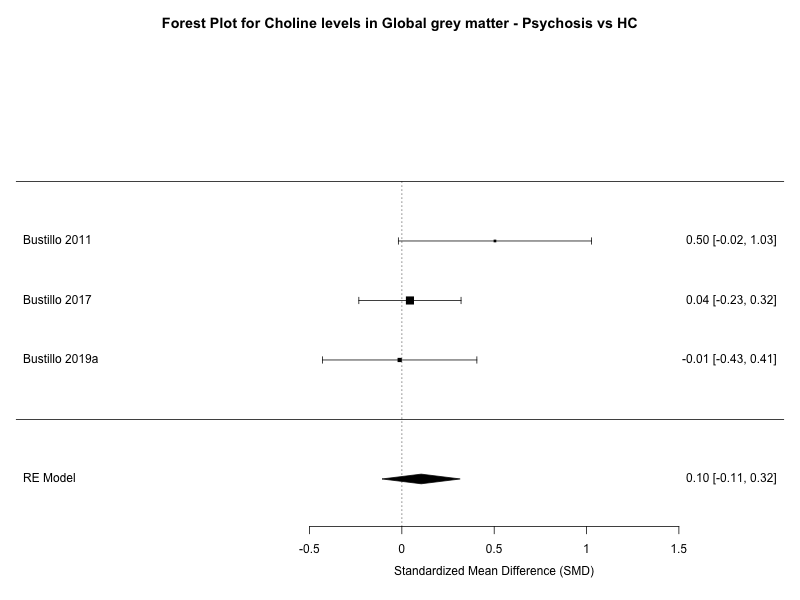


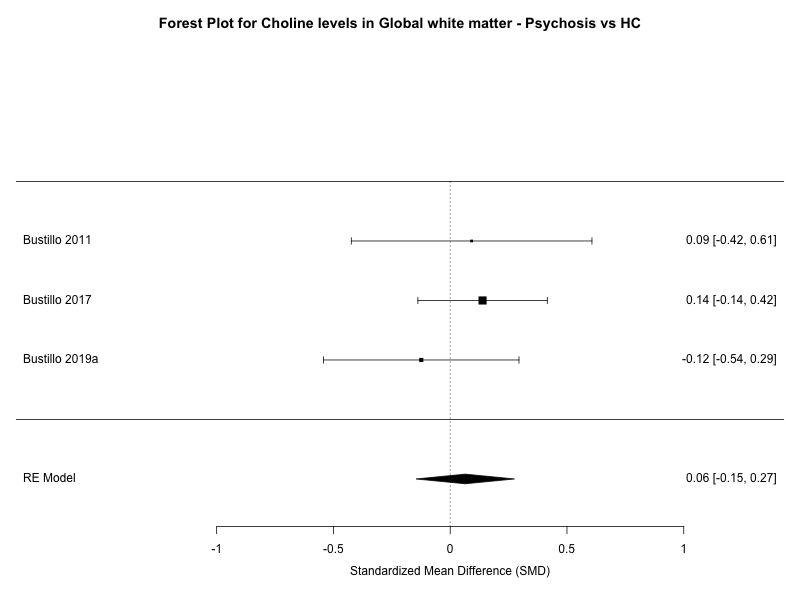


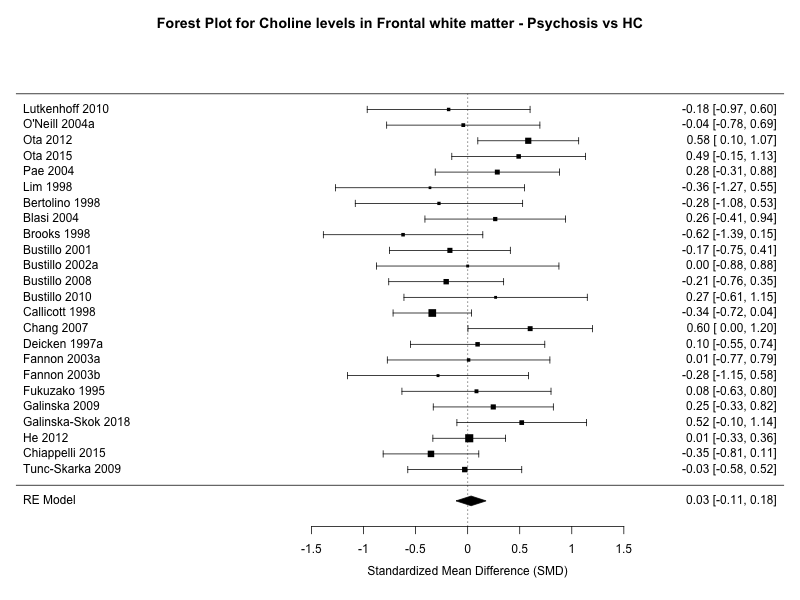


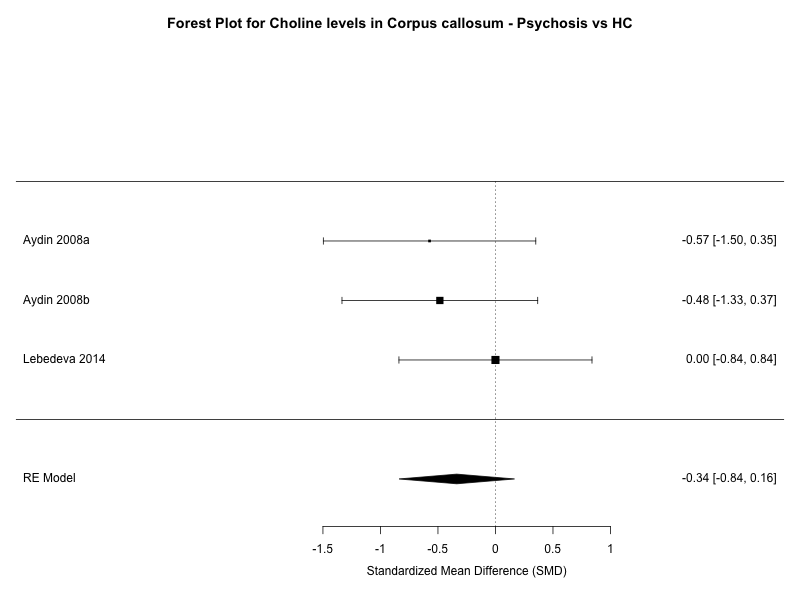


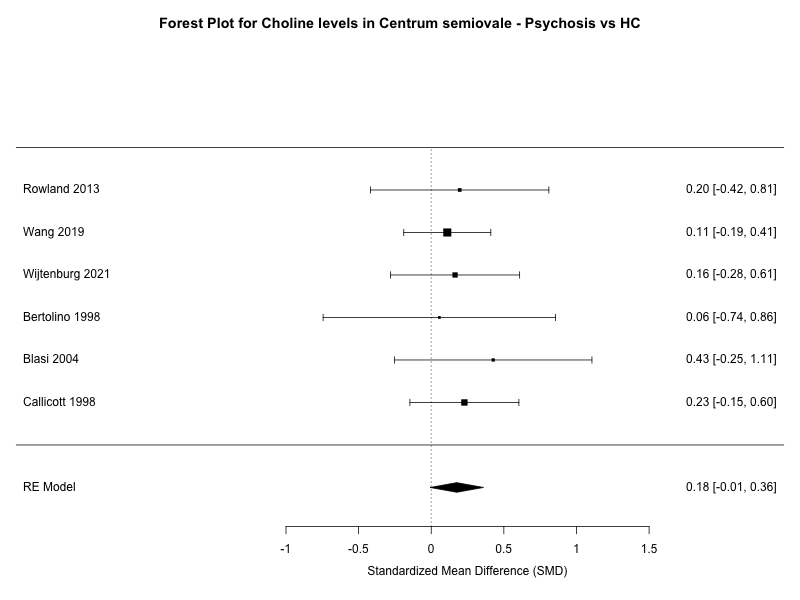


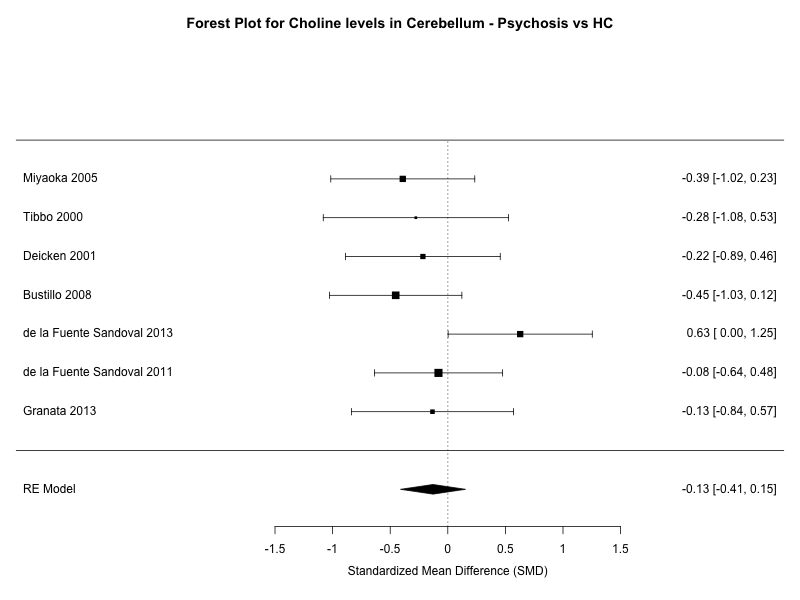


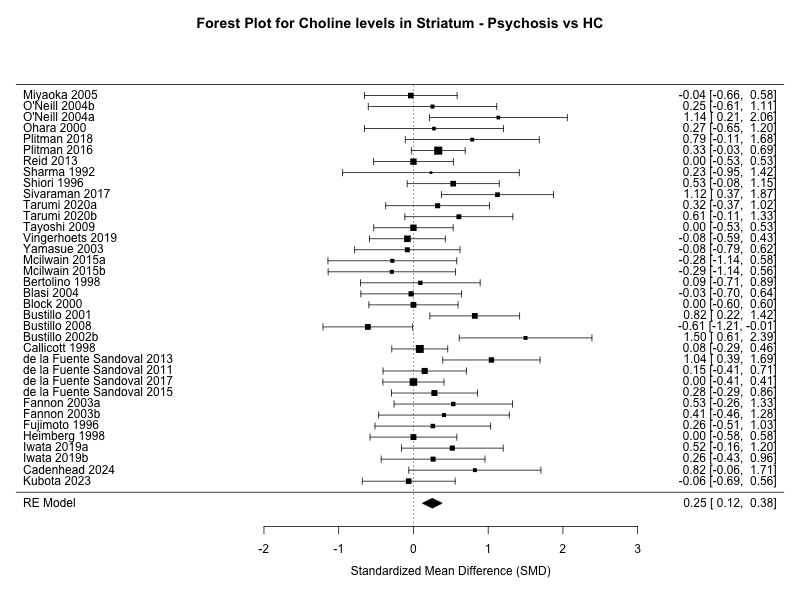


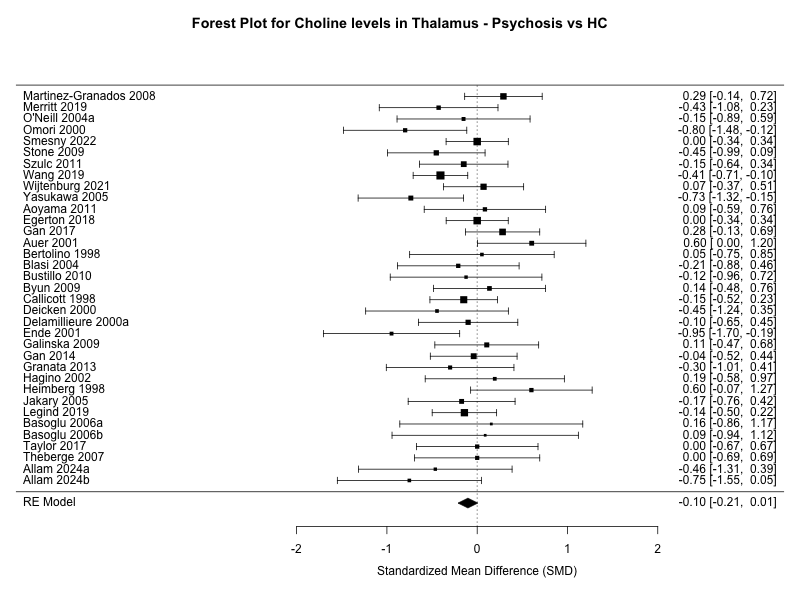


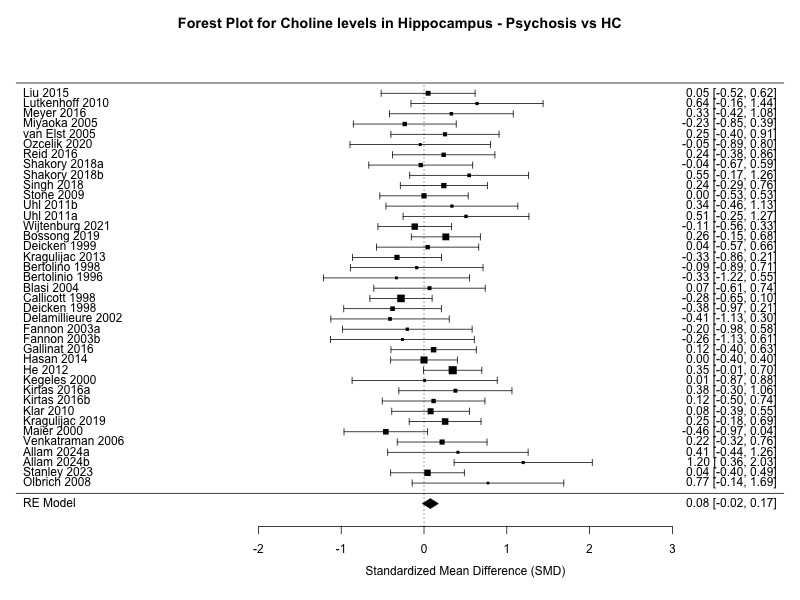


**Supplementary Figures 2A-P: Forest plots of standardised mean differences (SMD) between choline levels in psychosis spectrum disorders and healthy controls.** (A) dlPFC (B) mPFC (C) Other frontal lobe (D) ACC (E) Temporal Lobe (F) Parietal lobe (G) Occipital lobe (H) Global grey matter (I) Global white matter (J) Corpus Callosum (L) Centrum semiovale (M) Cerebellum (N) Striatum (O) Thalamus (P) Hippocampus. Negative SMD indicates lower choline levels in cases, positive SMD indicates higher choline levels in cases. dlPFC indicates dorsolateral prefrontal cortex; mPFC, medial prefrontal cortex; ACC, anterior cingulate cortex; PCC, posterior cingulate cortex; HC, healthy controls.


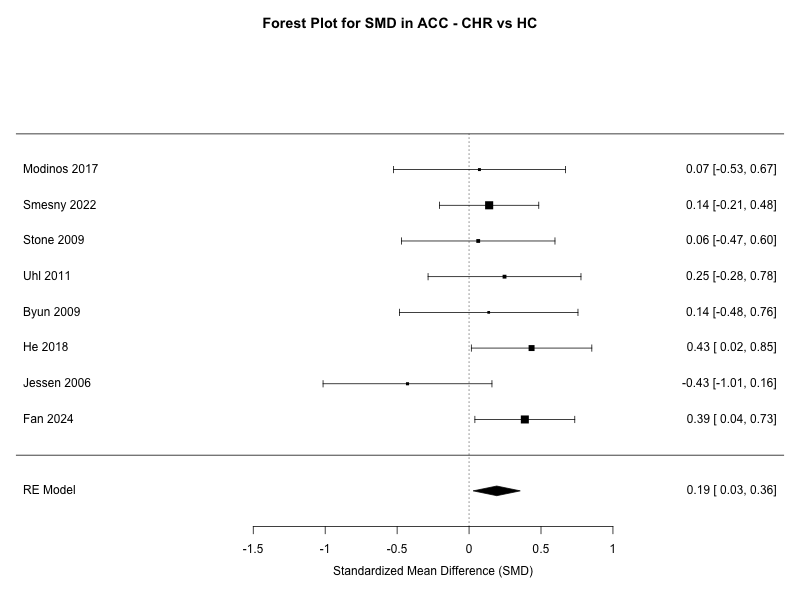


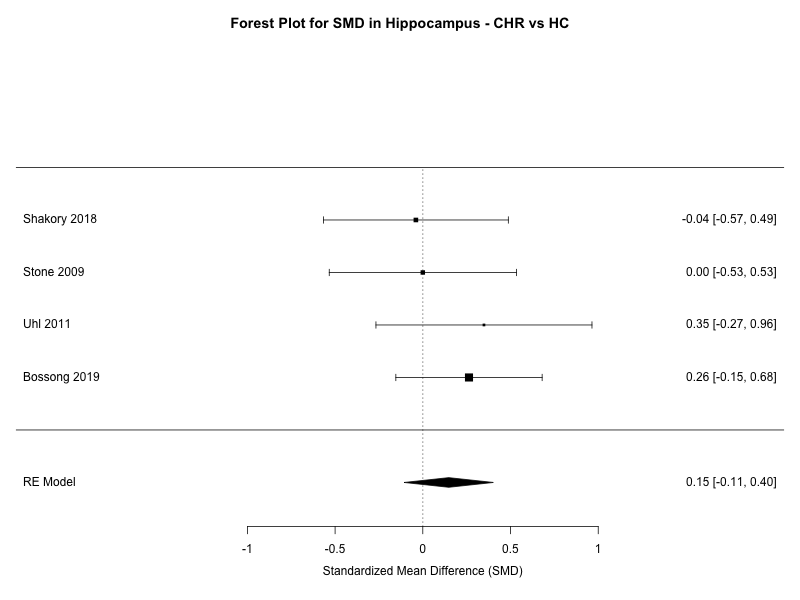


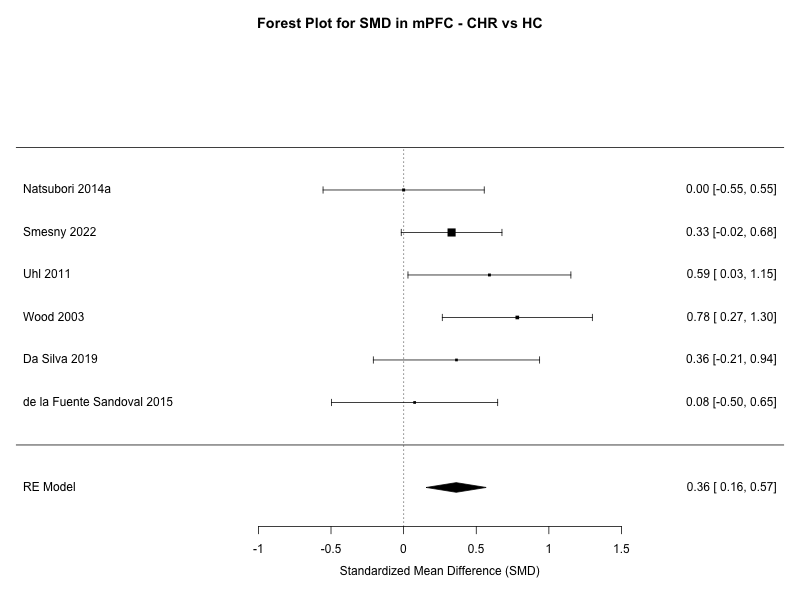


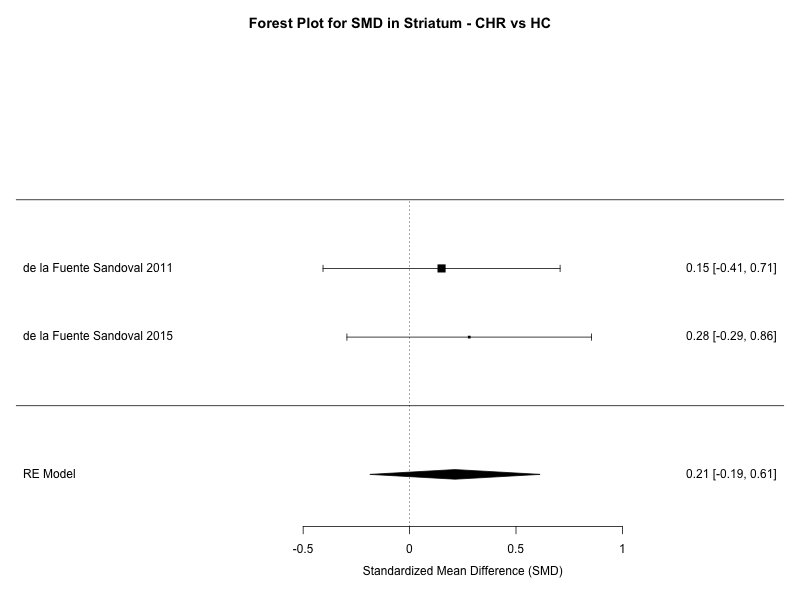


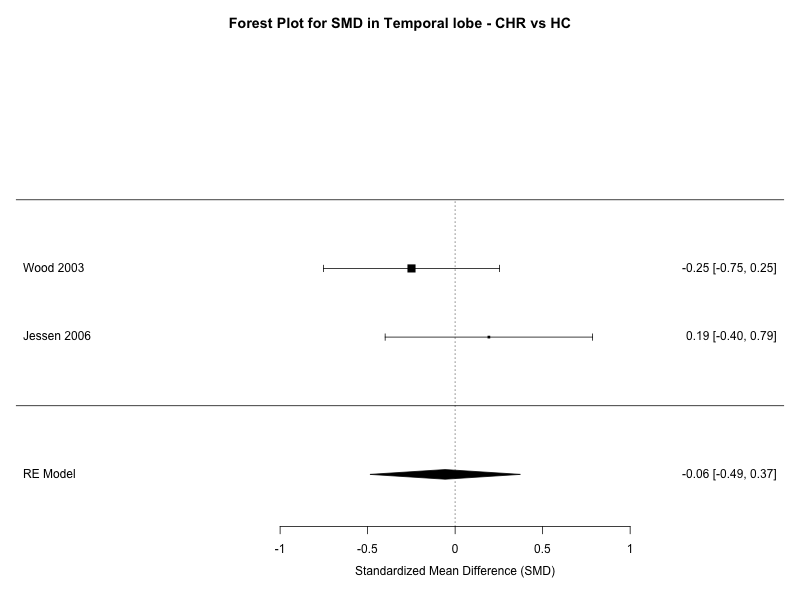


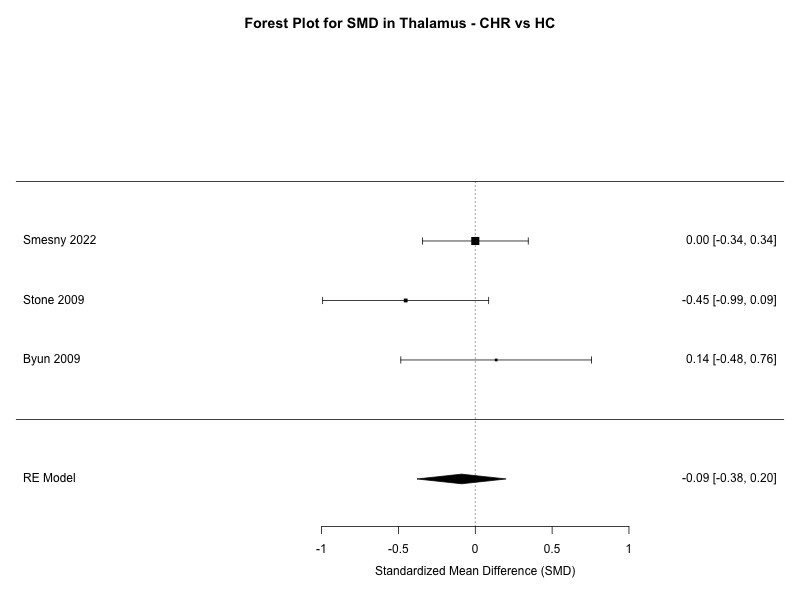


**Supplementary Figures 3A-F: Forest plots of standardised mean differences (SMD) between choline levels in clinical high risk individuals and healthy controls.** (A) ACC (B) Hippocampus (C) mPFC (D) Thalamus (E) Striatum (F) Temporal Lobe. Negative SMD indicates lower choline levels in cases, positive SMD indicates higher choline levels in cases. mPFC, medial prefrontal cortex; ACC, anterior cingulate cortex; PCC, posterior cingulate cortex; HC, healthy controls.


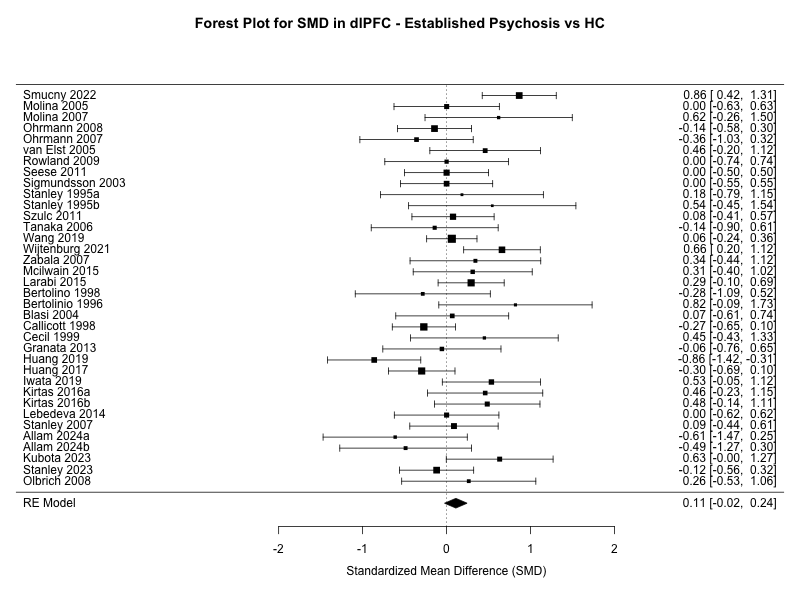


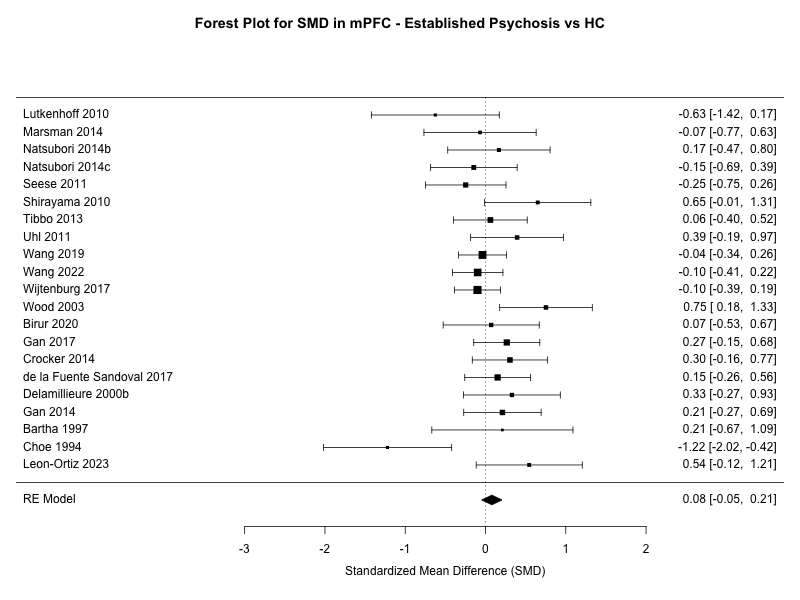


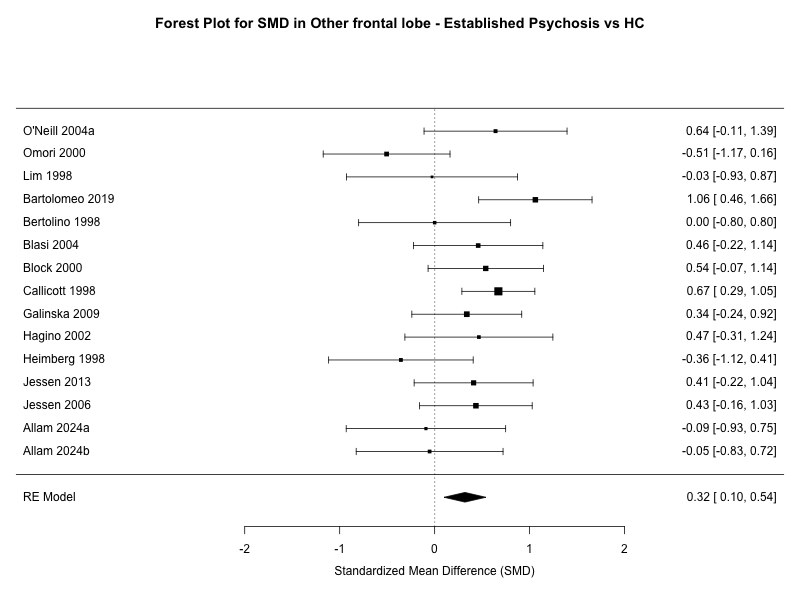


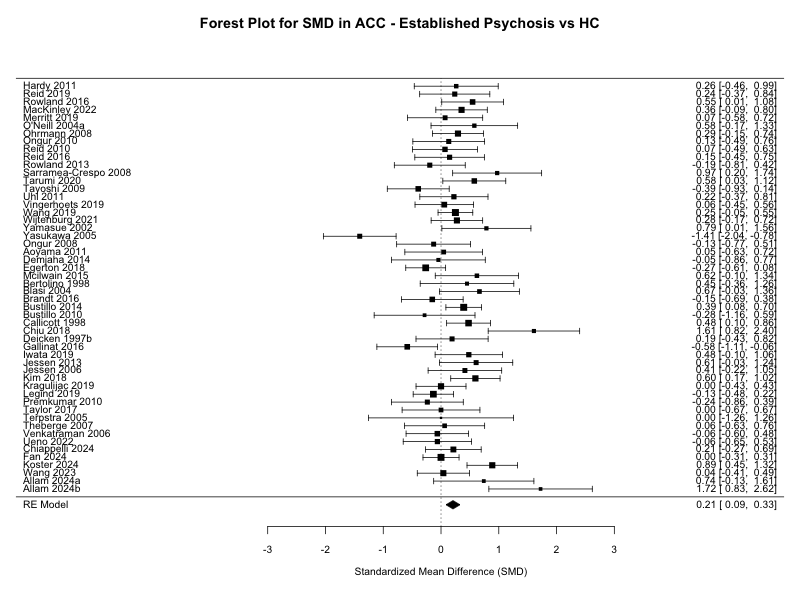


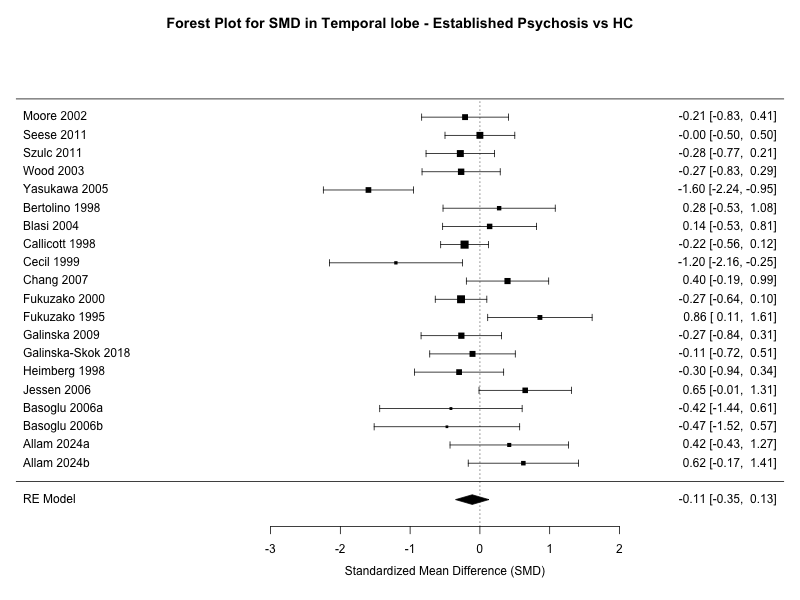


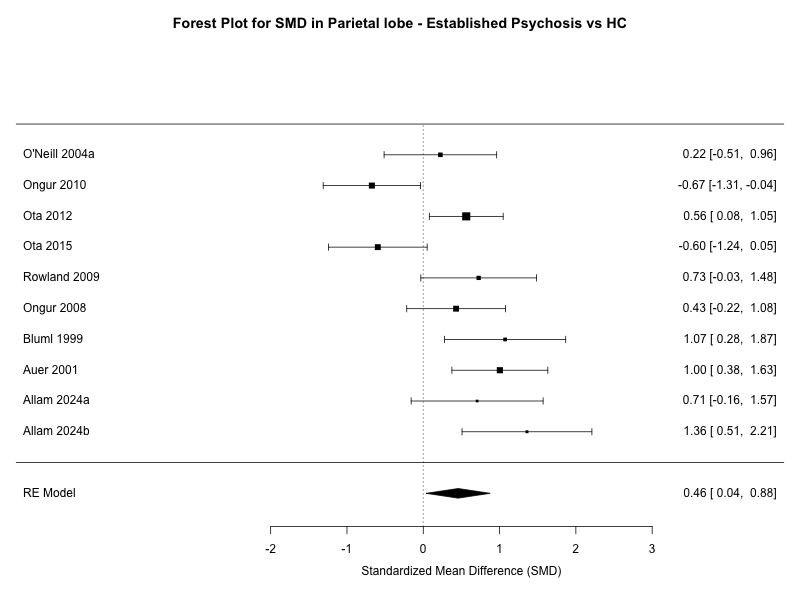


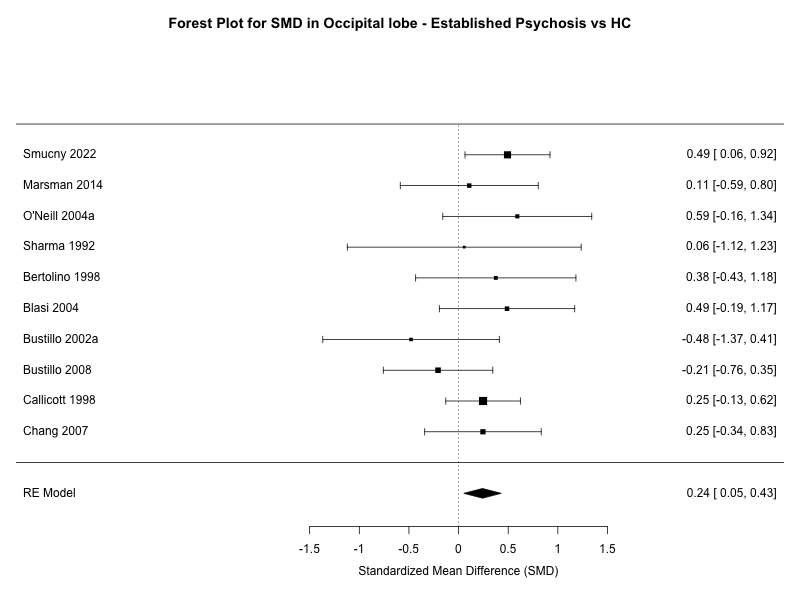


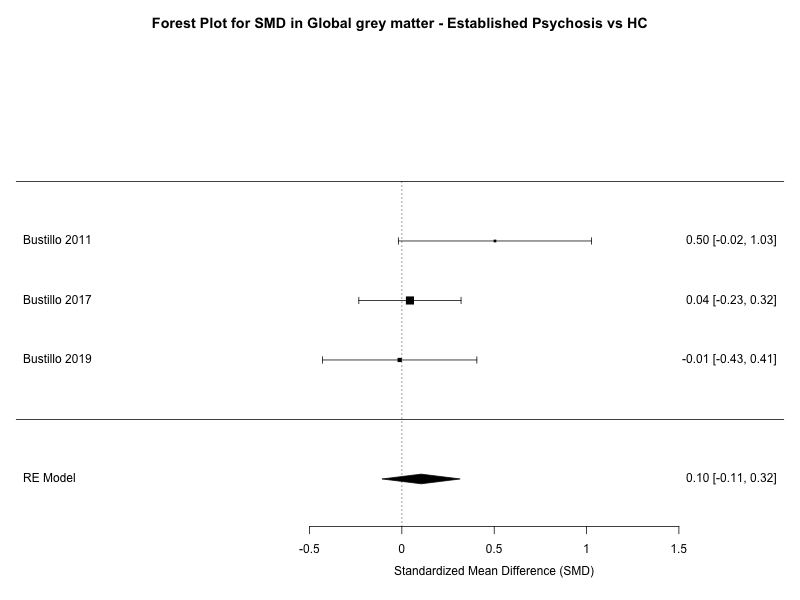


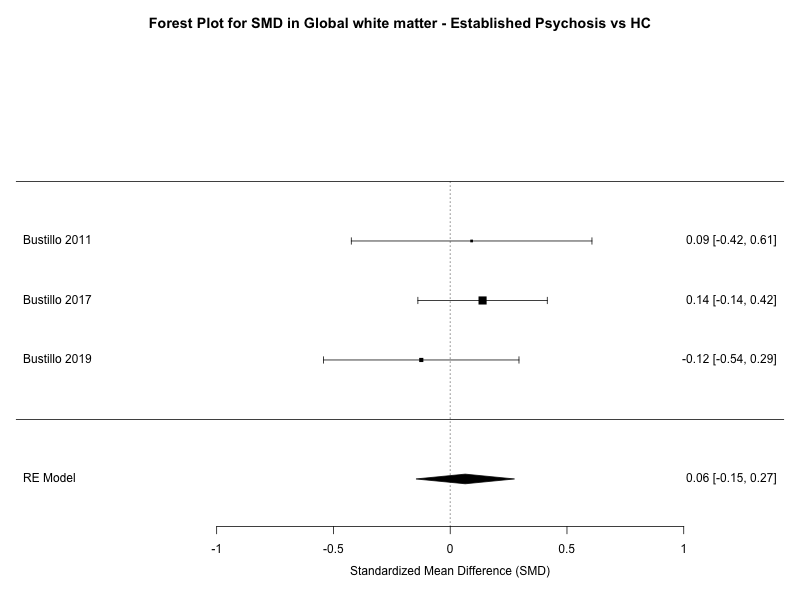


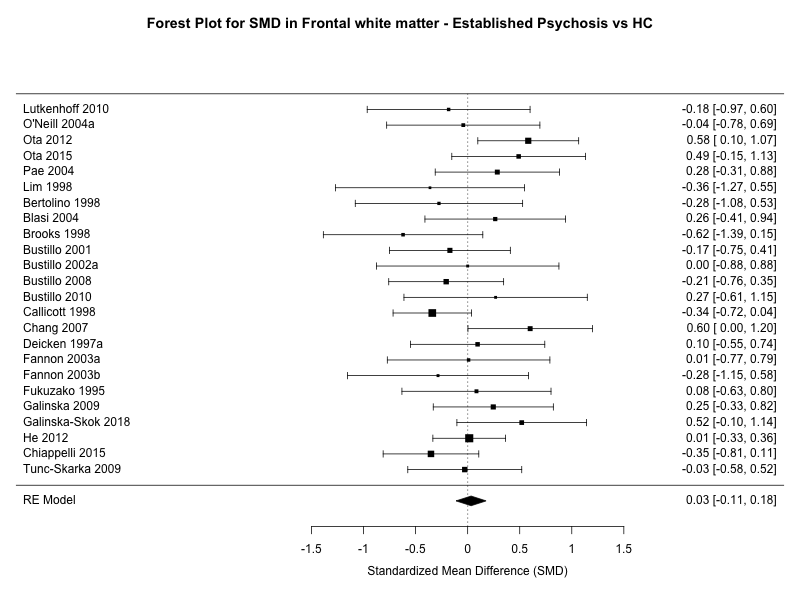


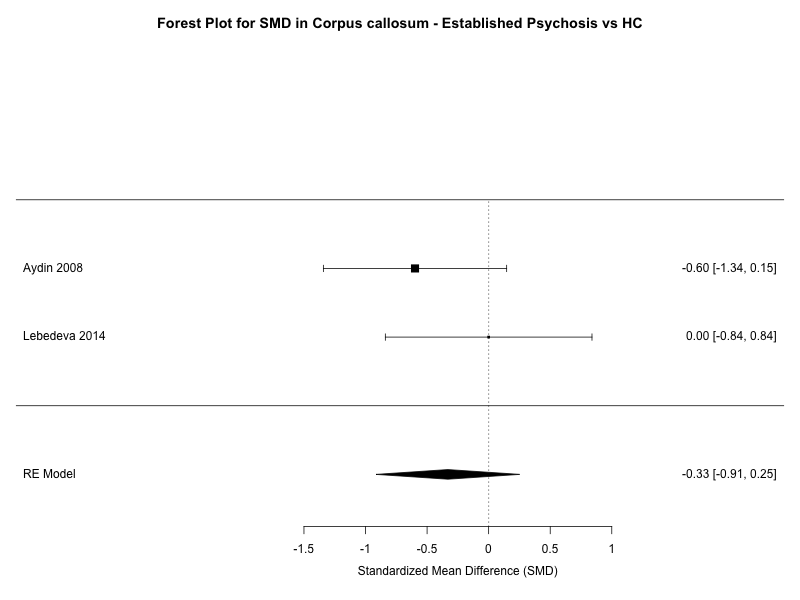


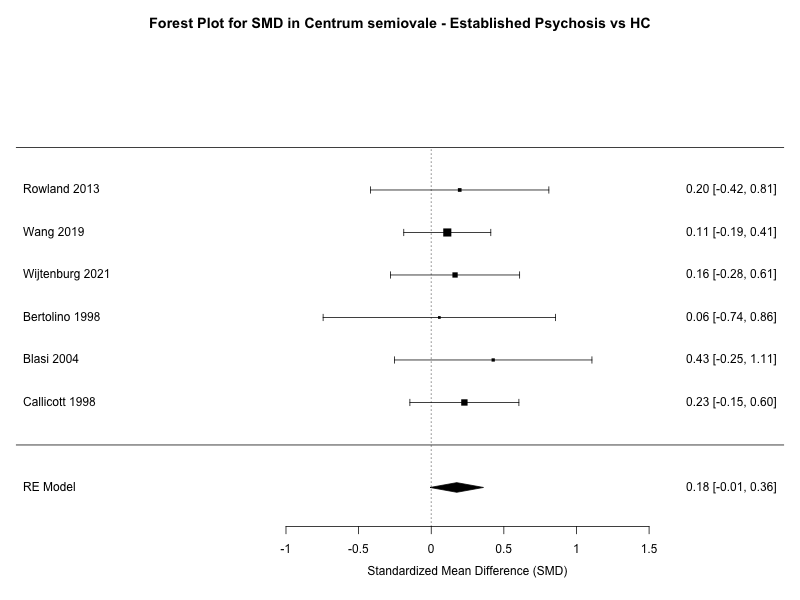


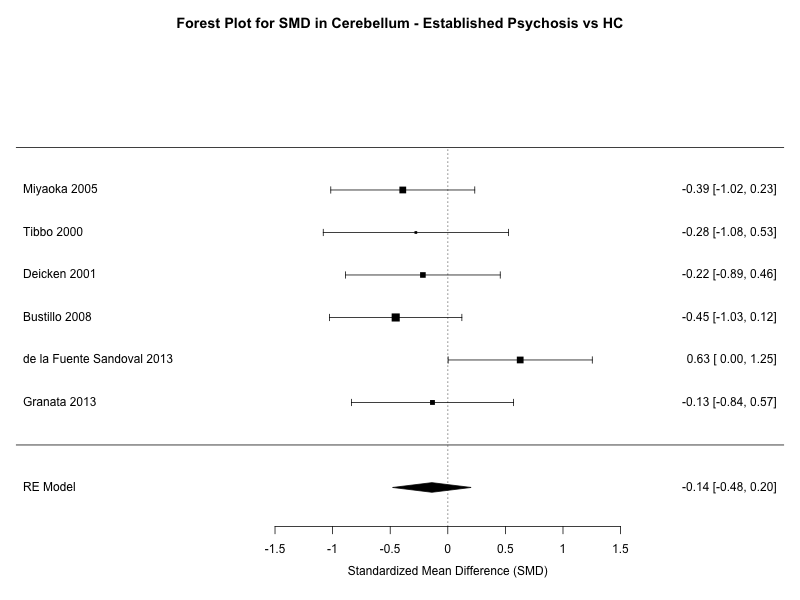


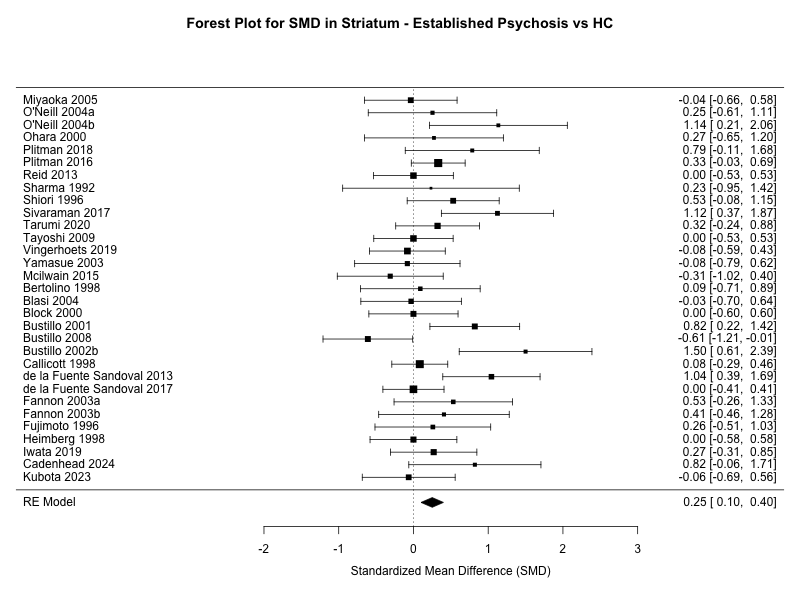


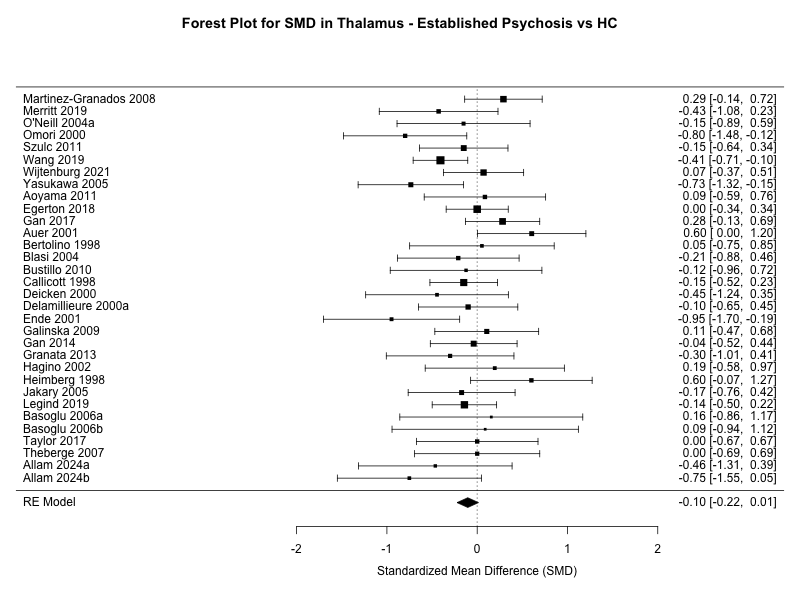


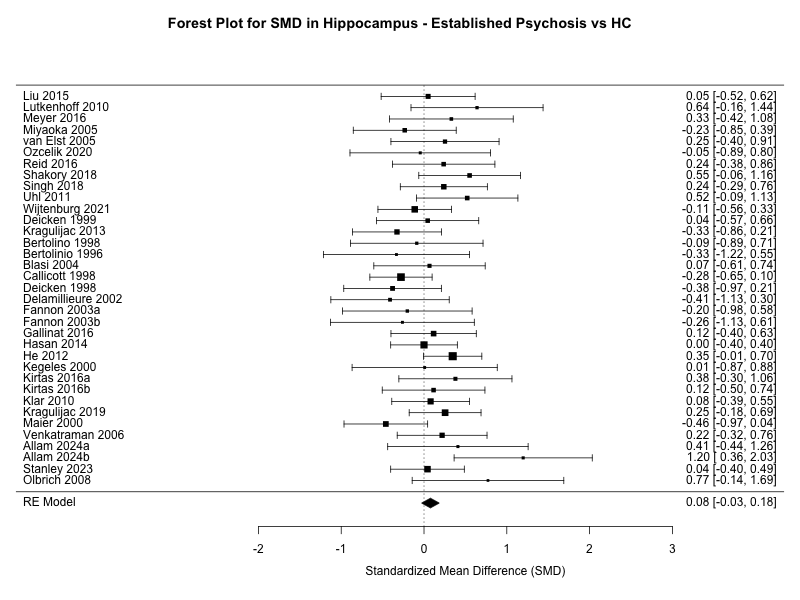


**Supplementary Figures 4A-P: Forest plots of standardised mean differences (SMD) between choline levels in established psychosis and healthy controls.** (A) dlPFC (B) mPFC (C) Other frontal lobe (D) ACC (E) Temporal Lobe (F) Parietal lobe (G) Occipital lobe (H) Global grey matter (I) Global white matter (J) Corpus Callosum (L) Centrum semiovale (M) Cerebellum (N) Striatum (O) Thalamus (P) Hippocampus. Negative SMD indicates lower choline levels in cases, positive SMD indicates higher choline levels in cases. dlPFC indicates dorsolateral prefrontal cortex; mPFC, medial prefrontal cortex; ACC, anterior cingulate cortex; PCC, posterior cingulate cortex; HC, healthy controls.


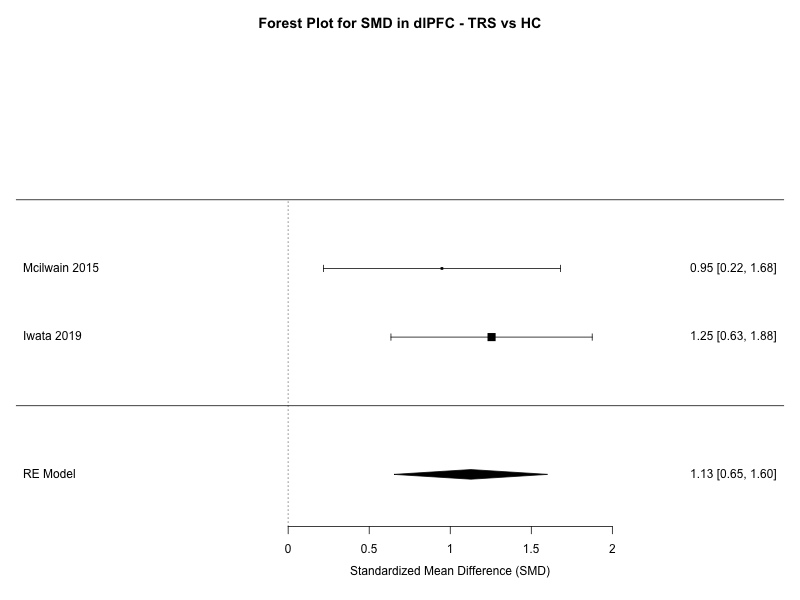


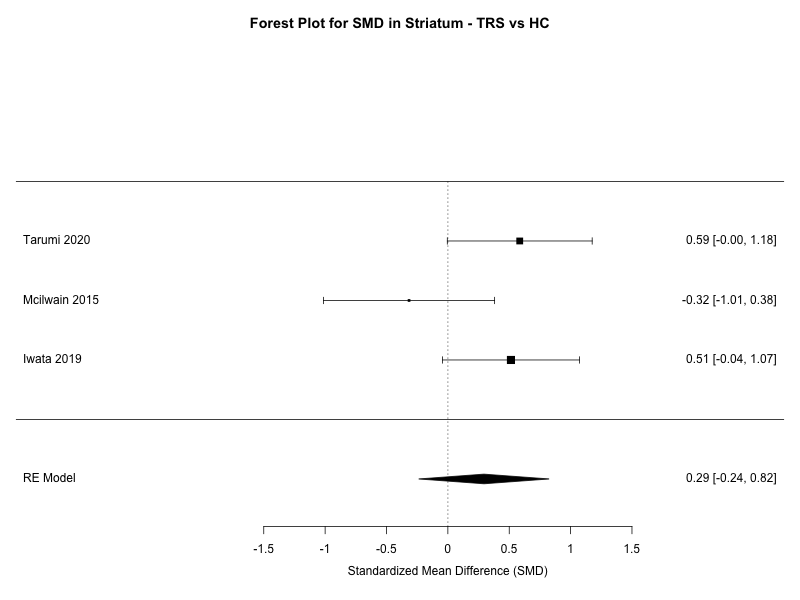


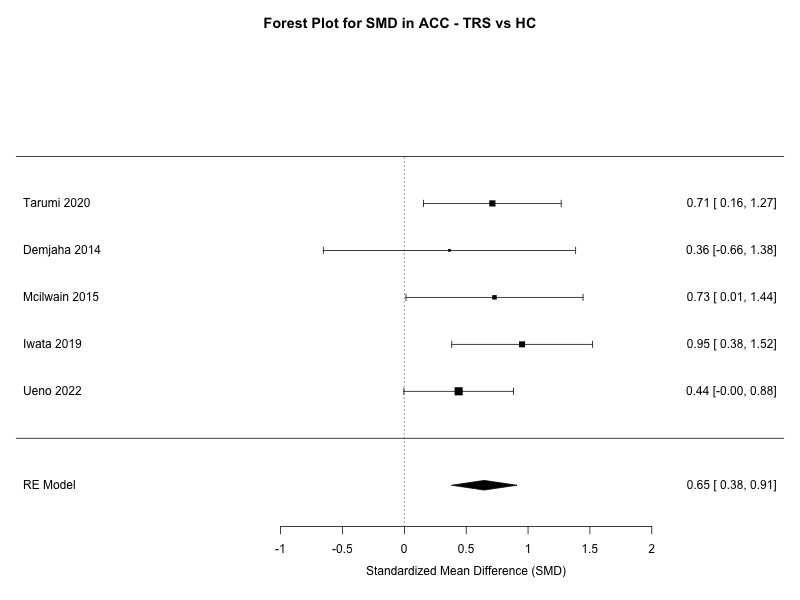


**Supplementary Figures 5A-C: Forest plots of standardised mean differences (SMD) between choline levels in treatment resistant schizophrenia and healthy controls.** (A) dlPFC (B) striatum (C) ACC. Negative SMD indicates lower choline levels in cases, positive SMD indicates higher choline levels in cases. dlPFC indicates dorsolateral prefrontal cortex; ACC, anterior cingulate cortex; TRS, treatment resistant schizophrenia; HC, healthy controls.

**
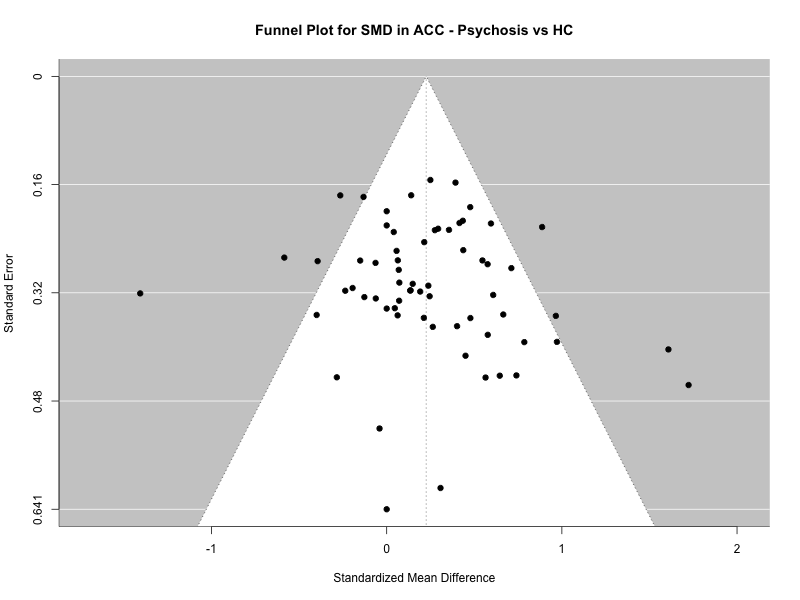
**

**Supplementary Figure 6: Funnel plot for publication bias in SMD meta-analysis for psychosis spectrum disorders in the ACC**

**
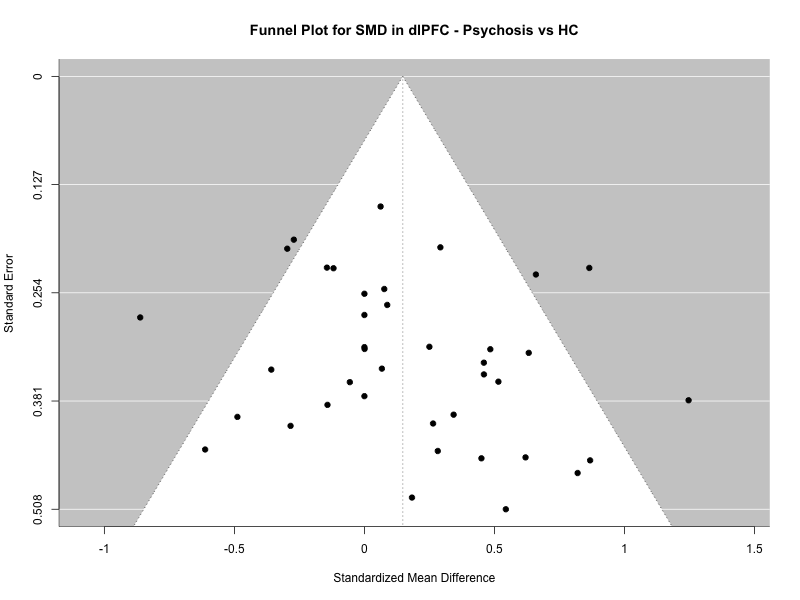
**

**Supplementary Figure 7: Funnel plot for publication bias in SMD meta-analysis for psychosis spectrum disorders in the dlPFC**

**
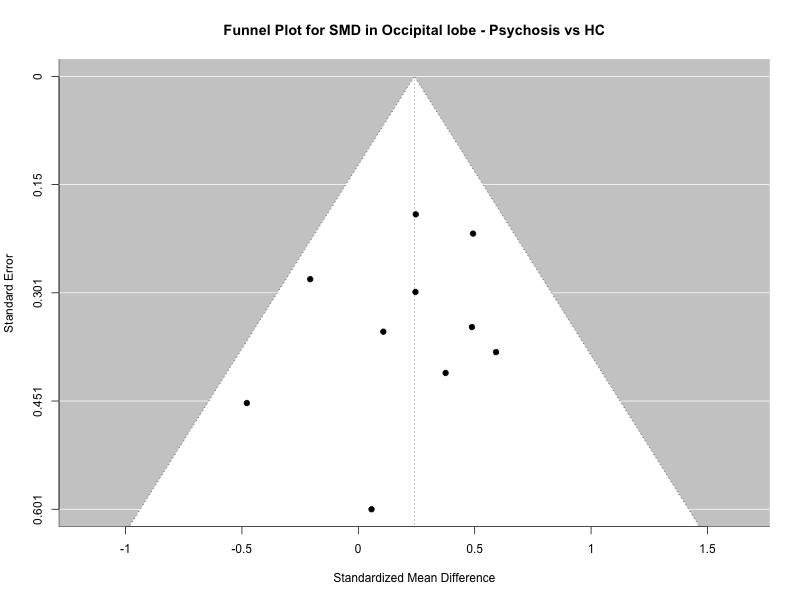
**

**Supplementary Figure 8: Funnel plot for publication bias in SMD meta-analysis for psychosis spectrum disorders in the Occipital lobe**


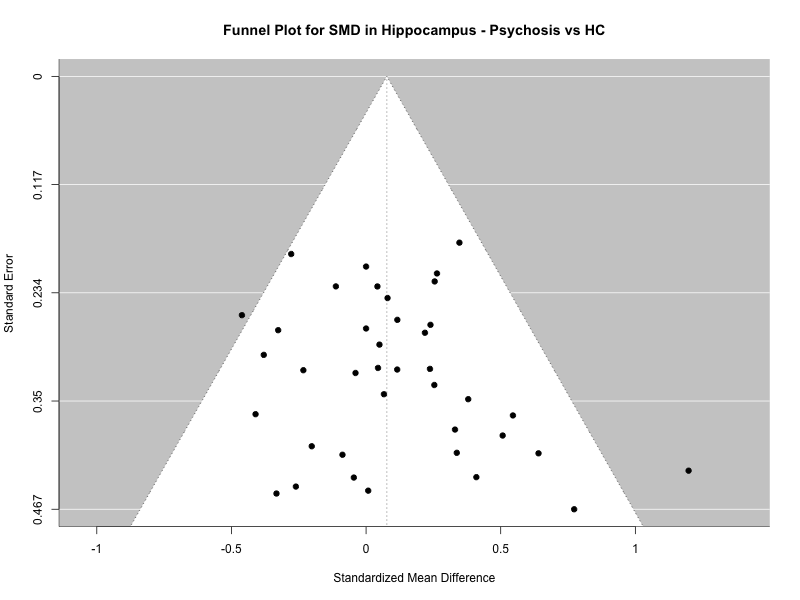


**Supplementary Figure 9: Funnel plot for publication bias in SMD meta-analysis for psychosis spectrum disorders in the hippocampus**

**
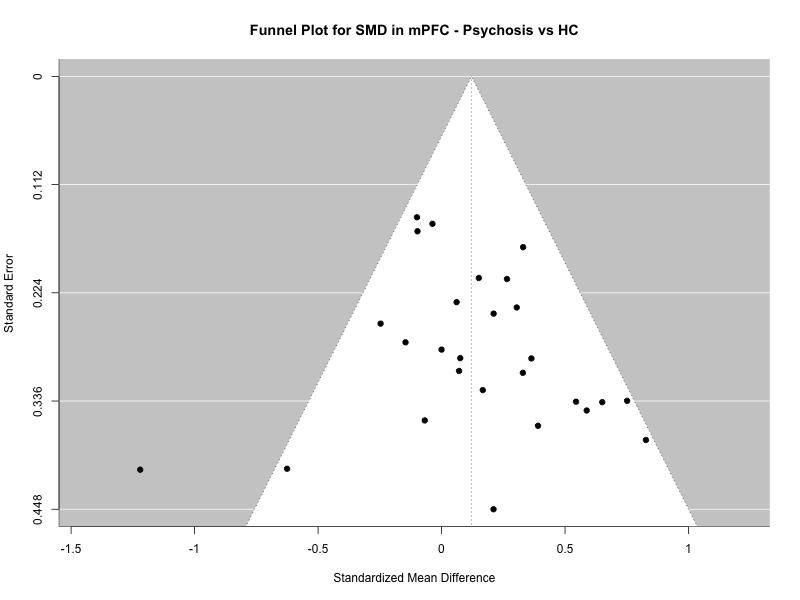
**

**Supplementary Figure 10: Funnel plot for publication bias in SMD meta-analysis for psychosis spectrum disorders in the mPFC**


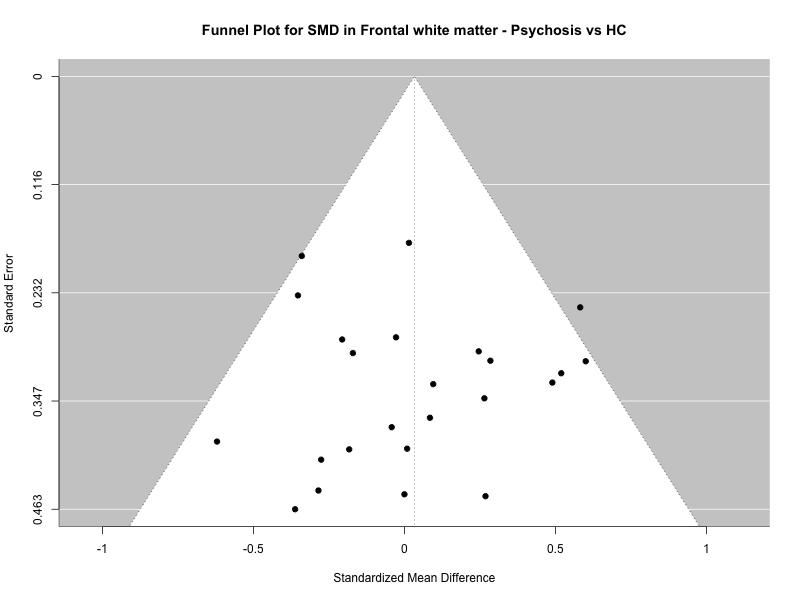


**Supplementary Figure 11: Funnel plot for publication bias in SMD meta-analysis for psychosis spectrum disorders in the frontal white matter**

**
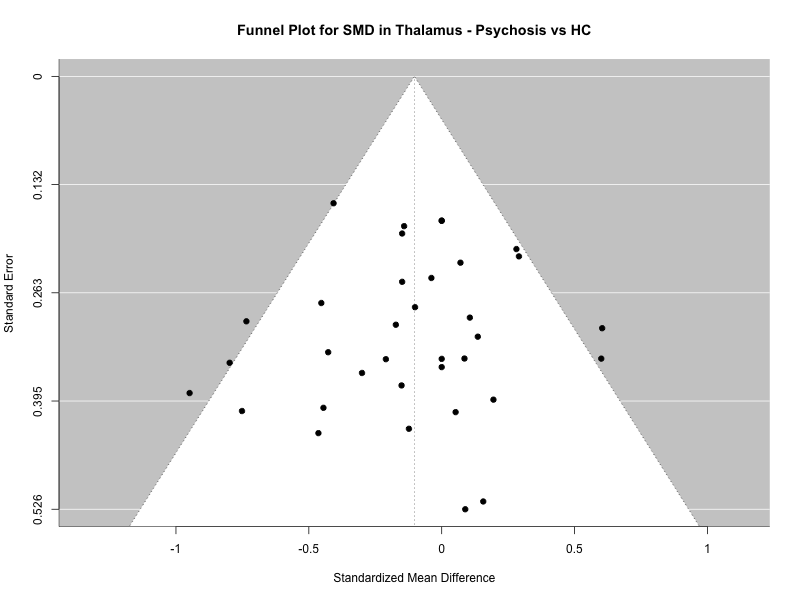
**

**Supplementary Figure 12: Funnel plot for publication bias in SMD meta-analysis for psychosis spectrum disorders in the thalamus**

**
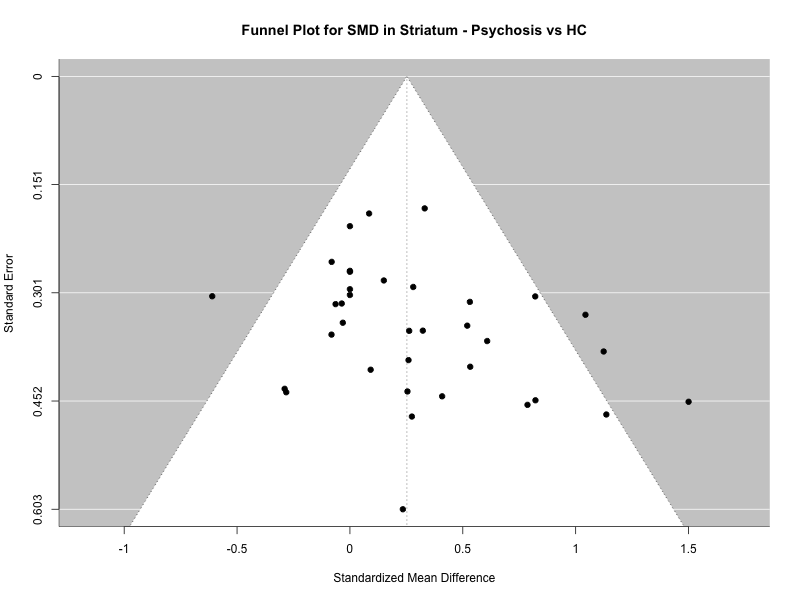
**

**Supplementary Figure 13: Funnel plot for publication bias in SMD meta-analysis for psychosis spectrum disorders in the striatum**


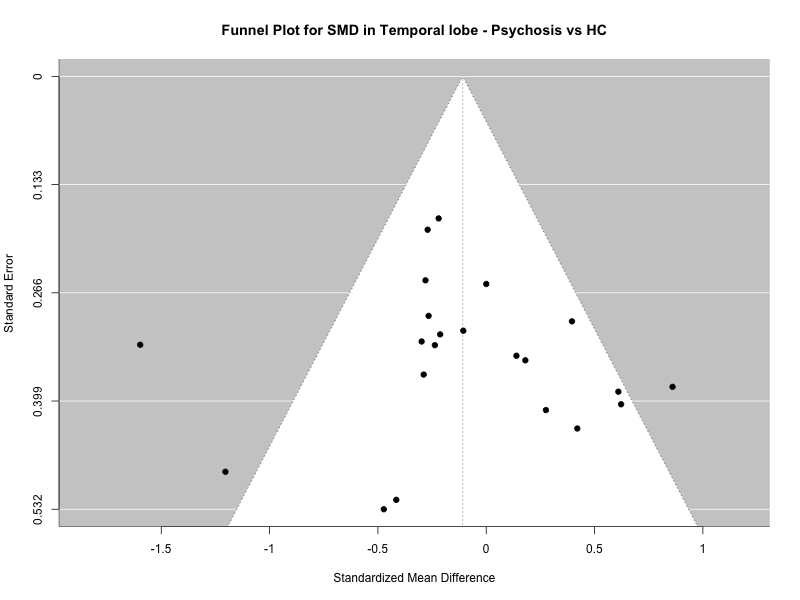


**Supplementary Figure 14: Funnel plot for publication bias in SMD meta-analysis for psychosis spectrum disorders in the temporal lobe**


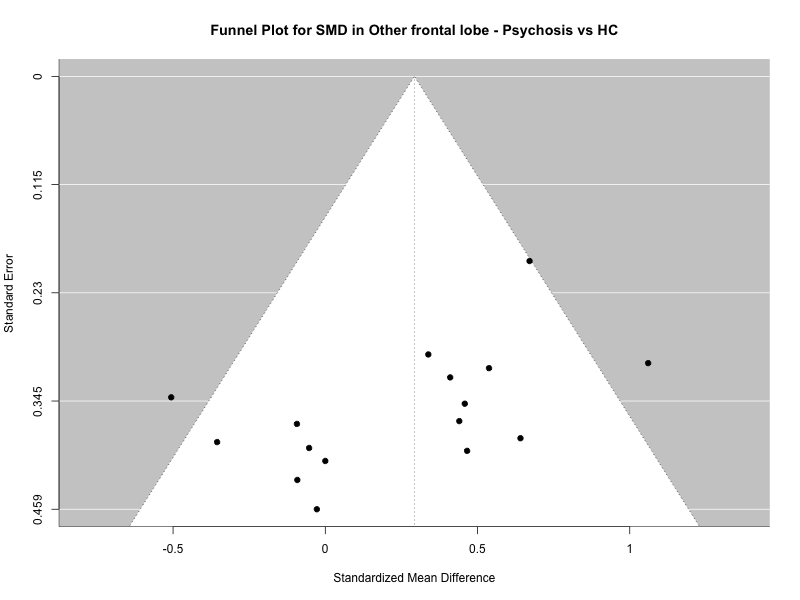


**Supplementary Figure 15: Funnel plot for publication bias in SMD meta-analysis for psychosis spectrum disorders in other frontal lobe regions**

**
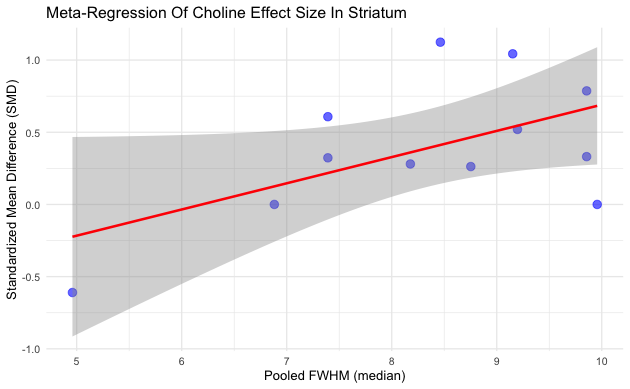
**

**Supplementary Figure 16: Scatter plot of meta-regression of SMD versus FWHM for psychosis spectrum disorders in the striatum**

**
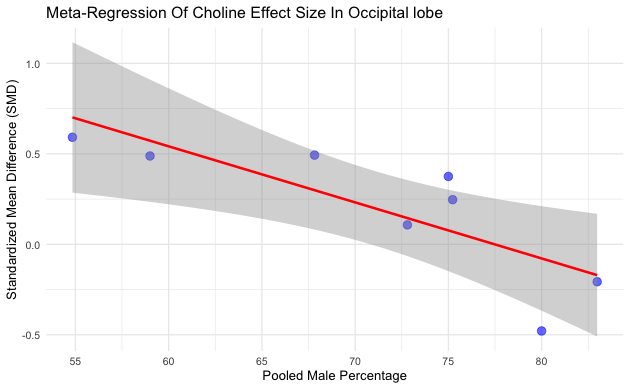
**

**Supplementary Figure 17: Scatter plot of meta-regression of SMD versus male percentage for psychosis spectrum disorders in the occipital lobe**

**
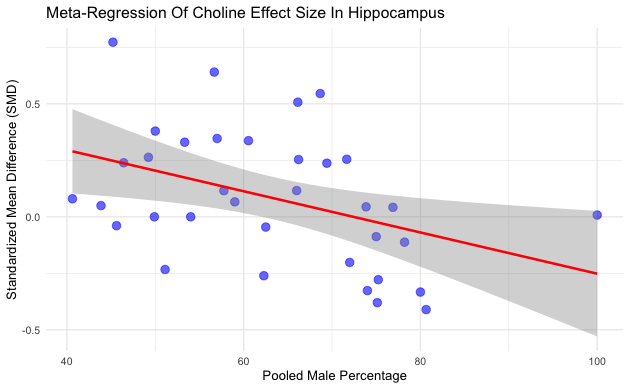
**

**Supplementary Figure 18: Scatter plot of meta-regression of SMD versus male percentage for psychosis spectrum disorders in the hippocampus**

**
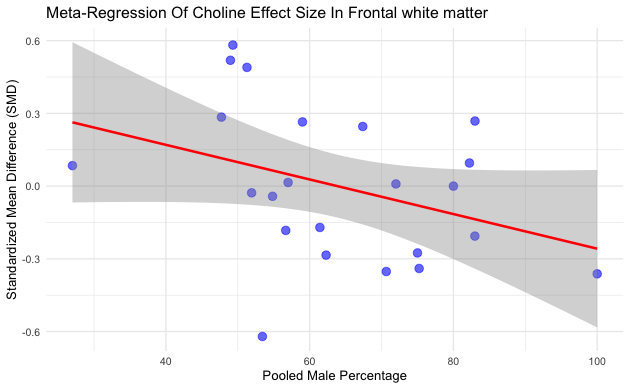
**

**Supplementary Figure 19: Scatter plot of meta-regression of SMD versus male percentage for psychosis spectrum disorders in the frontal white matter**

**
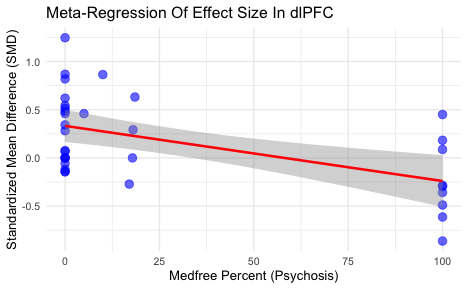
**

**Supplementary Figure 20: Scatter plot of meta-regression of SMD versus medication free percentage for psychosis spectrum disorders in the dlPFC**

**
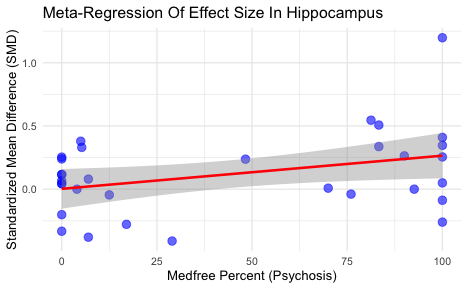
**

**Supplementary Figure 21: Scatter plot of meta-regression of SMD versus medication free percentage for psychosis spectrum disorders in the hippocampus**

**
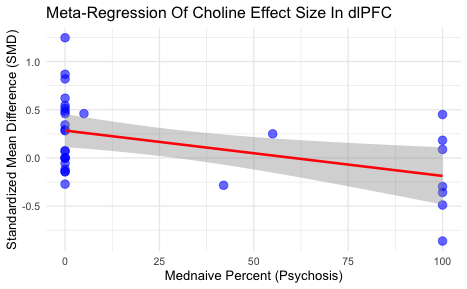
**

**Supplementary Figure 22: Scatter plot of meta-regression of SMD versus medication naive percentage for psychosis spectrum disorders in the dlPFC**

**
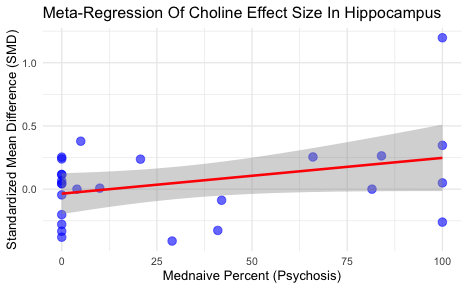
**

**Supplementary Figure 23: Scatter plot of meta-regression of SMD versus medication naive percentage for psychosis spectrum disorders in the hippocampus**

**
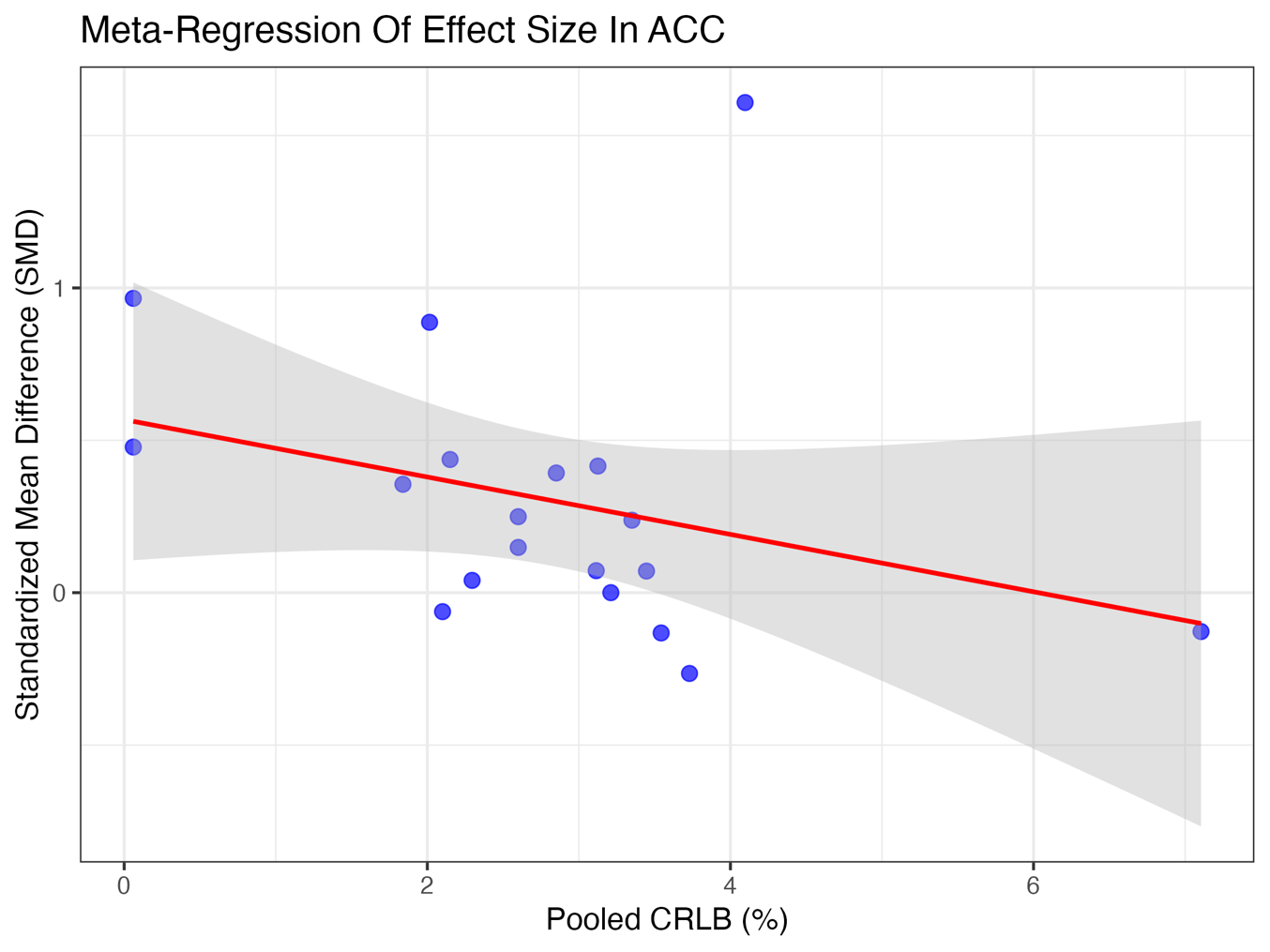
**

**Supplementary Figure 24: Scatter plot of meta-regression of SMD versus CRLB percentage for psychosis spectrum disorders in the ACC**

**
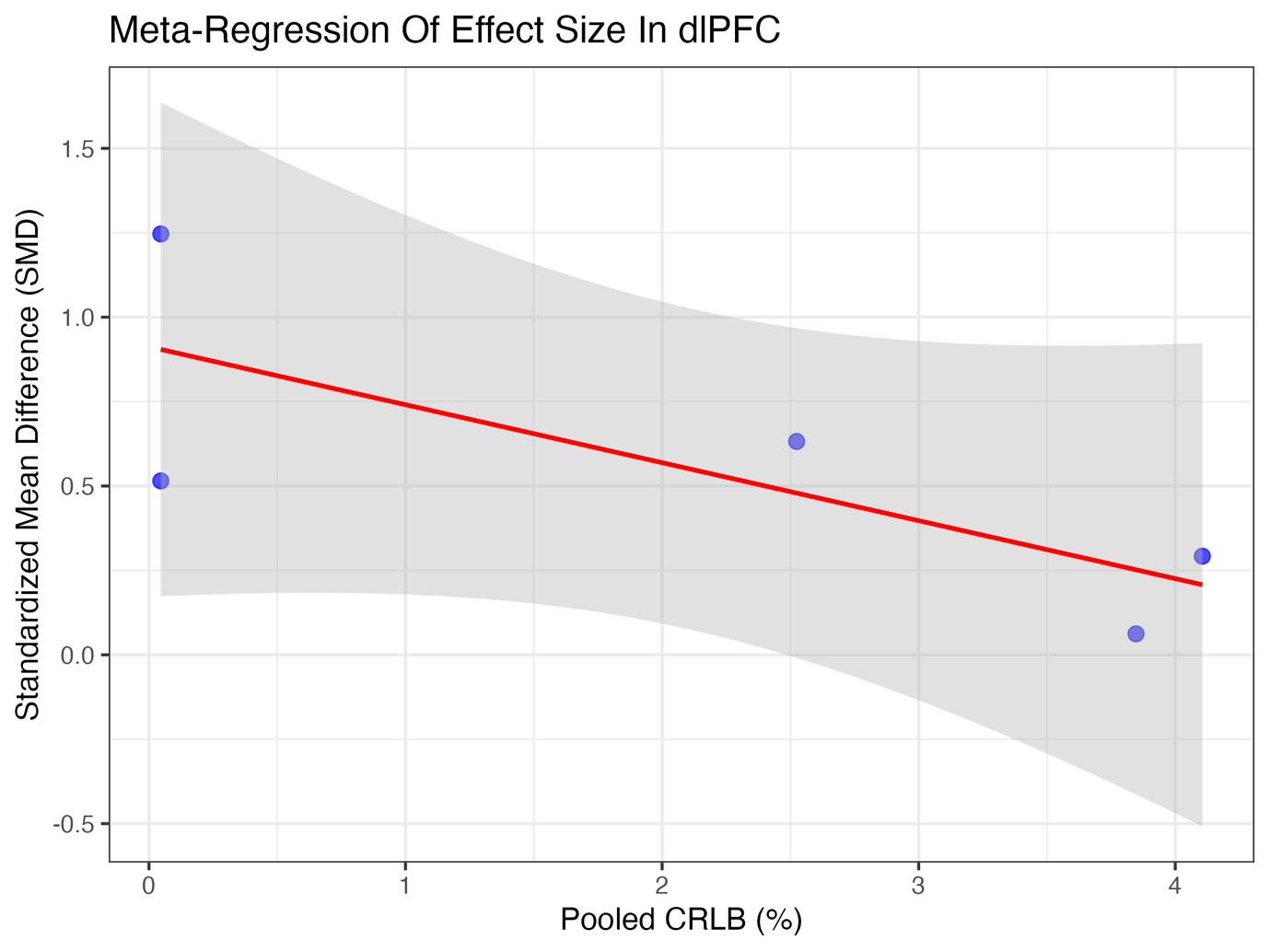
**

**Supplementary Figure 25: Scatter plot of meta-regression of SMD versus CRLB percentage for psychosis spectrum disorders in the dlPFC**

**
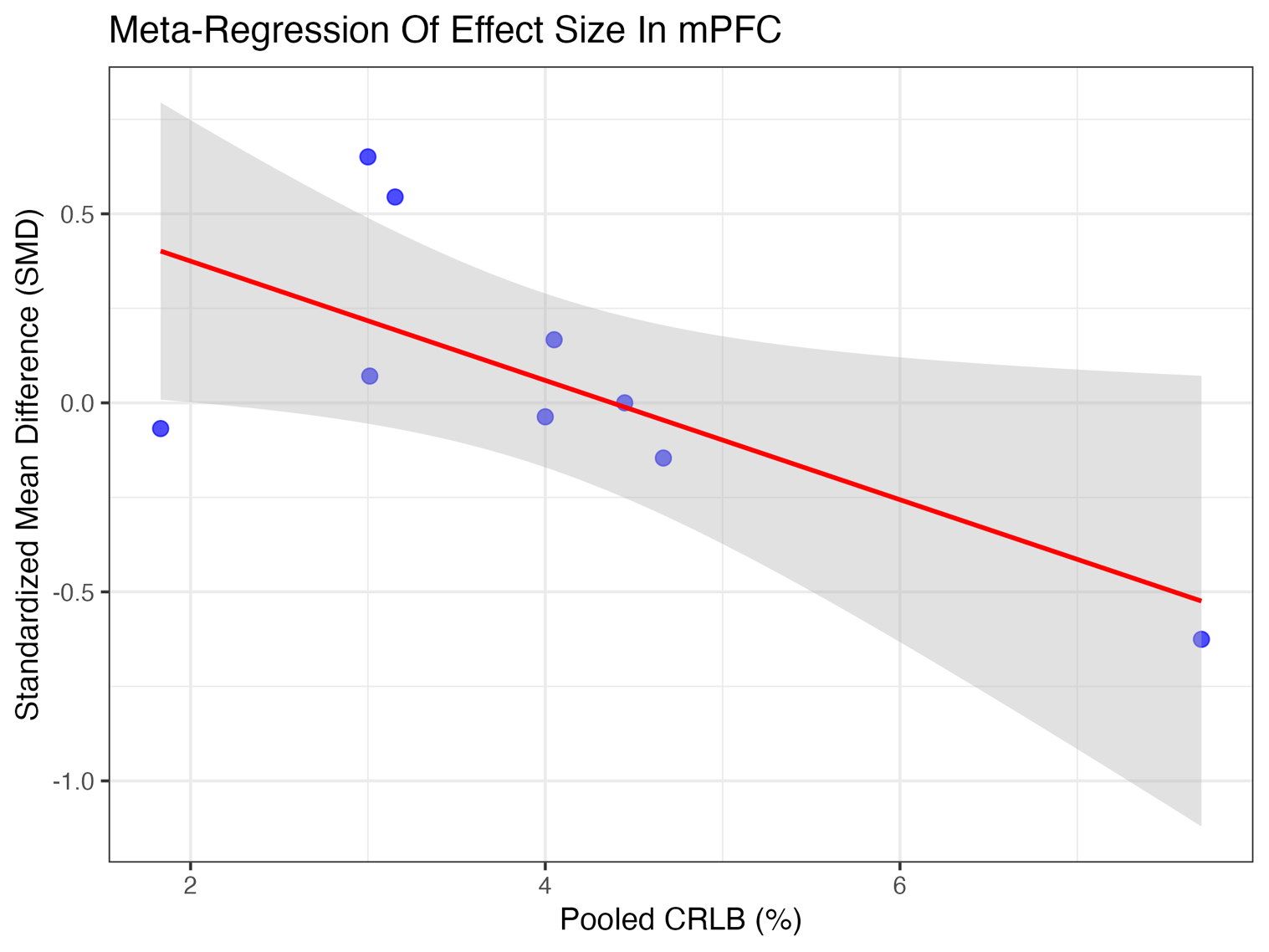
**

**Supplementary Figure 26: Scatter plot of meta-regression of SMD versus CRLB percentage for psychosis spectrum disorders in the mPFC**

**
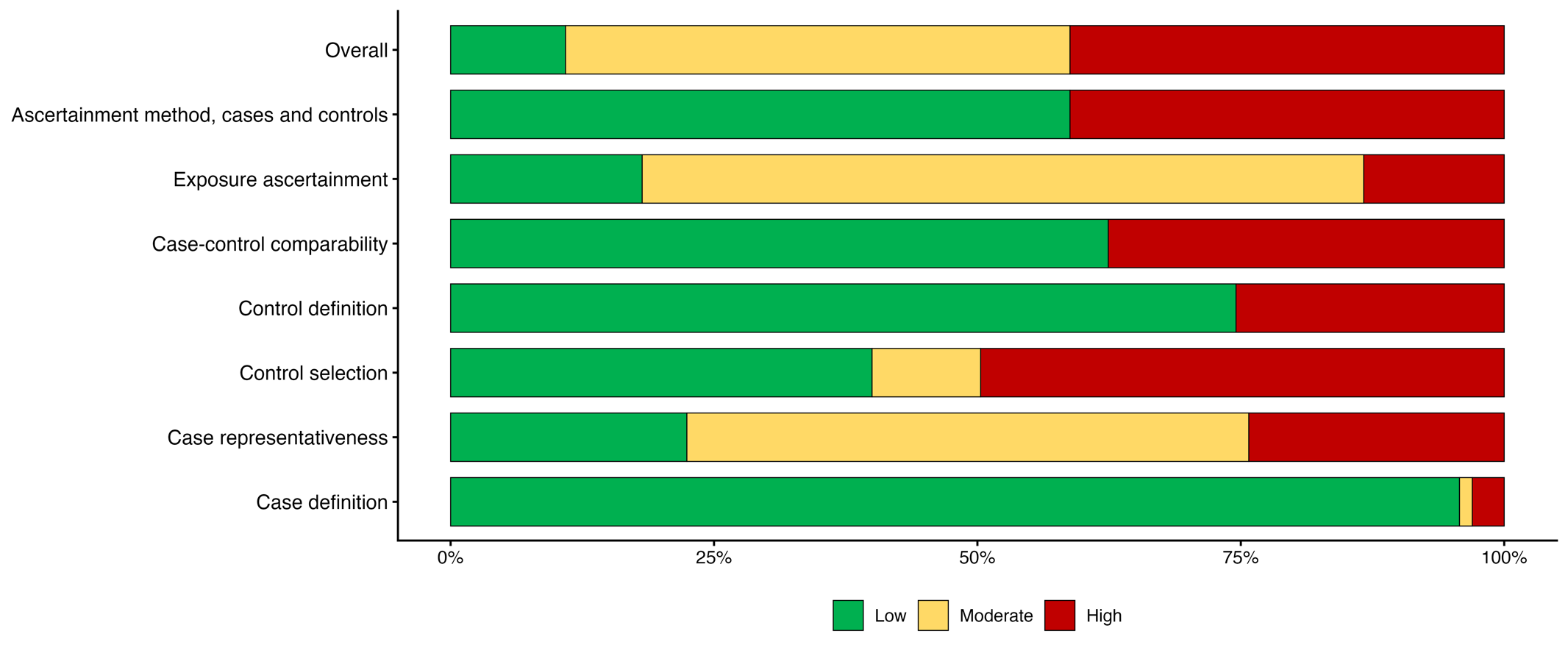
**

**Supplementary Figure 27: Risk of bias findings**

**Supplementary bibliography**

1. Hardy CJ, Tal A, Babb JS, et al: Multivoxel proton MR spectroscopy used to distinguish anterior cingulate metabolic abnormalities in patients with schizophrenia. Radiology 2011; **261**: 542–550.

2. Modinos G, McLaughlin A, Egerton A, et al: Corticolimbic hyper-response to emotion and glutamatergic function in people with high schizotypy: a multimodal fMRI-MRS study. Transl. Psychiatry 2017; **7**: e1083.

3. Reid MA, Salibi N, White DM, et al: 7T Proton Magnetic Resonance Spectroscopy of the Anterior Cingulate Cortex in First-Episode Schizophrenia. Schizophr. Bull. 2019; **45**: 180–189.

4. Rowland LM, Pradhan S, Korenic S, et al: Elevated brain lactate in schizophrenia: a 7 T magnetic resonance spectroscopy study. Transl. Psychiatry 2016; **6**: e967.

5. Kübler U: Structured Clinical Interview for DSM-IV (SCID). In: Encyclopedia of Behavioral Medicine. Edited by MD Gellman and JR Turner. New York, NY: Springer 2013; pp 1919–1920. Available at: https://doi.org/10.1007/978-1-4419-1005-9_66, accessed May 25, 2025.

6. Smucny J, Carter CS and Maddock RJ: Magnetic resonance spectroscopic evidence of increased choline in the dorsolateral prefrontal and visual cortices in recent onset schizophrenia. Neurosci. Lett. 2022; **770**: 136410.

7. Liu W, Yu H, Jiang B, et al: The predictive value of baseline NAA/Cr for treatment response of first-episode schizophrenia: A ^1^H MRS study. Neurosci. Lett. 2015; **600**: 199–205.

8. Lutkenhoff ES, van Erp TG, Thomas MA, et al: Proton MRS in twin pairs discordant for schizophrenia. Mol. Psychiatry 2010; **15**: 308–318.

9. MacKinley M, Ford SD, Jeon P, et al: Central Oxidative Stress and Early Vocational Outcomes in First Episode Psychosis: A 7-Tesla Magnetic Resonance Spectroscopy Study of Glutathione. Schizophr. Bull. 2022; **48**: 921–930.

10. Marsman A, Mandl RCW, Klomp DWJ, et al: GABA and glutamate in schizophrenia: A 7 T 1H-MRS study. NeuroImage Clin. 2014; **6**: 398–407.

11. Martínez-Granados B, Brotons O, Martínez-Bisbal MC, et al: Spectroscopic metabolomic abnormalities in the thalamus related to auditory hallucinations in patients with schizophrenia. Schizophr. Res. 2008; **104**: 13–22.

12. Merritt K, Perez-Iglesias R, Sendt K-V, et al: Remission from antipsychotic treatment in first episode psychosis related to longitudinal changes in brain glutamate. NPJ Schizophr. 2019; **5**: 12.

13. Meyer EJ, Kirov II, Tal A, et al: Metabolic Abnormalities in the Hippocampus of Patients with Schizophrenia: A 3D Multivoxel MR Spectroscopic Imaging Study at 3T. AJNR Am. J. Neuroradiol. 2016; **37**: 2273–2279.

14. Nurnberger JI, Blehar MC, Kaufmann CA, et al: Diagnostic interview for genetic studies. Rationale, unique features, and training. NIMH Genetics Initiative. Arch. Gen. Psychiatry 1994; **51**: 849–859; discussion 863-864.

15. Oishi K: MRI Atlas of Human White Matter. London: Academic Press; 2011.

16. Miyaoka T, Yasukawa R, Mizuno S, et al: Proton magnetic resonance spectroscopy (1H-MRS) of hippocampus, basal ganglia, and vermis of cerebellum in schizophrenia associated with idiopathic unconjugated hyperbilirubinemia (Gilbert’s syndrome). J. Psychiatr. Res. 2005; **39**: 29–34.

17. Molina V, Sánchez J, Reig S, et al: N-acetyl-aspartate levels in the dorsolateral prefrontal cortex in the early years of schizophrenia are inversely related to disease duration. Schizophr. Res. 2005; **73**: 209–219.

18. Molina V, Sánchez J, Sanz J, et al: Dorsolateral prefrontal N-acetyl-aspartate concentration in male patients with chronic schizophrenia and with chronic bipolar disorder. Eur. Psychiatry J. Assoc. Eur. Psychiatr. 2007; **22**: 505–512.

19. Moore CM, Bonello CM, Sherwood AR, et al: Mesial temporal lobe Cho to Cr(PCr) ratio asymmetry in chronic schizophrenics. Schizophr. Res. 2002; **57**: 35–42.

20. Natsubori T, Inoue H, Abe O, et al: Reduced frontal glutamate + glutamine and N-acetylaspartate levels in patients with chronic schizophrenia but not in those at clinical high risk for psychosis or with first-episode schizophrenia. Schizophr. Bull. 2014; **40**: 1128–1139.

21. Woods SW, Walsh BC, Addington J, et al: Current Status Specifiers for Patients at Clinical High Risk for Psychosis. Schizophr. Res. 2014; **158**: 69–75.

22. O’Neill J, Levitt J, Caplan R, et al: 1H MRSI evidence of metabolic abnormalities in childhood-onset schizophrenia. NeuroImage 2004; **21**: 1781–1789.

23. Ohara K, Isoda H, Suzuki Y, et al: Proton magnetic resonance spectroscopy of lenticular nuclei in simple schizophrenia. Prog. Neuropsychopharmacol. Biol. Psychiatry 2000; **24**: 507–519.

24. Ohrmann P, Kugel H, Bauer J, et al: Learning potential on the WCST in schizophrenia is related to the neuronal integrity of the anterior cingulate cortex as measured by proton magnetic resonance spectroscopy. Schizophr. Res. 2008; **106**: 156–163.

25. Rajkowska G and Goldman-Rakic PS: Cytoarchitectonic definition of prefrontal areas in the normal human cortex: II. Variability in locations of areas 9 and 46 and relationship to the Talairach Coordinate System. Cereb. Cortex N. Y. N 1991 1995; **5**: 323–337.

26. Ohrmann P, Siegmund A, Suslow T, et al: Cognitive impairment and in vivo metabolites in first-episode neuroleptic-naive and chronic medicated schizophrenic patients: a proton magnetic resonance spectroscopy study. J. Psychiatr. Res. 2007; **41**: 625–634.

27. van Elst LT, Valerius G, Büchert M, et al: Increased prefrontal and hippocampal glutamate concentration in schizophrenia: evidence from a magnetic resonance spectroscopy study. Biol. Psychiatry 2005; **58**: 724–730.

28. Omori M, Murata T, Kimura H, et al: Thalamic abnormalities in patients with schizophrenia revealed by proton magnetic resonance spectroscopy. Psychiatry Res. 2000; **98**: 155–162.

29. Ongür D, Prescot AP, McCarthy J, et al: Elevated gamma-aminobutyric acid levels in chronic schizophrenia. Biol. Psychiatry 2010; **68**: 667–670.

30. Ota M, Ishikawa M, Sato N, et al: Glutamatergic changes in the cerebral white matter associated with schizophrenic exacerbation. Acta Psychiatr. Scand. 2012; **126**: 72–78.

31. Ota M, Wakabayashi C, Sato N, et al: Effect of L-theanine on glutamatergic function in patients with schizophrenia. Acta Neuropsychiatr. 2015; **27**: 291–296.

32. Özçelik Eroğlu E, Ertuğrul A, Oğuz KK, et al: Effect of Clozapine on Proton Magnetic Resonance Spectroscopy Findings in Hippocampus. Turk Psikiyatri Derg. Turk. J. Psychiatry 2020; **31**: 159–167.

33. Pae C-U, Choe B-Y, Joo R-H, et al: Neuronal dysfunction of the frontal lobe in schizophrenia. Neuropsychobiology 2004; **50**: 211–215.

34. Plitman E, Chavez S, Nakajima S, et al: Striatal neurometabolite levels in patients with schizophrenia undergoing long-term antipsychotic treatment: A proton magnetic resonance spectroscopy and reliability study. Psychiatry Res. Neuroimaging 2018; **273**: 16–24.

35. Sheehan DV, Lecrubier Y, Sheehan KH, et al: The Mini-International Neuropsychiatric Interview (M.I.N.I.): the development and validation of a structured diagnostic psychiatric interview for DSM-IV and ICD-10. J. Clin. Psychiatry 1998; **59 Suppl 20**: 22-33;quiz 34-57.

36. Plitman E, de la Fuente-Sandoval C, Reyes-Madrigal F, et al: Elevated Myo-Inositol, Choline, and Glutamate Levels in the Associative Striatum of Antipsychotic-Naive Patients With First-Episode Psychosis: A Proton Magnetic Resonance Spectroscopy Study With Implications for Glial Dysfunction. Schizophr. Bull. 2016; **42**: 415–424.

37. Reid MA, Kraguljac NV, Avsar KB, et al: Proton magnetic resonance spectroscopy of the substantia nigra in schizophrenia. Schizophr. Res. 2013; **147**: 348–354.

38. Reid MA, Stoeckel LE, White DM, et al: Assessments of function and biochemistry of the anterior cingulate cortex in schizophrenia. Biol. Psychiatry 2010; **68**: 625–633.

39. Reid MA, White DM, Kraguljac NV, et al: A combined diffusion tensor imaging and magnetic resonance spectroscopy study of patients with schizophrenia. Schizophr. Res. 2016; **170**: 341–350.

40. Rowland LM, Kontson K, West J, et al: In vivo measurements of glutamate, GABA, and NAAG in schizophrenia. Schizophr. Bull. 2013; **39**: 1096–1104.

41. Rowland LM, Spieker EA, Francis A, et al: White Matter Alterations in Deficit Schizophrenia. Neuropsychopharmacol. Off. Publ. Am. Coll. Neuropsychopharmacol. 2009; **34**: 1514–1522.

42. Sarramea Crespo F, Luque R, Prieto D, et al: Biochemical changes in the cingulum in patients with schizophrenia and chronic bipolar disorder. Eur. Arch. Psychiatry Clin. Neurosci. 2008; **258**: 394–401.

43. Seese RR, O’Neill J, Hudkins M, et al: Proton magnetic resonance spectroscopy and thought disorder in childhood schizophrenia. Schizophr. Res. 2011; **133**: 82–90.

44. Kaufman J, Birmaher B, Brent D, et al: Schedule for Affective Disorders and Schizophrenia for School-Age Children-Present and Lifetime Version (K-SADS-PL): initial reliability and validity data. J. Am. Acad. Child Adolesc. Psychiatry 1997; **36**: 980–988.

45. Shakory S, Watts JJ, Hafizi S, et al: Hippocampal glutamate metabolites and glial activation in clinical high risk and first episode psychosis. Neuropsychopharmacol. Off. Publ. Am. Coll. Neuropsychopharmacol. 2018; **43**: 2249–2255.

46. Miller TJ, McGlashan TH, Rosen JL, et al: Prodromal assessment with the structured interview for prodromal syndromes and the scale of prodromal symptoms: predictive validity, interrater reliability, and training to reliability. Schizophr. Bull. 2003; **29**: 703–715.

47. Sharma R, Venkatasubramanian PN, Bárány M, et al: Proton magnetic resonance spectroscopy of the brain in schizophrenic and affective patients. Schizophr. Res. 1992; **8**: 43–49.

48. Shioiri T, Hamakawa H, Kato T, et al: Proton magnetic resonance spectroscopy of the basal ganglia in patients with schizophrenia: a preliminary report. Schizophr. Res. 1996; **22**: 19–26.

49. Shirayama Y, Obata T, Matsuzawa D, et al: Specific metabolites in the medial prefrontal cortex are associated with the neurocognitive deficits in schizophrenia: a preliminary study. NeuroImage 2010; **49**: 2783–2790.

50. Sigmundsson T, Maier M, Toone BK, et al: Frontal lobe N-acetylaspartate correlates with psychopathology in schizophrenia: a proton magnetic resonance spectroscopy study. Schizophr. Res. 2003; **64**: 63–71.

51. Singh S, Khushu S, Kumar P, et al: Evidence for regional hippocampal damage in patients with schizophrenia. Neuroradiology 2018; **60**: 199–205.

52. Sivaraman S, Kraguljac NV, White DM, et al: Neurometabolic abnormalities in the associative striatum in antipsychotic-naïve first episode psychosis patients. Psychiatry Res. Neuroimaging 2018; **281**: 101–106.

53. Smesny S, Gussew A, Schack S, et al: Neurometabolic patterns of an “at risk for mental disorders” syndrome involve abnormalities in the thalamus and anterior midcingulate cortex. Schizophr. Res. 2022; **243**: 285–295.

54. Yung AR, Yuen HP, McGorry PD, et al: Mapping the onset of psychosis: the Comprehensive Assessment of At-Risk Mental States. Aust. N. Z. J. Psychiatry 2005; **39**: 964–971.

55. Stanley JA, Williamson PC, Drost DJ, et al: An in vivo proton magnetic resonance spectroscopy study of schizophrenia patients. Schizophr. Bull. 1996; **22**: 597–609.

56. Stone JM, Day F, Tsagaraki H, et al: Glutamate dysfunction in people with prodromal symptoms of psychosis: relationship to gray matter volume. Biol. Psychiatry 2009; **66**: 533–539.

57. Phillips LJ, Yung AR and McGorry PD: Identification of young people at risk of psychosis: validation of Personal Assessment and Crisis Evaluation Clinic intake criteria. Aust. N. Z. J. Psychiatry 2000; **34 Suppl**: S164-169.

58. Szulc A, Galinska B, Tarasow E, et al: Proton magnetic resonance spectroscopy study of brain metabolite changes after antipsychotic treatment. Pharmacopsychiatry 2011; **44**: 148–157.

59. Tanaka Y, Obata T, Sassa T, et al: Quantitative magnetic resonance spectroscopy of schizophrenia: relationship between decreased N-acetylaspartate and frontal lobe dysfunction. Psychiatry Clin. Neurosci. 2006; **60**: 365–372.

60. Tarumi R, Tsugawa S, Noda Y, et al: Levels of glutamatergic neurometabolites in patients with severe treatment-resistant schizophrenia: a proton magnetic resonance spectroscopy study. Neuropsychopharmacol. Off. Publ. Am. Coll. Neuropsychopharmacol. 2020; **45**: 632–640.

61. Howes OD, McCutcheon R, Agid O, et al: Treatment-Resistant Schizophrenia: Treatment Response and Resistance in Psychosis (TRRIP) Working Group Consensus Guidelines on Diagnosis and Terminology. Am. J. Psychiatry 2017; **174**: 216–229.

62. Tayoshi S, Sumitani S, Taniguchi K, et al: Metabolite changes and gender differences in schizophrenia using 3-Tesla proton magnetic resonance spectroscopy (1H-MRS). Schizophr. Res. 2009; **108**: 69–77.

63. Tibbo P, Hanstock C, Asghar S, et al: Proton magnetic resonance spectroscopy (1H-MRS) of the cerebellum in men with schizophrenia. J. Psychiatry Neurosci. 2000; **25**: 509–512.

64. Tibbo PG, Bernier D, Hanstock CC, et al: 3-T proton magnetic spectroscopy in unmedicated first episode psychosis: a focus on creatine. Magn. Reson. Med. 2013; **69**: 613–620.

65. Uhl I, Mavrogiorgou P, Norra C, et al: 1 H-MR spectroscopy in ultra-high risk and first episode stages of schizophrenia. J. Psychiatr. Res. 2011; **45**: 1135–1139.

66. Vingerhoets C, Bakker G, Schrantee A, et al: Influence of muscarinic M1 receptor antagonism on brain choline levels and functional connectivity in medication-free subjects with psychosis: A placebo controlled, cross-over study. Psychiatry Res. Neuroimaging 2019; **290**: 5–13.

67. Andreasen NC, Flaum M and Arndt S: The Comprehensive Assessment of Symptoms and History (CASH). An instrument for assessing diagnosis and psychopathology. Arch. Gen. Psychiatry 1992; **49**: 615–623.

68. Wang AM, Pradhan S, Coughlin JM, et al: Assessing Brain Metabolism With 7-T Proton Magnetic Resonance Spectroscopy in Patients With First-Episode Psychosis. JAMA Psychiatry 2019; **76**: 314–323.

69. Wang Q, Ren H, Li C, et al: Metabolite differences in the medial prefrontal cortex in schizophrenia patients with and without persistent auditory verbal hallucinations: a 1H MRS study. Transl. Psychiatry 2022; **12**: 116.

70. Wijtenburg SA, Wang M, Korenic SA, et al: Metabolite Alterations in Adults With Schizophrenia, First Degree Relatives, and Healthy Controls: A Multi-Region 7T MRS Study. Front. Psychiatry 2021; **12**: 656459.

71. Wijtenburg SA, Wright SN, Korenic SA, et al: Altered Glutamate and Regional Cerebral Blood Flow Levels in Schizophrenia: A 1H-MRS and pCASL study. Neuropsychopharmacol. Off. Publ. Am. Coll. Neuropsychopharmacol. 2017; **42**: 562–571.

72. Wood SJ, Berger G, Velakoulis D, et al: Proton magnetic resonance spectroscopy in first episode psychosis and ultra high-risk individuals. Schizophr. Bull. 2003; **29**: 831–843.

73. Yamasue H, Fukui T, Fukuda R, et al: Drug-induced parkinsonism in relation to choline-containing compounds measured by 1H-MR spectroscopy in putamen of chronically medicated patients with schizophrenia. Int. J. Neuropsychopharmacol. 2003; **6**: 353–360.

74. Yamasue H, Fukui T, Fukuda R, et al: 1H-MR spectroscopy and gray matter volume of the anterior cingulate cortex in schizophrenia. Neuroreport 2002; **13**: 2133–2137.

75. Yasukawa R, Miyaoka T, Mizuno S, et al: Proton magnetic resonance spectroscopy of the anterior cingulate gyrus, insular cortex and thalamus in schizophrenia associated with idiopathic unconjugated hyperbilirubinemia (Gilbert’s syndrome). J. Psychiatry Neurosci. 2005; **30**: 416–422.

76. Zabala A, Sánchez-González J, Parellada M, et al: Findings of proton magnetic resonance spectometry in the dorsolateral prefrontal cortex in adolescents with first episodes of psychosis. Psychiatry Res. 2007; **156**: 33–42.

77. Öngür D, Jensen JE, Prescot AP, et al: Abnormal glutamatergic neurotransmission and neuronal-glial interactions in acute mania. Biol. Psychiatry 2008; **64**: 718–726.

78. Aoyama N, Théberge J, Drost DJ, et al: Grey matter and social functioning correlates of glutamatergic metabolite loss in schizophrenia. Br. J. Psychiatry J. Ment. Sci. 2011; **198**: 448–456.

79. Birur B, Kraguljac NV, VerHoef L, et al: Neurometabolic correlates of 6 and 16 weeks of treatment with risperidone in medication-naive first-episode psychosis patients. Transl. Psychiatry 2020; **10**: 15.

80. Blüml S, Tan J, Harris K, et al: Quantitative proton-decoupled 31P MRS of the schizophrenic brain in vivo. J. Comput. Assist. Tomogr. 1999; **23**: 272–275.

81. Bossong MG, Antoniades M, Azis M, et al: Association of Hippocampal Glutamate Levels With Adverse Outcomes in Individuals at Clinical High Risk for Psychosis. JAMA Psychiatry 2019; **76**: 199–207.

82. Yung AR, Phillips LJ, McGorry PD, et al: Prediction of psychosis. A step towards indicated prevention of schizophrenia. Br. J. Psychiatry. Suppl. 1998; **172**: 14–20.

83. Bustillo JR, Chen H, Gasparovic C, et al: Glutamate as a marker of cognitive function in schizophrenia: a proton spectroscopic imaging study at 4 Tesla. Biol. Psychiatry 2011; **69**: 19–27.

84. Bustillo JR, Rowland LM, Mullins P, et al: 1H-MRS at 4 tesla in minimally treated early schizophrenia. Mol. Psychiatry 2010; **15**: 629–636.

85. Da Silva T, Hafizi S, Rusjan PM, et al: GABA levels and TSPO expression in people at clinical high risk for psychosis and healthy volunteers: a PET-MRS study. J. Psychiatry Neurosci. JPN 2019; **44**: 111–119.

86. Deicken RF, Feiwell R, Schuff N, et al: Evidence for altered cerebellar vermis neuronal integrity in schizophrenia. Psychiatry Res. 2001; **107**: 125–134.

87. Deicken RF, Pegues M and Amend D: Reduced hippocampal N-acetylaspartate without volume loss in schizophrenia. Schizophr. Res. 1999; **37**: 217–223.

88. Demjaha A, Egerton A, Murray RM, et al: Antipsychotic treatment resistance in schizophrenia associated with elevated glutamate levels but normal dopamine function. Biol. Psychiatry 2014; **75**: e11-13.

89. Conley RR and Kelly DL: Management of treatment resistance in schizophrenia. Biol. Psychiatry 2001; **50**: 898–911.

90. McGuffin P and Farmer A: Polydiagnostic approaches to measuring and classifying psychopathology. Am. J. Med. Genet. 2001; **105**: 39–41.

91. Egerton A, Broberg BV, Van Haren N, et al: Response to initial antipsychotic treatment in first episode psychosis is related to anterior cingulate glutamate levels: a multicentre 1H-MRS study (OPTiMiSE). Mol. Psychiatry 2018; **23**: 2145–2155.

92. Gan J-L, Duan H-F, Cheng Z-X, et al: Neuroprotective Effect of Modified Electroconvulsive Therapy for Schizophrenia: A Proton Magnetic Resonance Spectroscopy Study. J. Nerv. Ment. Dis. 2017; **205**: 480–486.

93. Goldstein ME, Anderson VM, Pillai A, et al: Glutamatergic neurometabolites in clozapine-responsive and -resistant schizophrenia. Int. J. Neuropsychopharmacol. 2015; **18**: pyu117.

94. Kraguljac NV, White DM, Reid MA, et al: Increased hippocampal glutamate and volumetric deficits in unmedicated patients with schizophrenia. JAMA Psychiatry 2013; **70**: 1294–1302.

95. Larabi DI, Liemburg EJ, Pijnenborg GHM, et al: Association between prefrontal N-acetylaspartate and insight in psychotic disorders. Schizophr. Res. 2017; **179**: 112–118.

96. Lim KO, Adalsteinsson E, Spielman D, et al: Proton magnetic resonance spectroscopic imaging of cortical gray and white matter in schizophrenia. Arch. Gen. Psychiatry 1998; **55**: 346–352.

97. Auer DP, Wilke M, Grabner A, et al: Reduced NAA in the thalamus and altered membrane and glial metabolism in schizophrenic patients detected by 1H-MRS and tissue segmentation. Schizophr. Res. 2001; **52**: 87–99.

98. Aydin K, Ucok A and Guler J: Altered metabolic integrity of corpus callosum among individuals at ultra high risk of schizophrenia and first-episode patients. Biol. Psychiatry 2008; **64**: 750–757.

99. Bartolomeo LA, Wright AM, Ma RE, et al: Relationship of auditory electrophysiological responses to magnetic resonance spectroscopy metabolites in Early Phase Psychosis. Int. J. Psychophysiol. Off. J. Int. Organ. Psychophysiol. 2019; **145**: 15–22.

100. Bertolino A, Kumra S, Callicott JH, et al: Common pattern of cortical pathology in childhood-onset and adult-onset schizophrenia as identified by proton magnetic resonance spectroscopic imaging. Am. J. Psychiatry 1998; **155**: 1376–1383.

101. Bertolino A, Nawroz S, Mattay VS, et al: Regionally specific pattern of neurochemical pathology in schizophrenia as assessed by multislice proton magnetic resonance spectroscopic imaging. Am. J. Psychiatry 1996; **153**: 1554–1563.

102. Blasi G, Bertolino A, Brudaglio F, et al: Hippocampal neurochemical pathology in patients at first episode of affective psychosis: a proton magnetic resonance spectroscopic imaging study. Psychiatry Res. 2004; **131**: 95–105.

103. Block W, Bayer TA, Tepest R, et al: Decreased frontal lobe ratio of N-acetyl aspartate to choline in familial schizophrenia: a proton magnetic resonance spectroscopy study. Neurosci. Lett. 2000; **289**: 147–151.

104. Endicott J and Spitzer RL: A diagnostic interview: the schedule for affective disorders and schizophrenia. Arch. Gen. Psychiatry 1978; **35**: 837–844.

105. Brandt AS, Unschuld PG, Pradhan S, et al: Age-related changes in anterior cingulate cortex glutamate in schizophrenia: A (1)H MRS Study at 7 Tesla. Schizophr. Res. 2016; **172**: 101–105.

106. Brooks WM, Hodde-Vargas J, Vargas LA, et al: Frontal lobe of children with schizophrenia spectrum disorders: a proton magnetic resonance spectroscopic study. Biol. Psychiatry 1998; **43**: 263–269.

107. Bustillo JR, Chen H, Jones T, et al: Increased glutamine in patients undergoing long-term treatment for schizophrenia: a proton magnetic resonance spectroscopy study at 3 T. JAMA Psychiatry 2014; **71**: 265–272.

108. Bustillo JR, Jones T, Chen H, et al: Glutamatergic and Neuronal Dysfunction in Gray and White Matter: A Spectroscopic Imaging Study in a Large Schizophrenia Sample. Schizophr. Bull. 2017; **43**: 611–619.

109. Bustillo JR, Jones T, Qualls C, et al: Proton magnetic resonance spectroscopic imaging of gray and white matter in bipolar-I and schizophrenia. J. Affect. Disord. 2019; **246**: 745–753.

110. Bustillo JR, Lauriello J, Rowland LM, et al: Effects of chronic haloperidol and clozapine treatments on frontal and caudate neurochemistry in schizophrenia. Psychiatry Res. 2001; **107**: 135–149.

111. Bustillo JR, Lauriello J, Rowland LM, et al: Longitudinal follow-up of neurochemical changes during the first year of antipsychotic treatment in schizophrenia patients with minimal previous medication exposure. Schizophr. Res. 2002; **58**: 313–321.

112. Bustillo JR, Rowland LM, Jung R, et al: Proton magnetic resonance spectroscopy during initial treatment with antipsychotic medication in schizophrenia. Neuropsychopharmacol. Off. Publ. Am. Coll. Neuropsychopharmacol. 2008; **33**: 2456–2466.

113. First MB, PhD JBWW, Karg RS, et al: Structured Clinical Interview for DSM-5® Disorders―Clinician Version. Arlington, VA: American Psychiatric Association Publishing; 2016.

114. Bustillo JR, Rowland LM, Lauriello J, et al: High choline concentrations in the caudate nucleus in antipsychotic-naive patients with schizophrenia. Am. J. Psychiatry 2002; **159**: 130–133.

115. Byun MS, Choi J-S, Yoo SY, et al: Depressive Symptoms and Brain Metabolite Alterations in Subjects at Ultra-high Risk for Psychosis: A Preliminary Study. Psychiatry Investig. 2009; **6**: 264–271.

116. Callicott JH, Egan MF, Bertolino A, et al: Hippocampal N-acetyl aspartate in unaffected siblings of patients with schizophrenia: a possible intermediate neurobiological phenotype. Biol. Psychiatry 1998; **44**: 941–950.

117. Cecil KM, Lenkinski RE, Gur RE, et al: Proton magnetic resonance spectroscopy in the frontal and temporal lobes of neuroleptic naive patients with schizophrenia. Neuropsychopharmacol. Off. Publ. Am. Coll. Neuropsychopharmacol. 1999; **20**: 131–140.

118. Chang L, Friedman J, Ernst T, et al: Brain metabolite abnormalities in the white matter of elderly schizophrenic subjects: implication for glial dysfunction. Biol. Psychiatry 2007; **62**: 1396–1404.

119. Chiu PW, Lui SSY, Hung KSY, et al: In vivo gamma-aminobutyric acid and glutamate levels in people with first-episode schizophrenia: A proton magnetic resonance spectroscopy study. Schizophr. Res. 2018; **193**: 295–303.

120. Crocker CE, Bernier DC, Hanstock CC, et al: Prefrontal glutamate levels differentiate early phase schizophrenia and methamphetamine addiction: a (1)H MRS study at 3Tesla. Schizophr. Res. 2014; **157**: 231–237.

121. de la Fuente-Sandoval C, León-Ortiz P, Azcárraga M, et al: Glutamate levels in the associative striatum before and after 4 weeks of antipsychotic treatment in first-episode psychosis: a longitudinal proton magnetic resonance spectroscopy study. JAMA Psychiatry 2013; **70**: 1057–1066.

122. de la Fuente-Sandoval C, León-Ortiz P, Favila R, et al: Higher levels of glutamate in the associative-striatum of subjects with prodromal symptoms of schizophrenia and patients with first-episode psychosis. Neuropsychopharmacol. Off. Publ. Am. Coll. Neuropsychopharmacol. 2011; **36**: 1781–1791.

123. de la Fuente-Sandoval C, Reyes-Madrigal F, Mao X, et al: Prefrontal and Striatal Gamma-Aminobutyric Acid Levels and the Effect of Antipsychotic Treatment in First-Episode Psychosis Patients. Biol. Psychiatry 2018; **83**: 475–483.

124. de la Fuente-Sandoval C, Reyes-Madrigal F, Mao X, et al: Cortico-Striatal GABAergic and Glutamatergic Dysregulations in Subjects at Ultra-High Risk for Psychosis Investigated with Proton Magnetic Resonance Spectroscopy. Int. J. Neuropsychopharmacol. 2015; **19**: pyv105.

125. Deicken RF, Johnson C, Eliaz Y, et al: Reduced concentrations of thalamic N-acetylaspartate in male patients with schizophrenia. Am. J. Psychiatry 2000; **157**: 644–647.

126. Deicken RF, Zhou L, Corwin F, et al: Decreased left frontal lobe N-acetylaspartate in schizophrenia. Am. J. Psychiatry 1997; **154**: 688–690.

127. Deicken RF, Zhou L, Schuff N, et al: Hippocampal neuronal dysfunction in schizophrenia as measured by proton magnetic resonance spectroscopy. Biol. Psychiatry 1998; **43**: 483–488.

128. Deicken RF, Zhou L, Schuff N, et al: Proton magnetic resonance spectroscopy of the anterior cingulate region in schizophrenia. Schizophr. Res. 1997; **27**: 65–71.

129. Delamillieure P, Constans J, Fernandez J, et al: Proton magnetic resonance spectroscopy (1H-MRS) of the thalamus in schizophrenia. Eur. Psychiatry J. Assoc. Eur. Psychiatr. 2000; **15**: 489–491.

130. Delamillieure P, Constans J-M, Fernandez J, et al: Proton magnetic resonance spectroscopy (1H MRS) in schizophrenia: investigation of the right and left hippocampus, thalamus, and prefrontal cortex. Schizophr. Bull. 2002; **28**: 329–339.

131. Delamillieure P, Fernandez J, Constans JM, et al: Proton magnetic resonance spectroscopy of the medial prefrontal cortex in patients with deficit schizophrenia: preliminary report. Am. J. Psychiatry 2000; **157**: 641–643.

132. Ende G, Braus DF, Walter S, et al: Lower concentration of thalamic n-acetylaspartate in patients with schizophrenia: a replication study. Am. J. Psychiatry 2001; **158**: 1314–1316.

133. Fannon D, Simmons A, Tennakoon L, et al: Selective deficit of hippocampal N-acetylaspartate in antipsychotic-naive patients with schizophrenia. Biol. Psychiatry 2003; **54**: 587–598.

134. Fujimoto T, Nakano T, Takano T, et al: Proton magnetic resonance spectroscopy of basal ganglia in chronic schizophrenia. Biol. Psychiatry 1996; **40**: 14–18.

135. Fukuzako H: Heritability heightens brain metabolite differences in schizophrenia. J. Neuropsychiatry Clin. Neurosci. 2000; **12**: 95–97.

136. Fukuzako H, Takeuchi K, Hokazono Y, et al: Proton magnetic resonance spectroscopy of the left medial temporal and frontal lobes in chronic schizophrenia: preliminary report. Psychiatry Res. 1995; **61**: 193–200.

137. Galińska B, Szulc A, Tarasów E, et al: Duration of untreated psychosis and proton magnetic resonance spectroscopy (1H-MRS) findings in first-episode schizophrenia. Med. Sci. Monit. Int. Med. J. Exp. Clin. Res. 2009; **15**: CR82-88.

138. World Health Organization: International statistical classification of diseases and related health problems (10th Revision). 10th ed.; 2016.

139. Galińska-Skok B, Małus A, Konarzewska B, et al: Choline Compounds of the Frontal Lobe and Temporal Glutamatergic System in Bipolar and Schizophrenia Proton Magnetic Resonance Spectroscopy Study. Dis. Markers 2018; **2018**: 3654894.

140. Gallinat J, McMahon K, Kühn S, et al: Cross-sectional Study of Glutamate in the Anterior Cingulate and Hippocampus in Schizophrenia. Schizophr. Bull. 2016; **42**: 425–433.

141. Gan J-L, Cheng Z-X, Duan H-F, et al: Atypical antipsychotic drug treatment for 6 months restores N-acetylaspartate in left prefrontal cortex and left thalamus of first-episode patients with early onset schizophrenia: A magnetic resonance spectroscopy study. Psychiatry Res. 2014; **223**: 23–27.

142. Granata F, Pandolfo G, Vinci S, et al: Proton magnetic resonance spectroscopy (H-MRS) in chronic schizophrenia. A single-voxel study in three regions involved in a pathogenetic theory. Neuroradiol. J. 2013; **26**: 277–283.

143. Hagino H, Suzuki M, Mori K, et al: Proton magnetic resonance spectroscopy of the inferior frontal gyrus and thalamus and its relationship to verbal learning task performance in patients with schizophrenia: a preliminary report. Psychiatry Clin. Neurosci. 2002; **56**: 499–507.

144. Hasan A, Wobrock T, Falkai P, et al: Hippocampal integrity and neurocognition in first-episode schizophrenia: a multidimensional study. World J. Biol. Psychiatry Off. J. World Fed. Soc. Biol. Psychiatry 2014; **15**: 188–199.

145. He Y, Kosciolek T, Tang J, et al: Gut microbiome and magnetic resonance spectroscopy study of subjects at ultra-high risk for psychosis may support the membrane hypothesis. Eur. Psychiatry J. Assoc. Eur. Psychiatr. 2018; **53**: 37–45.

146. He Z-L, Deng W, Li M-L, et al: Detection of metabolites in the white matter of frontal lobes and hippocampus with proton in first-episode treatment-naïve schizophrenia patients. Early Interv. Psychiatry 2012; **6**: 166–175.

147. Heimberg C, Komoroski RA, Lawson WB, et al: Regional proton magnetic resonance spectroscopy in schizophrenia and exploration of drug effect. Psychiatry Res. 1998; **83**: 105–115.

148. Huang M, Guo W, Lu S, et al: The relationship between the alterations in metabolite levels in the dorsolateral prefrontal cortex and clinical symptoms of patients with first-episode schizophrenia: a one year follow-up study. Oncotarget 2019; **10**: 606–615.

149. Huang M-L, Khoh T-T, Lu S-J, et al: Relationships between dorsolateral prefrontal cortex metabolic change and cognitive impairment in first-episode neuroleptic-naive schizophrenia patients. Medicine (Baltimore) 2017; **96**: e7228.

150. Iwata Y, Nakajima S, Plitman E, et al: Glutamatergic Neurometabolite Levels in Patients With Ultra-Treatment-Resistant Schizophrenia: A Cross-Sectional 3T Proton Magnetic Resonance Spectroscopy Study. Biol. Psychiatry 2019; **85**: 596–605.

151. Jakary A, Vinogradov S, Feiwell R, et al: N-acetylaspartate reductions in the mediodorsal and anterior thalamus in men with schizophrenia verified by tissue volume corrected proton MRSI. Schizophr. Res. 2005; **76**: 173–185.

152. Jessen F, Fingerhut N, Sprinkart AM, et al: N-acetylaspartylglutamate (NAAG) and N-acetylaspartate (NAA) in patients with schizophrenia. Schizophr. Bull. 2013; **39**: 197–205.

153. Jessen F, Scherk H, Träber F, et al: Proton magnetic resonance spectroscopy in subjects at risk for schizophrenia. Schizophr. Res. 2006; **87**: 81–88.

154. Häfner H, Maurer K, Ruhrmann S, et al: Early detection and secondary prevention of psychosis: facts and visions. Eur. Arch. Psychiatry Clin. Neurosci. 2004; **254**: 117–128.

155. Kegeles LS, Shungu DC, Anjilvel S, et al: Hippocampal pathology in schizophrenia: magnetic resonance imaging and spectroscopy studies. Psychiatry Res. 2000; **98**: 163–175.

156. Kim S-Y, Kaufman MJ, Cohen BM, et al: In Vivo Brain Glycine and Glutamate Concentrations in Patients With First-Episode Psychosis Measured by Echo Time-Averaged Proton Magnetic Resonance Spectroscopy at 4T. Biol. Psychiatry 2018; **83**: 484–491.

157. Kirtaş D, Karadağ RF, Balci Şengül MC, et al: 1H-magnetic resonance spectroscopy in first episode and chronic schizophrenia patients. Turk. J. Med. Sci. 2016; **46**: 862–871.

158. Klär AA, Ballmaier M, Leopold K, et al: Interaction of hippocampal volume and N-acetylaspartate concentration deficits in schizophrenia: a combined MRI and 1H-MRS study. NeuroImage 2010; **53**: 51–57.

159. Kraguljac NV, Morgan CJ, Reid MA, et al: A longitudinal magnetic resonance spectroscopy study investigating effects of risperidone in the anterior cingulate cortex and hippocampus in schizophrenia. Schizophr. Res. 2019; **210**: 239–244.

160. Lebedeva IS, Sidorin SV, Akhadov TA, et al: [Some structural and functional features of the dorsolateral prefrontal cortex and the corpus callosum genu and auditory information processing (P300) in healthy subjects and patients with juvenile schizophrenia]. Zh. Vyssh. Nerv. Deiat. Im. I. P. Pavlova 2012; **62**: 544–552.

161. Legind CS, Broberg BV, Mandl RCW, et al: Heritability of cerebral glutamate levels and their association with schizophrenia spectrum disorders: a 1[H]-spectroscopy twin study. Neuropsychopharmacol. Off. Publ. Am. Coll. Neuropsychopharmacol. 2019; **44**: 581–589.

162. Bartha R, Williamson PC, Drost DJ, et al: Measurement of glutamate and glutamine in the medial prefrontal cortex of never-treated schizophrenic patients and healthy controls by proton magnetic resonance spectroscopy. Arch. Gen. Psychiatry 1997; **54**: 959–965.

163. Başoğlu C, Cetin M, Oner O, et al: Comparison of right thalamus and temporal cortex metabolite levels of drug-naive first-episode psychotic and chronic schizophrenia in patients. Turk Psikiyatri Derg. Turk. J. Psychiatry 2006; **17**: 85–91.

164. Chiappelli J, Hong LE, Wijtenburg SA, et al: Alterations in frontal white matter neurochemistry and microstructure in schizophrenia: implications for neuroinflammation. Transl. Psychiatry 2015; **5**: e548.

165. Choe BY, Kim KT, Suh TS, et al: 1H magnetic resonance spectroscopy characterization of neuronal dysfunction in drug-naive, chronic schizophrenia. Acad. Radiol. 1994; **1**: 211–216.

166. Maier M, Mellers J, Toone B, et al: Schizophrenia, temporal lobe epilepsy and psychosis: an in vivo magnetic resonance spectroscopy and imaging study of the hippocampus/amygdala complex. Psychol. Med. 2000; **30**: 571–581.

167. Premkumar P, Parbhakar VA, Fannon D, et al: N-acetyl aspartate concentration in the anterior cingulate cortex in patients with schizophrenia: A study of clinical and neuropsychological correlates and preliminary exploration of cognitive behaviour therapy effects. Psychiatry Res. 2010; **182**: 251–260.

168. Stanley JA, Vemulapalli M, Nutche J, et al: Reduced N-acetyl-aspartate levels in schizophrenia patients with a younger onset age: a single-voxel 1H spectroscopy study. Schizophr. Res. 2007; **93**: 23–32.

169. Taylor R, Osuch EA, Schaefer B, et al: Neurometabolic abnormalities in schizophrenia and depression observed with magnetic resonance spectroscopy at 7 T. BJPsych Open 2017; **3**: 6–11.

170. Terpstra M, Vaughan TJ, Ugurbil K, et al: Validation of glutathione quantitation from STEAM spectra against edited 1H NMR spectroscopy at 4T: application to schizophrenia. Magma N. Y. N 2005; **18**: 276–282.

171. Théberge J, Williamson KE, Aoyama N, et al: Longitudinal grey-matter and glutamatergic losses in first-episode schizophrenia. Br. J. Psychiatry J. Ment. Sci. 2007; **191**: 325–334.

172. Tunc-Skarka N, Weber-Fahr W, Hoerst M, et al: MR spectroscopic evaluation of N-acetylaspartate’s T2 relaxation time and concentration corroborates white matter abnormalities in schizophrenia. NeuroImage 2009; **48**: 525–531.

173. Venkatraman TN, Hamer RM, Perkins DO, et al: Single-voxel 1H PRESS at 4.0 T: precision and variability of measurements in anterior cingulate and hippocampus. NMR Biomed. 2006; **19**: 484–491.

174. Ueno F, Nakajima S, Iwata Y, et al: Gamma-aminobutyric acid (GABA) levels in the midcingulate cortex and clozapine response in patients with treatment-resistant schizophrenia: A proton magnetic resonance spectroscopy (1 H-MRS) study. Psychiatry Clin. Neurosci. 2022; **76**: 587–594.

175. Cadenhead KS, Mirzakhanian H, Achim C, et al: Peripheral and central biomarkers associated with inflammation in antipsychotic naïve first episode psychosis: Pilot studies. Schizophr. Res. 2024; **264**: 39–48.

176. Chiappelli J, Savransky A, Ma Y, et al: Impact of lifetime stressor exposure on neuroenergetics in schizophrenia spectrum disorders. Schizophr. Res. 2024; **269**: 58–63.

177. Fan L, Zhang Z, Ma X, et al: Glutamate levels and symptom burden in high-risk and first-episode schizophrenia: a dual-voxel study of the anterior cingulate cortex. J. Psychiatry Neurosci. JPN 2024; **49**: E367–E376.

178. Koster M, van der Pluijm M, van de Giessen E, et al: The association of tobacco smoking and metabolite levels in the anterior cingulate cortex of first-episode psychosis patients: A case-control and 6-month follow-up 1H-MRS study. Schizophr. Res. 2024; **271**: 144–152.

179. León-Ortiz P, Rivera-Chávez LF, Torres-Ruíz J, et al: Systemic inflammation and cortical neurochemistry in never-medicated first episode-psychosis individuals. Brain. Behav. Immun. 2023; **111**: 270–276.

180. Wang M, Barker PB, Cascella NG, et al: Longitudinal changes in brain metabolites in healthy controls and patients with first episode psychosis: a 7-Tesla MRS study. Mol. Psychiatry 2023; **28**: 2018–2029.

181. Allam AEI, Reda AM, Eissa MAERSA, et al: Usefulness of combined pseudo-continuous arterial spin labelling and spectroscopic analysis in schizophrenic Egyptian population sample. Egypt. J. Radiol. Nucl. Med. 2024; **55**: 149.

182. Tandon R, Gaebel W, Barch DM, et al: Definition and description of schizophrenia in the DSM-5. Schizophr. Res. 2013; **150**: 3–10.

183. Kubota M, Takahata K, Matsuoka K, et al: Positron Emission Tomography Assessments of Phosphodiesterase 10A in Patients With Schizophrenia. Schizophr. Bull. 2023; **49**: 688–696.

184. Stanley JA, Daugherty AM, Richter Gorey C, et al: Basal glutamate in the hippocampus and the dorsolateral prefrontal cortex in schizophrenia: Relationships to cognitive proficiency investigated with structural equation modelling. World J. Biol. Psychiatry Off. J. World Fed. Soc. Biol. Psychiatry 2023; **24**: 730–740.

185. Woodcock EA, Arshad M, Khatib D, et al: Automated Voxel Placement: A Linux-based Suite of Tools for Accurate and Reliable Single Voxel Coregistration. J. Neuroimaging Psychiatry Neurol. 2018; **3**: 1–8.

186. Olbrich HM, Valerius G, Rüsch N, et al: Frontolimbic glutamate alterations in first episode schizophrenia: evidence from a magnetic resonance spectroscopy study. World J. Biol. Psychiatry Off. J. World Fed. Soc. Biol. Psychiatry 2008; **9**: 59–63.
